# A stretchable human lung‐on‐chip model of alveolar inflammation for evaluating anti‐inflammatory drug response

**DOI:** 10.1002/btm2.10715

**Published:** 2024-09-05

**Authors:** Clémentine Richter, Lorenz Latta, Daria Harig, Patrick Carius, Janick D. Stucki, Nina Hobi, Andreas Hugi, Paul Schumacher, Tobias Krebs, Alexander Gamrekeli, Felix Stöckle, Klaus Urbschat, Galia Montalvo, Franziska Lautenschläger, Brigitta Loretz, Alberto Hidalgo, Nicole Schneider‐Daum, Claus‐Michael Lehr

**Affiliations:** ^1^ Helmholtz Institute for Pharmaceutical Research Saarland Saarbrücken Germany; ^2^ Department of Pharmacy Saarland University Saarbrücken Germany; ^3^ AlveoliX AG, Swiss Organs‐on‐Chip Innovation Bern Switzerland; ^4^ ARTORG Center for Biomedical Engineering Research, Organs‐on‐Chip Technologies, University of Bern Bern Switzerland; ^5^ Vitrocell® Systems GmbH Waldkirch Germany; ^6^ Center for Thorax Medicine, Clinic Saarbrücken Saarbrücken Germany; ^7^ Section of Thoracic Surgery of the Saar Lung Center, SHG Clinics Völklingen Germany; ^8^ Department of Experimental Physics Saarland University Saarbrücken Germany; ^9^ Biophysics, Center for Integrative Physiology and Molecular Medicine (CIPMM), School of Medicine, Saarland University Homburg Germany; ^10^ Center for Biophysics, Saarland University Saarbrücken Germany

**Keywords:** 3R, acute lung injury, aerosolization, cytokine storm, immuno‐competent, lung‐on‐chip, microfluidic

## Abstract

This study describes a complex human in vitro model for evaluating anti‐inflammatory drug response in the alveoli that may contribute to the reduction of animal testing in the pre‐clinical stage of drug development. The model is based on the human alveolar epithelial cell line Arlo co‐cultured with macrophages differentiated from the THP‐1 cell line, creating a physiological biological microenvironment. To mimic the three‐dimensional architecture and dynamic expansion and relaxation of the air‐blood‐barrier, they are grown on a stretchable microphysiological lung‐on‐chip. For validating the in vitro model, three different protocols have been developed to demonstrate the clinically established anti‐inflammatory effect of glucocorticoids to reduce certain inflammatory markers after different pro‐inflammatory stimuli: (1) an inflammation caused by bacterial LPS (lipopolysaccharides) to simulate an LPS‐induced acute lung injury measured best with cytokine IL‐6 release; (2) an inflammation caused by LPS at ALI (air‐liquid interface) to investigate aerosolized anti‐inflammatory treatment, measured with chemokine IL‐8 release; and (3) an inflammation with a combination of human inflammatory cytokines TNFα and IFNγ to simulate a critical cytokine storm leading to epithelial barrier disruption, where the eventual weakening or protection of the epithelial barrier can be measured. In all cases, the presence of macrophages appeared to be crucial to mediating inflammatory changes in the alveolar epithelium. LPS induction led to inflammatory changes independently of stretch conditions. Dynamic stretch, emulating breathing‐like mechanics, was essential for in vitro modeling of the clinically relevant outcome of epithelial barrier disruption upon TNFα/IFNγ‐induced inflammation.

**Translational Impact Statement**

Organ‐on‐chips form a link between late preclinical research on animal models and clinical investigations on humans. The human in vitro model of pulmonary inflammation presented in this study is intended to predict the anti‐inflammatory effects of new potential drug candidates. To demonstrate this, human alveolar epithelial and immune cells were co‐cultivated in a dynamic lung‐on‐chip, inflamed, and treated with the known glucocorticoid Budesonide as proof of concept.

## 1 INTRODUCTION

Inhaled anti‐inflammatory drugs fall in the category of respiratory drugs, which have one of the highest attrition rates in clinical testing compared to other diseases.^1^ Most respiratory drugs fail in late clinical stages due to efficacy issues, highlighting the need for reliable proof of concept models for drug response to identify these failing candidates earlier and reduce future costs.^1^, ^2^ By using microfluidic lung‐on‐chips, which simulate the pulmonary micro‐environment more accurately than traditional static and 2‐dimensional in vitro models, the predictability of human drug response is expected to be more accurate.^3^, ^4^ To date, various complex microfluidic models have been used to investigate treatment options and efficacy against airway infections,^5^, ^6^ lung cancer,^7^ lung edema,^8^ and lung thrombosis,^9^ some including different cell types, medium flow, lateral stretch, or the possibility to replicate the air–liquid interface (ALI). In a future perspective, such complex in vitro models may provide reliable alternatives to animal models and facilitate the translation from pre‐clinical to clinical investigation, in line with the 3R principle of replacement, reduction, and refinement of animal experiments.^10^, ^11^, ^12^, ^13^, ^14^, ^15^ On the other hand, it should always be considered that in vitro models, as complex as they may be, only represent some limited aspects of the physiological state. Before being accepted alongside or as alternatives to animal experiments, the models, their read‐outs, and their correlation to human in vivo data need to be carefully evaluated and standardized.^16^, ^17^, ^18^ Additionally, the handling is often more complex than simpler in vitro models and the costs are higher, necessitating a thorough evaluation of effort and benefit.^2^, ^19^

Two main anatomical areas of the human lung can be discerned: the conducting airways, consisting mainly of bronchi of gradually decreasing diameter, and the respiratory area in the deep lung, formed by the alveoli. The main structure of the alveolar region is the very large (100–140 m^2^) and very thin (1–2 μm), but very tight air‐blood barrier, formed by endothelial cells, a basal membrane, and alveolar epithelial cells. On top of the epithelial cells, patrolling alveolar macrophages and the surfactant lining constitute the last line of immune defense against inhaled toxic particles before they can reach the bloodstream through the air‐blood barrier.^14^, ^20^, ^21^

**FIGURE 1:**
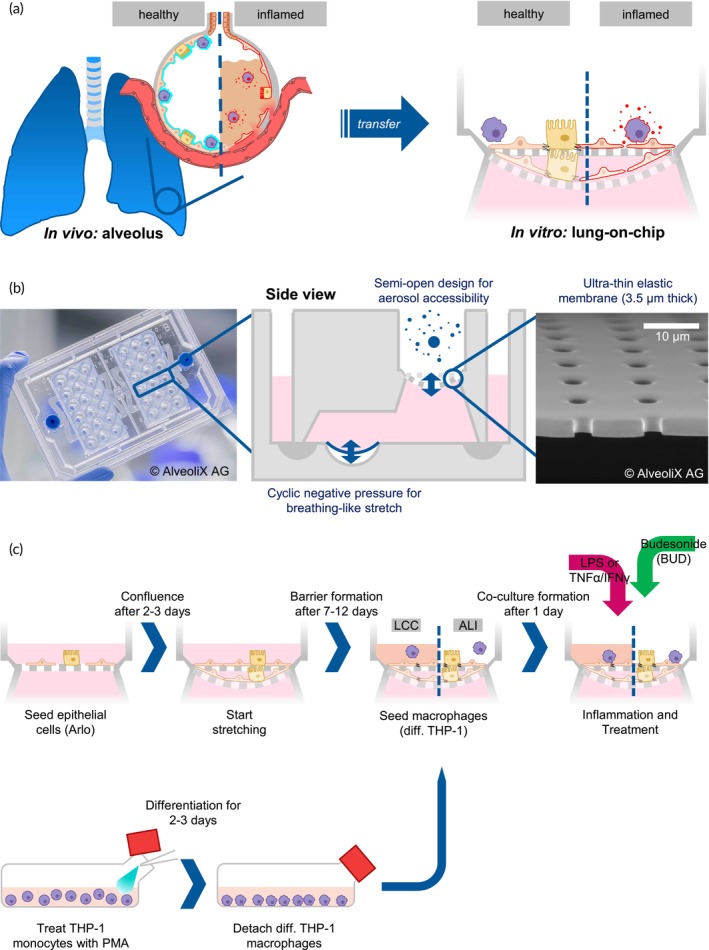
Chip and experimental design. (a) Experimental idea of transferring the inflamed alveolar state to an in vitro model on chip, creating a disease model that is sensible to anti‐inflammatory drugs. (b) Lung‐on‐chip design of the AX12. The AX12 is based on a 96‐well plate format, with two chips in each plate. Each chip contains six individual lung‐on‐chip units, with a central well separated horizontally by a flexible porous membrane on which the cells can be grown. The two wells on each side of the central well are the inlet and outlet to reach the basal compartment. A cyclic negative pressure applied at the bottom of the basal chamber deflects the diaphragm, and this deflection is transferred to the porous membrane with the cells. (c) Setup and experimental protocol for a stretchable microphysiological model of the human air‐blood barrier: First, epithelial cells (Arlo) are seeded on the porous membrane of the lung‐on‐chip. After the cells have attached and grown to confluence, the cells are subjected to stretch for the remaining time of the experiment. In parallel, THP‐1 monocytes are differentiated with 7.5 ng/mL PMA for 2–3 days. After barrier formation (checked by regular TEER measurement), co‐cultures with macrophage surrogates (differentiated THP‐1 cells) are set up and left to acclimate for 24 hours before inflaming the co‐cultures with either LPS or a combination of TNFα/IFNγ. Co‐cultures are treated with the anti‐inflammatory drug Budesonide two hours after inflammation.

This study aimed to develop an in vitro model of alveolar inflammation by incorporating multiple cell types and the option to include breathing‐like mechanical stretch. Additionally, compared to other microfluidic setups, the AX12 system from AlveoliX AG is the only one with the possibility to apply aerosols to the ALI combined with stretch.^22^, ^23^ Contrary to models mimicking the healthy state as used in toxicology/safety studies, models to evaluate drug response must display the disease state realistically and over a long enough time to measure the effects of the drug treatment. One main challenge is finding and maintaining this diseased state in between the healthy and dead states in vitro (Figure 1a). However, no basal medium flow can be included in this chip.

The model presented here is intended for the evaluation of anti‐inflammatory drug response for the treatment of alveolar inflammation. The structural and dynamic micro‐environment of the alveoli was recreated by the microfluidic chip AX12 (AlveoliX AG): Epithelial cells and macrophages are grown on the apical side of a flexible, porous membrane of biocompatible silicone, mimicking the structure of the air‐blood barrier with the unique possibility of combining lateral stretch with the deposition of aerosols directly on the air‐exposed apical side of the cells (Figure 1b).^3^, ^24^, ^25^ The flexibility of the membrane allows for the application of dynamic three‐dimensional lateral stretch which emulates the expansion and relaxation of the breathing alveolus^8^, ^26^ and may alter the inflammatory response or epithelial barrier integrity.^27^ It has been reported that mechanical stretch can positively influence epithelial cell proliferation,^2^ differentiation,^28^ migration,^29^ and increase the resistance against viral infections in vitro.^30^ To model the pulmonary air‐blood barrier, the human alveolar epithelial cell line Arlo^31^ was used, either alone or in co‐culture with monocyte‐derived macrophage‐like cells as surrogates for alveolar macrophages to investigate the differences in drug response in mono‐ and co‐cultures.^32^, ^33^ Similar to primary human alveolar epithelial cells (hAEpC), the Arlo cell line displays a very low paracellular permeability, mediated by tight intercellular junctions.^34^, ^35^ This particularly qualifies this cell line to investigate mechanisms of epithelial barrier disruption and restoration. To validate these results, some experiments have additionally been repeated with primary hAEpC. THP‐1 cells, which have long been used as a model for M1 macrophages,^36^, ^37^ especially in the context of acute alveolar inflammation,^33^, ^38^ were used as surrogates for alveolar macrophages. It was hypothesized that the implementation of mechanical stretch, aerosol exposure, and immune cells into the model would lead to a more realistic representation of alveolar (patho)physiology.^39^

LPS (bacterial lipopolysaccharides) was chosen as an inflammatory stimulus because it is widely used in mice^40^, ^41^, ^42^, ^43^ and in vitro^33^, ^44^, ^45^ to model acute lung injury. Alternatively, a combination of high doses of the human inflammatory cytokines TNFα (tumor necrosis factor‐alpha) and IFNγ (interferon gamma) was used to simulate a critical cytokine storm leading to epithelial barrier disruption,^18^ like what would be expected, e.g., in severe progress of COVID‐19 infection.^46^, ^47^, ^48^

Measuring inflammatory cytokines is an established read‐out in inflammatory models,^49^ whereas changes in the alveolar air‐blood‐barrier function may be monitored by measuring transepithelial electrical resistance (TEER).^50^ As a proof of concept to demonstrate the sensibility to anti‐inflammatory drugs and the restoration of the “healthy” state from such inflammatory changes, the anti‐inflammatory glucocorticoid budesonide (BUD) was used.^51^ Different protocols were needed to demonstrate the associated pathophysiological changes and their restoration by BUD, as LPS‐ and TNFα/IFNγ‐induced inflammations rely on different pathological mechanisms.

With the end goal of establishing protocols for more physiologically relevant lung disease models, it is important to understand the relevance and interplay between different physiological parameters (such as the role of macrophages, mechanical stretch, or drug deposition). It can be tested in this limited in vitro setup, whether a physiological factor is relevant, and if and how to implement it in the model. This study shows for example that while macrophages are crucial in all tested disease settings, the impact of mechanical stretch is most relevant in TNFα/IFNγ‐triggered cytokine storm.

## 2 MATERIALS AND METHODS

### 2.1 Cell culture methods

#### 2.1.1 Arlo cell line

The cell line “Arlo,” a monoclonal lentivirus‐immortalized epithelial cell line of the human deep lung, was developed in‐house and cultured according to a recent publication.^31^ For experiments on AX12, Arlo was seeded with a density of 4 × 10^5^ cells/cm^2^ in 70 μL.

#### 2.1.2 THP‐1 cell line

The THP‐1 cell line^36^ (No. ACC‐16, DSMZ (Deutsche Sammlung für Mikroorganismen und Zellkulturen)) was cultivated and differentiated according to a previously published protocol with minor changes.^38^ Briefly, 3 × 10^6^ THP‐1 cells in 3 mL were differentiated to macrophage‐like cells (dTHP‐1) with 7.5 ng/mL of PMA (phorbol 12‐myristate 13‐acetate, Sigma‐Aldrich PA585) in 10 mL RPMI (Roswell Park Memorial Institute) medium supplemented with 10% fetal calf serum (FCS) for two to three days (Figure S1).^52^ dTHP‐1 cells were washed twice with phosphate‐buffered saline (PBS, Sigma‐Aldrich D8537), incubated with 3 mL Accutase (Sigma‐Aldrich A6964) for 30 min at 37°C, and gently detached with a cell scraper (Greiner bio‐one 541070).

#### 2.1.3 Co‐cultures of Arlo and dTHP‐1 cell lines

**FIGURE 2:**
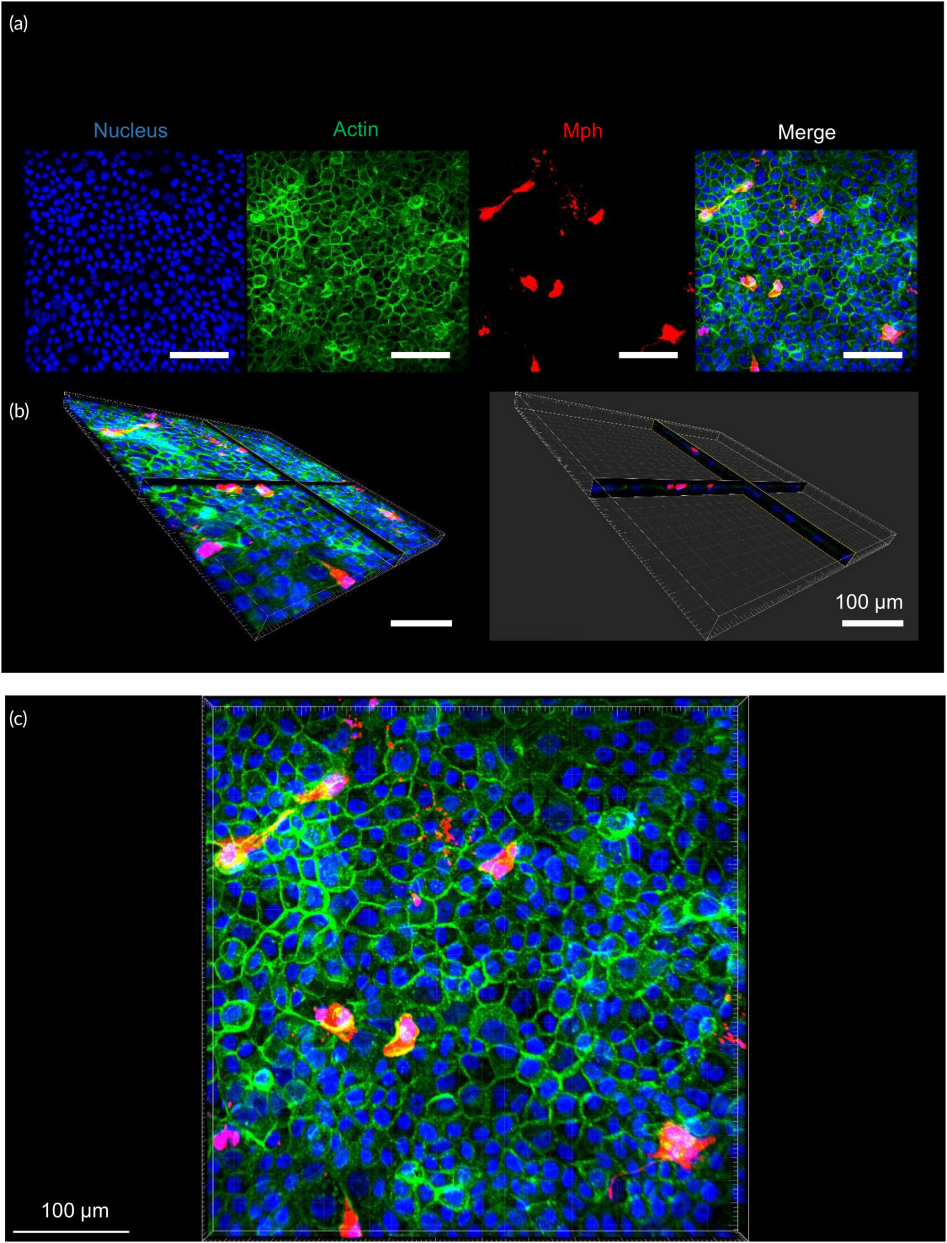
Confocal images of co‐culture characterization. (a) The image of the co‐culture shows the cell nuclei (stained with DAPI) in blue, the actin filaments (stained with Phalloidin) in green, and the whole macrophages (stained with Far Red Cell Tracer) in red. Macrophages are evenly distributed on top of the epithelial cells. (b) Cross‐section of the co‐culture shows the close contact between Arlo and dTHP‐1 cells. (c) Magnification of co‐culture image for better representation. All representative images.

Co‐cultures of Arlo and dTHP‐1 cells were set up according to a previous publication.^38^ In short, after TEER increase of Arlo cells, dTHP‐1 cells were seeded on top of the epithelial barrier with 2.4 × 10^5^ cells/cm^2^ in 70 μL (LCC) or 3 μL (ALI).^21^ After macrophage cell seeding, the cells were allowed to interact for 24 h before starting inflammation experiments (Figures 1c and 2, Video S1).^53^

#### 2.1.4 Human alveolar epithelial cells (hAEpC)

Primary hAEpCs were isolated according to a previously published protocol.^34^ The human tissue was provided by the Clinic Saarbrücken and the SHG Clinics Völklingen. The procedure and use of patient material are in accordance with the Helsinki Declaration of 1975 (revised 2008) and were permitted by the local ethics committee of the state of Saarland, Germany (May 21, 2019, reference number 113/19 and May 10, 2021, reference number 97/21). All patient materials were delivered with anonymized labels, ensuring patient privacy. The local ethics committee of the state of Saarland, Germany, has reviewed the patient consent forms as well. The cells were seeded and cultivated on chip with 3.5 × 10^5^ cells/cm^2^.

### 2.2 ^AX^Lung‐On‐Chip System

#### 2.2.1 AX12

The ^AX^Lung‐On‐Chip system (AlveoliX AG) has been previously described in detail (Figure 1b).^23^, ^54^ In brief, the ^AX^Lung‐On‐Chip System consists of the AX12 containing a porous ultra‐thin membrane, connected to the electro‐pneumatic control units (^AX^Exchanger and ^AX^Breather) through the ^AX^Dock. The ^AX^Exchanger is used for medium exchange, sampling of basolateral samples, and TEER measurements. The ^AX^Breather is applying cyclic 3‐dimensional stretch. All experiments were conducted according to manufacturer specifications.

#### 2.2.2 ALI and nebulizing

Arlo cultures were switched to ALI after the formation of a tight monolayer approx. on day 12 after seeding. Nebulization of LPS and BUD was performed using the Cloud AX12^26^, ^55^ (Vitrocell® Systems GmbH) with an Aeroneb® Lab Nebulizer^56^ (standard VMAD, 4.0–6.0 μm droplet diameter) connected to an Aerogen® USB controller according to supplier instructions. LPS was nebulized at a concentration of 907 μg/mL, and BUD at 33.5 μg/mL, both with 300 μL. The volume is given by the manufacturer's instructions, and the concentrations are calculated according to the measured deposition efficiency (Figure S5b).

### 2.3 Inflammation and treatment protocols

#### 2.3.1 Inflammation with LPS

LPS from *E. coli* O26:B6 (Sigma‐Aldrich L2762‐5MG) stock solution was prepared with 1 μg/mL in PBS, aliquoted, and frozen at −20°C until use. An LPS response curve (0.05, 0.5, and 5.0 μg/mL) was performed in mono‐cultures of dTHP‐1 cells to select the optimal concentration for further experiments (Figure S2).^57^, ^58^, ^59^ The rest of the experiments in mono‐ and co‐cultures were performed with 0.5 μg/mL LPS when in LCC (liquid‐covered conditions). In the case of ALI cultures on chip, LPS was nebulized using the Cloud AX12, with a deposition of 20 μg/cm^2^, corresponding to 0.5 μg/mL in LCC or 1,75 pg/macrophage, as calculated according to the measured deposition efficiency (Figure S5b).^60^, ^61^, ^62^, ^63^, ^64^, ^65^

#### 2.3.2 Inflammation with TNFα/IFNγ

TNFα (Sigma‐Aldrich H8916) stock solution was prepared with 1.0 μg/mL in PBS, aliquoted, and frozen at −20°C until use. IFNγ (Miltenyi Biotec 130‐096‐48) stock solution was prepared with 100 μg/mL in PBS, aliquoted, and frozen at −20°C. Inflammation with a combination of TNFα/ IFNγ^18^ was performed in LCC with assay concentrations of ~0.1 μg/mL each, corresponding to 96 ng/cm^2^ or 0.33 fg/macrophage for TNFα and 113 ng/cm^2^ or 0.39 fg/macrophage for INFγ.

#### 2.3.3 Treatment with BUD

BUD (Sigma‐Aldrich, Pharmaceutical Secondary Standard PHR1178) stock was suspended in 100% ethanol at 3 mg/mL. Cultures were treated with an assay concentration of 1 μM BUD apically in LCC.^66^, ^67^, ^68^ In the case of ALI cultures on chip, BUD was nebulized with the Cloud AX12, which corresponded to a deposition of 350 ng/cm^2^ or 1.5 pg/macrophage. To ensure reproducibility and avoid cross‐contamination, an output control was performed regularly, and the nebulizer was thoroughly cleaned according to manufacturer specifications.

### 2.4 Read‐outs

#### 2.4.1 Cytokine quantification

Released cytokines were measured via FACS (bead‐based fluorescence‐activated cell sorting) assay using Human Soluble Protein Flex Sets for IL‐6 (558276), TNFα (560112), and IL‐8 (558277) with the Human Soluble Protein Master Buffer Kit (558264, all BD Biosciences). All samples were taken 24 h after LPS inflammation.^42^, ^65^ An aliquot of 60 μL apical medium was centrifuged for 4 min at 300×g, and 55 μL supernatant was immediately frozen at −80°C. In the case of ALI cultures, LCC were re‐established 30 min before the end of the 24 h and sampled the same way as LCC cultures. All samples were thawed only once directly before performing the quantification.

The beads were sorted and analyzed with a BD LSRFortessaTM FACS (BD Biosciences). The data were analyzed with FCAP Array Version 3.0.1 for Windows (BD Biosciences).

#### 2.4.2 Methods for epithelial barrier measurement

TEER on AX12 was measured with an EVOM2 with an adapted range (World Precision Instruments 300523) and electrodes for 96‐well plates (World Precision Instruments STX100M). Raw resistance data were corrected for cell growth area with the following formula:

$$\text{TEER}\;\left[\Omega {\mathrm{cm}}^{2}\right]=\left(\mathrm{raw}\;\left[\Omega \right]-\text{blank}\;\left[\Omega \right]\right)\times \text{surface area}\;\left[{\mathrm{cm}}^{2}\right]$$

The value for the blank in the AX12 is 450 Ω, and the porous surface area is 0.071 cm^2^.

The p_app_ (apparent permeability) of the small molecule FluNa (Fluorescein sodium salt, Sigma‐Aldrich F6377) was determined according to a previous protocol with minor changes:^69^ The medium was exchanged for HBSS (Gibco Thermo Fisher Scientific Inc. 14025‐050) containing 10 μg/mL FluNa apically with or without 8 mM EDTA (ethylenediamine tetraacetic acid disodium salt dihydrate, Carl ROTH® 8043.1). 70 μL basal HBSS was sampled every hour and the missing volume was replaced with fresh HBSS for 7 h. The amount of FluNa in the samples was measured via fluorescence with Tecan Infinite 200Pro Photometer (Tecan, λ_ex_ = 485 nm; λ_em_ = 530 nm), and p_app_ [cm/s] was calculated according to the following equation:

$$p_{\mathrm{app}}=\frac{\mathrm{dQ}}{\mathrm{dt}}\left[\frac{\mathrm{\mu g}}{s}\right]\times \frac{1}{\text{surface area}\;\left[{\mathrm{cm}}^{2}\right]\times c_{\text{start}}\left[\frac{\mathrm{\mu g}}{\mathrm{mL}}\right]}$$

#### 2.4.3 Cell stress and cell death measurement

Cell stress and death on chip after the start of the application of stretch were measured with the RealTime‐Glo™ Annexin V Apoptosis and Necrosis Assay (Promega JA1011), adapted to smaller volumes to match the AX12. In short, 3 μL of each component of the assay kit was mixed with 900 μL medium. An aliquot of 30 μL of this mixture was added to the 70 μL medium in the apical compartment, for a total apical volume of 100 μL. Dead control cells were challenged with 10% dimethyl sulfoxide (DMSO, Sigma‐Aldrich D2438‐5X10ML).

Luminescence (1000 ms) and fluorescence (λ_ex_ = 485 nm; λ_em_ = 525 nm) were measured with a Tecan Spark Cyto 600 cell imager and plate reader (Tecan). Blanks were measured and subtracted from all values.

#### 2.4.4 Confocal microscopy

Macrophages were stained before seeding with Far Red Cell Tracer (Invitrogen C34564) according to the manufacturer's specifications. Co‐cultures with stained macrophages were not used in inflammation experiments.

Fixation and staining of all cultures were performed according to a previous publication with modifications to adapt it to macrophage‐epithelial co‐cultures:^70^ cultures were washed three times with PBS very gently to minimize loss of macrophages, fixated with 4% PFA (paraformaldehyde, Sigma‐Aldrich 30525‐89‐4) for 15 min at room temperature, washed again very gently three times with PBS, and kept under PBS at 4°C until staining (see Supporting Information).

Confocal images were taken with a confocal laser scanning microscope (Leica, Dmi8 Confocal Laser Scanning Microscope) with a 25x water immersion objective. Images were analyzed with Imaris Version 9.7.2 for Windows (Oxford Instruments).

#### 2.4.5 RNA sequencing and analysis

RNA was harvested by incubation in lysis buffer (RLT Buffer, Qiagen 79216) from the basal and apical side for 5 min. Two wells were pooled for one sample, and a total of 6 samples out of three independent passages for Arlo or patients for hAEpC were collected. Isolation of RNA was performed with the RNeasy Micro Kit (Qiagen 74004) and the RNase‐Free DNase Set (Qiagen 79254) according to the manufacturer's instructions. Samples with guanidin salt contaminations were additionally cleaned with the Monarch® RNA Cleanup Kit (New England Biolabs T2030L) to achieve the minimum requirements for sequencing (500 ng total RNA, RQN >8; 260/280 ratio >1.8 and 230/260 ration <1.8, respectively).

Sequencing was performed by strand‐specific mRNA sequencing. mRNA library was prepared with the NEB Next Ultra II Directional RNA Library Prep Kit (New England Biolabs E7765). Sequencing was performed on NovaSeq 6000, PE50 (2× 50 bp) with 30 mio NGS reads per sample and 800 mio cluster flow cell output.

Fastq data were analyzed with a preset pipeline using RNAdetector^71^ running in a docker container. Star alignment with feature counts for read summarizing was chosen as the alignment algorithm.^72^ The gene counts table was normalized for inherent systematic or experimental biases using the Bioconductor package edgeR. A complete analysis summary with a run log can be found in the Data S1. Fastq files are deposited at the RADAR portal and can be downloaded upon request (doi.org/10.22000/nfYyspONBoAAnRGx and doi.org/10.22000/xSdEBCHTPOQlmpBi).

### 2.5 Statistics

Numerical data are presented as mean ± standard deviation. Graphs were created with GraphPad Prism Version 9.5.0. To compare the statistical significance of the results, one‐way ANOVA with subsequent Tukey's multiple comparisons were used. The statistical thresholds for p values were set as follows: 0.12 (ns), 0.033 (*), 0.002 (**), <0.001 (***), according to NEJM (The New England Journal of Medicine) policies.^73^

All experiments were performed at least three independent times, with the exact number of replicates specified in the corresponding figure legends.

A more detailed version of the Materials and Methods section can be found in the Supporting Information.

## 3 RESULTS

### 3.1 LPS‐induced acute lung injury on chip

#### 3.1.1 Inflammation with LPS and treatment with BUD in LCC

The inflammatory settings with LPS and treatment with BUD (Figures S2 and S3) were transferred to chip and epithelial mono‐cultures compared to macrophage‐epithelial co‐cultures by keeping the previously established parameters (0.5 μg/mL LPS and 1 μM BUD 2 h after LPS). IL‐6 and TNFα release into the apical supernatant was measured 24 h after inflammation with LPS. For comparability, all doses, number of macrophages, and volumes were kept the same as in the macrophage mono‐cultures.

**FIGURE 3:**
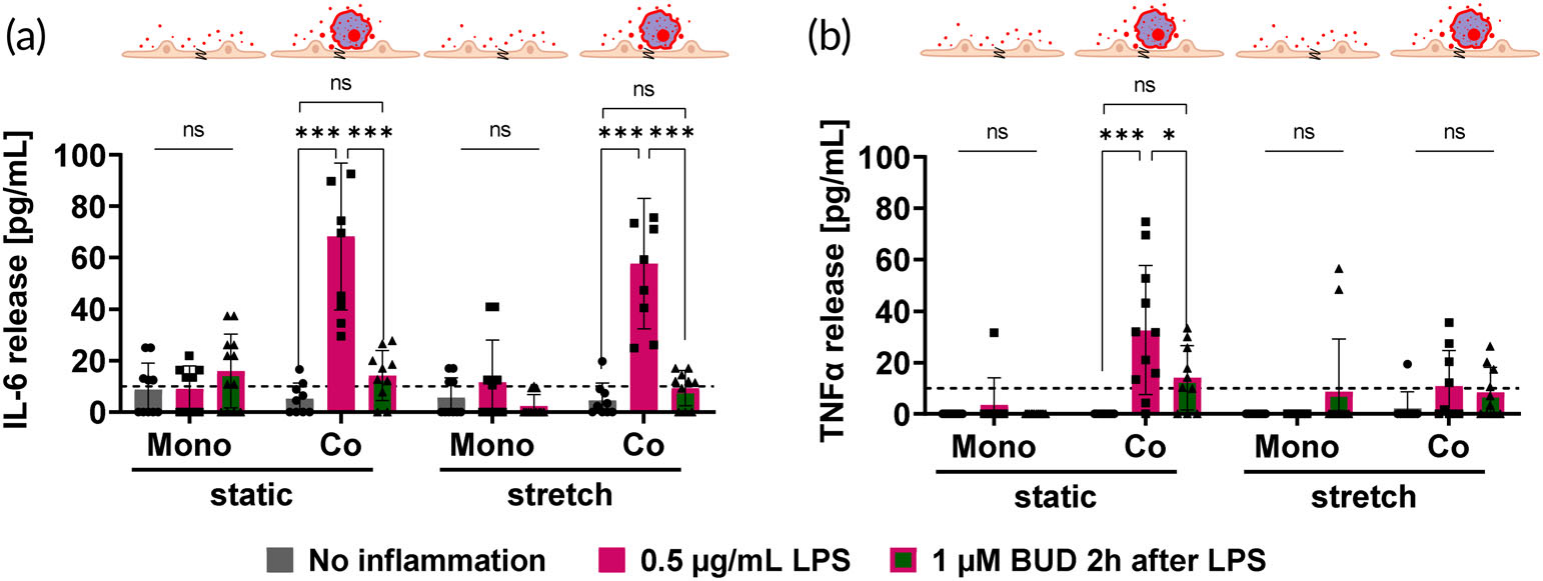
LPS‐induced acute lung injury model on chip in LCC. Inflammation on chip in epithelial mono‐culture and macrophage‐epithelial co‐culture showing cytokine release (IL‐6 in (a and TNF‐α in b) after inflammation and treatment comparing static and dynamic conditions. The dashed line represents the lower limit of quantification (LLOQ) of the assay. In general, released amounts of TNFα are lower compared to IL‐6. For almost all epithelial mono‐cultures, cytokine IL‐6 and TNFα release are below the LLOQ. IL‐6 shows a strong increase of release after inflammation with 0.5 μg/mL LPS and a significant reduction of release after BUD treatment two hours after inflammation (*n* = 9–12 out of 3–4 independent experiments).

In epithelial mono‐cultures on chip, cytokine IL‐6 and TNFα release were in most cases below the lower limit of quantification (LLOQ) of the assay and consequently, no effect of BUD could be observed either (Figure 3). The presence of macrophages in macrophage‐epithelial co‐cultures increased the cytokine release to measurable levels, pointing to the crucial role of macrophages to model LPS‐induced acute lung injury on chip. In both static and stretch conditions, an increase in cytokine release after LPS inflammation and decreased release after treatment with BUD could be measured (Figure 3).

#### 3.1.2 Inflammation with LPS and treatment with BUD at ALI

**FIGURE 4:**
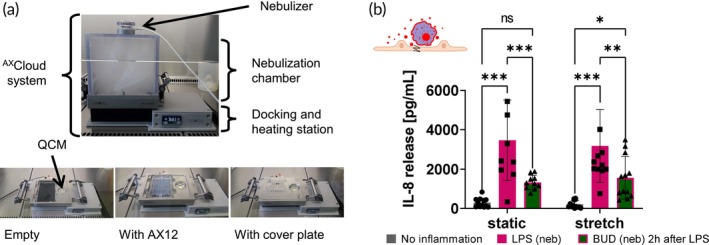
LPS‐induced acute lung injury model at ALI with nebulization of LPS and BUD. (a) Pictures of the Cloud AX12 deposition device. The Cloud AX12 consists of a heated base module, which serves as the docking station for the AX12. The AX12 is secured with the cover plate, covering the inlet and outlet wells, and leaving only the apical wells open for aerosol deposition, to ensure that only the cell‐bearing compartments are exposed to the aerosol. On top sits the tightly fitted aerosol chamber with the nebulizer as the aerosol source. (b) Inflammation on chip at ALI in macrophage‐epithelial co‐culture showing chemokine IL‐8 release comparing static and dynamic conditions. IL‐8 shows a strong increase of release after inflammation with 0.5 μg/mL LPS and significant reduction after Budesonide treatment two hours after inflammation (*n* = 10–13 out of 4 independent experiments).

To increase the physiological relevance of the LPS‐induced acute lung injury protocol, the macrophage‐epithelial co‐culture was adapted to ALI conditions, allowing exposure to LPS and BUD as aerosols using the Cloud AX12 (Figure 4a). Aerosol deposition can be monitored in real time using a quartz crystal microbalance (QCM) placed within the nebulization chamber (Figure S5, Video S2).

Both LPS and BUD were aerosolized and apical chemokine IL‐8 release was measured after 24 h. Comparable to the results in LCC, co‐cultures of Arlo and dTHP‐1 at ALI can be inflamed with nebulized LPS (increased chemokine release) and subsequently treated with BUD (decreased chemokine release), with no difference between static and stretch conditions (Figure 4b).

### 3.2 Characterization of epithelial cells on chip

#### 3.2.1 Effect of stretch on epithelial barrier formation

**FIGURE 5:**
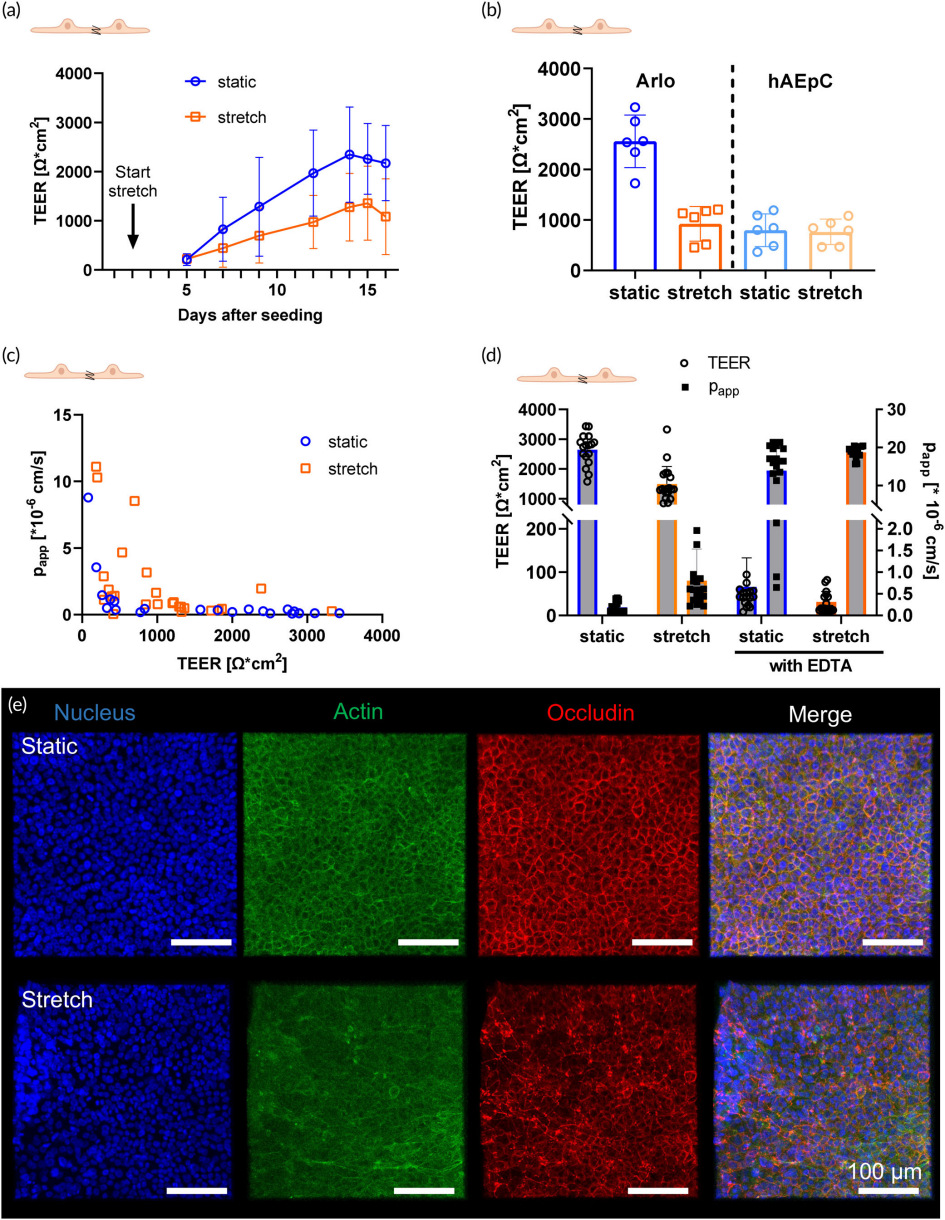
Characterization of microphysiological alveolar epithelial barrier in static and stretch conditions. (a) TEER development of Arlo on chip over time in static and stretch conditions. Breathing dynamics are applied from day 2 on (*n* = 60–64 out of 6 independent experiments). (b) TEER values after 6–7 days in culture comparing the plateau values in static and stretch conditions for Arlo and hAEpCs. Stretch lowers TEER values of Arlo to the more physiological level of hAEpC (*n* = 6 out of 3 independent experiments). (c) Correlation of TEER values (electrical resistance) to apparent permeability of small‐molecule fluorescein (p_app_) in Arlo. High TEER correlates with low permeability in both static and stretch conditions. Experiments were carried out at different time points to include data points with low barrier stability (26–29 single values, out of 5 independent experiments). (d) TEER and p_app_ (Fluorescein) data with and without EDTA treatment in Arlo. EDTA complexes Ca^2+^ ions, thereby reversibly opening the tight junctions in the epithelial layer. After EDTA treatment, a lower TEER correlates with increased permeability in both static and stretch conditions (*n* = 16–19 out of 4 independent experiments). (e) Confocal images of fixated Arlo cells on chip in static and stretch conditions. Cell nuclei are stained in blue with DAPI, actin filaments in green with Phalloidin, and tight junctions in red with an Occludin‐specific antibody. While still present in stretch conditions, actin filaments, and tight junctions are more organized in static conditions. The scale bar represents 100 μM. Representative images from several repeated experiments.

Cyclic stretch was applied as soon as the epithelial cells grew confluent on day 2 for the whole duration of the experiment, leading to generally lower TEER values in stretch conditions (Figure 5a). While weaker after stretch, the epithelial barrier was still tight (> 500 Ω/cm^2^) and stable over two weeks of culture in both conditions. The lower TEER values of Arlo in stretch conditions after 6–7 days are similar to the TEER values of hAEpC in both static and stretch conditions (Figure 5b).

High TEER values of Arlo could be correlated to low permeability of small molecule FluNa, while the p_app_ of FluNa increased with lower TEER (Figure 5c). Adding EDTA as a chelator of divalent cations to open the tight junctions led to lower TEER and higher p_app_, confirming the presence of functional tight junctions (Figure 5d). Less organized and more delocalized actin fibers (green, cytoskeleton) and occludin (red, tight junction protein) might in part explain the lower TEER values in stretch conditions (Figure 5e).

#### 3.2.2 Short‐term stretch effect

**FIGURE 6:**
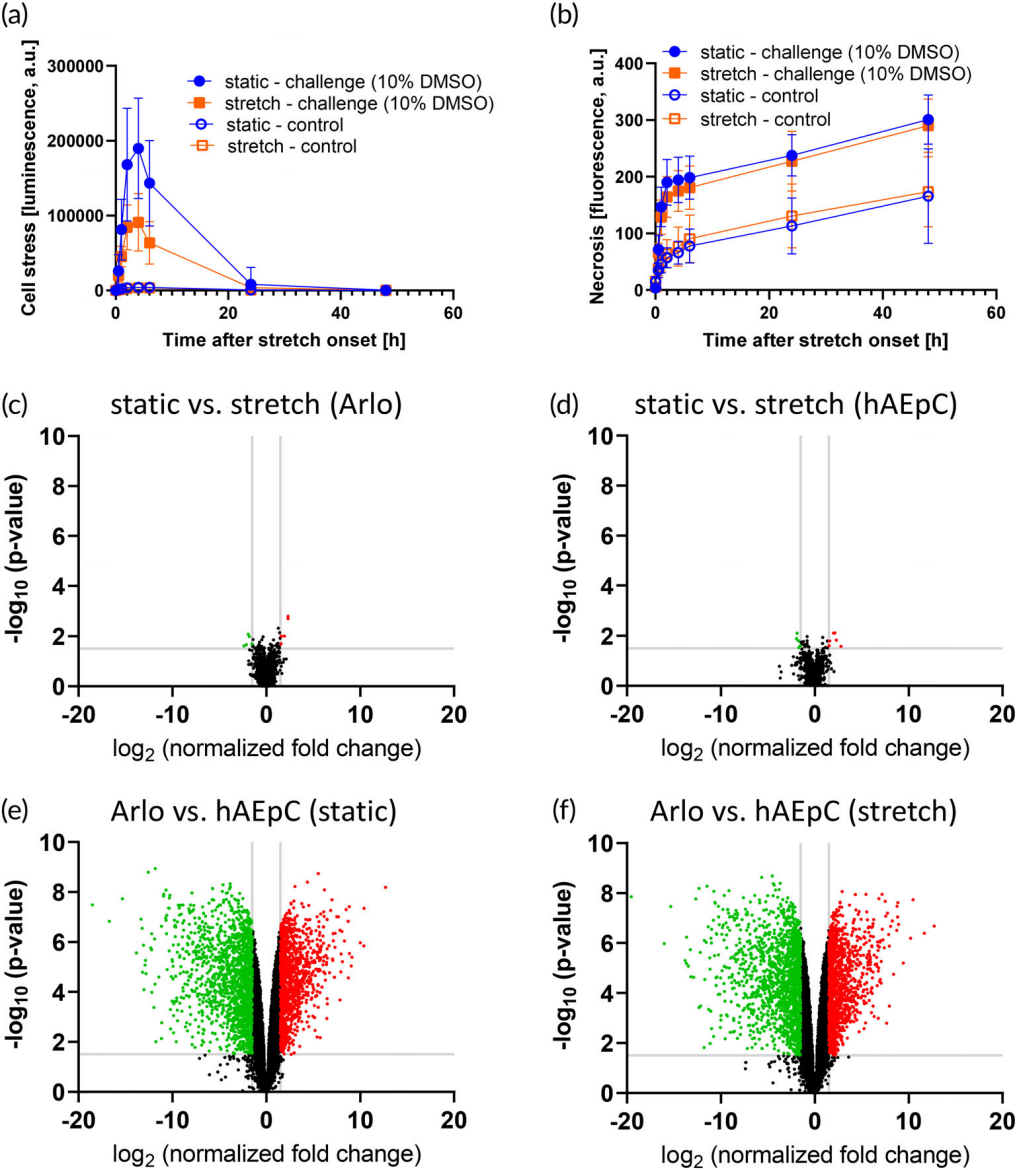
Investigation of stretch effect. (a) Cell stress measurement directly after applying stretch on day 2 of culture. In healthy conditions, no cell stress and no difference between static and stretch conditions can be observed. Upon adding 10% DMSO as a challenge, cells in stretch conditions seem more resistant to the challenge (*n* = 15–23 out of 3–4 independent experiments). (b) Cell death measurement directly after applying stretch on day 2 of culture. Stretch does not negatively affect cell death compared to static conditions (*n* = 15–23 out of 3–4 independent experiments). (c) RNA‐seq volcano plot of Arlo on chip depicting the differences between static and stretch conditions (*n* = 6 out of 3 independent experiments). (d) RNA‐seq volcano plot of hAEpCs on chip depicting the differences between static and stretch conditions (*n* = 6 out of 3 independent experiments). (e) RNA‐seq volcano plot of static conditions depicting the differences between Arlo and hAEpC (*n* = 6 out of 3 independent experiments). (f) RNA‐seq volcano plot of stretch conditions depicting the differences between Arlo and hAEpC (*n* = 6 out of 3 independent experiments).

To investigate potential short‐term effects caused by the onset of stretch dynamics, cell stress and cell death were measured and compared to cells additionally challenged by the addition of 10% DMSO (Figure 6a,b). Without the addition of DMSO, no cell stress was observed in either static or stretch conditions (Figure 6a). Like cell stress, no increased cell death upon stretch was observed (Figure 6b). After challenge with DMSO, no more cell growth or epithelial barrier formation was observed (Figure S6).

#### 3.2.3 Long‐term stretch effect

RNA of Arlo and hAEpC was collected after 6–7 days of culture (corresponding to 4–5 days of ongoing cyclic stretch) and sequenced. Direct comparison of Arlo in static and stretch conditions displayed no significant differences in the transcriptome (Figure 6c), as shown by false discovery rate (FDR) > 1.0 (Data S1). The same was found also for hAEpC under static and stretch conditions (Figure 6d).

When comparing Arlo and hAEpC in static conditions (Figure 6e) or in stretch conditions (Figure 6f), the individual expression profiles of the respective cell types were well preserved in either condition.

### 3.3 TNFα/IFNγ‐induced cytokine storm on chip

**FIGURE 7:**
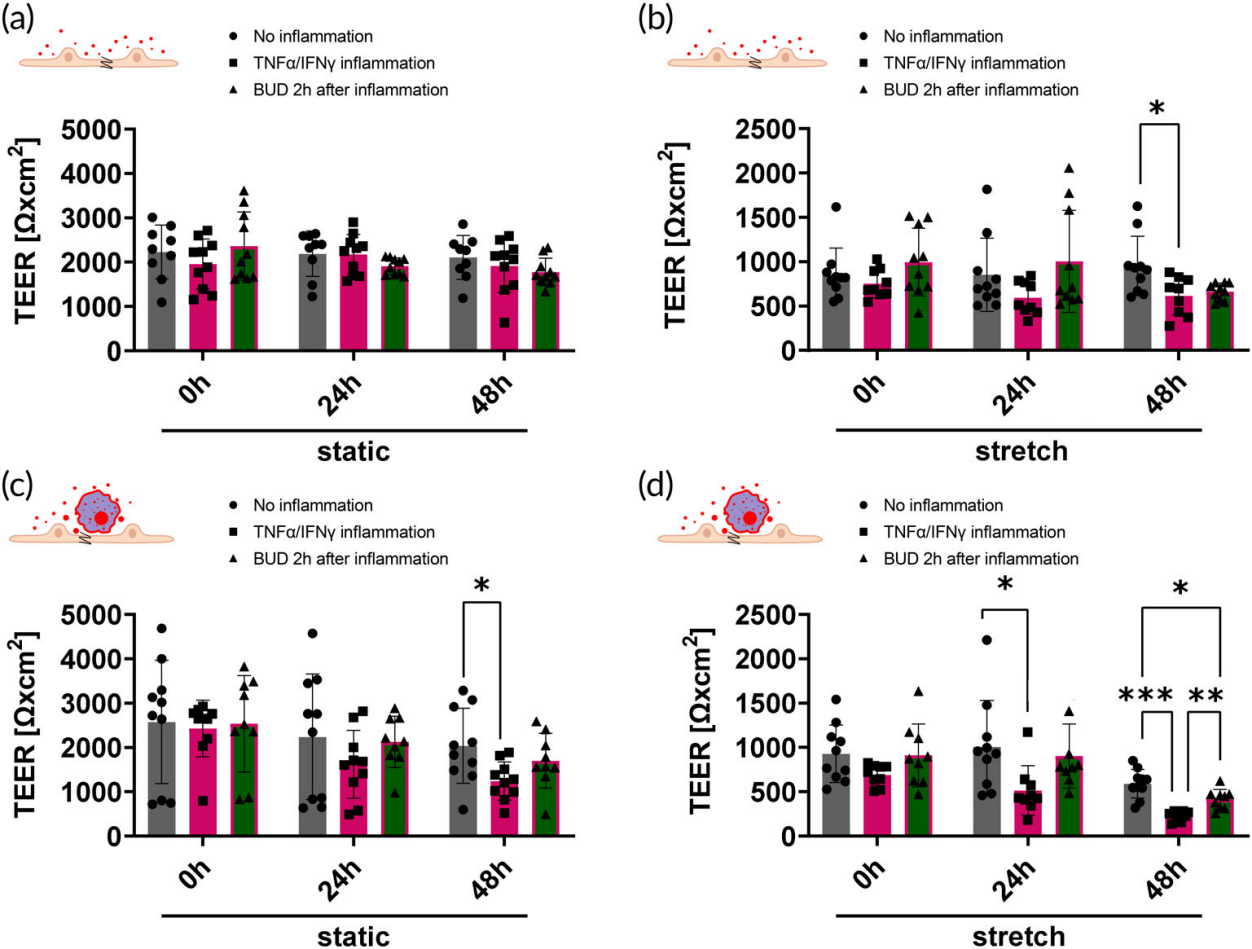
Inflammation of epithelial barrier on chip with a combination of TNFα/IFNγ and treatment with BUD showing improved disease modeling with applied stretch. (a) TEER changes of Arlo mono‐culture on chip in static and stretch conditions. Only in stretch conditions after 48 h can a slight decrease in barrier stability be measured. No effect of the Budesonide can be measured in the epithelial mono‐culture (*n* = 9–11 out of 3–4 independent experiments). (b) TEER changes of co‐culture of Arlo with diff. THP‐1 on chip in static and stretch conditions. In static conditions, a slight decrease in barrier stability can be measured after 48 h. In stretch condition, barrier weakening occurs already after 24 h and the protective effect of Budesonide can be measured after 48 h (*n* = 9–10 out of 3–5 independent experiments).

In epithelial mono‐cultures (Figure 7a,b), a weak disruption of the epithelial barrier could be measured after 48 h of inflammation in stretch conditions. Similarly, the co‐culture with macrophages in static conditions demonstrated a weak TNFα/IFNγ effect after 48 h (Figure 7c). However, the combined presence of macrophages and stretch conditions led to a weakened epithelial barrier already after 24 h of inflammation, causing a significant drop in TEER. Treatment with BUD 2 h after inflammation restored the epithelial barrier after 48 h in the co‐culture when stretch was applied (Figure 7d).

## 4 DISCUSSION

### 4.1 LPS‐induced acute lung injury on chip

#### 4.1.1 Inflammation with LPS and treatment with BUD in LCC

To investigate (1) the in vitro relevance of the immune cells and (2) the relevance of the dynamic environment of a breathing alveolus,^54^ mono‐cultures of Arlo and co‐cultures with dTHP‐1 macrophages were inflamed with LPS and treated with BUD in static and stretch conditions.

The presence of macrophages as the main immune cell component in the deep lung was crucial for measuring inflammatory signals by cytokine release after LPS inflammation in the in vitro alveolus model on chip (Figure 3). It is reported that the human lung epithelium does not express toll‐like receptor 4 (TLR4) and its associated proteins CD14 and MD‐2 for LPS recognition.^74^, ^75^ This is reflected by the poor response to LPS in epithelial mono‐cultures in this study, in most cases with cytokine releases below the LLOQ (Figure 3). Also, no epithelial barrier disruption could be detected after induction with solely LPS (Figure S4).

The application of stretch to mimic breathing motion within the alveoli was achieved by using the ^AX^Lung‐On‐Chip System (Figure 1b).^54^ In this protocol of LPS‐induced acute lung injury, stretch does not seem to impact the severity of the inflammation or the effect of the BUD treatment in the macrophage‐epithelial co‐cultures (Figure 3). This leads to the conclusion that the LPS‐induced inflammation is mainly macrophage‐driven and that the stretch does not seem to affect the macrophages or macrophage‐epithelial cellular cross‐talk in this setup. Previous investigations with murine, rat, and human macrophages showed very different effects of stretch on inflammation^76^: depending on the cell type, setup, and inflammatory trigger, either increase,^77^, ^78^ or decrease,^79^ or no effect^80^, ^81^ on cytokine release could be observed. However, all these studies investigate macrophages in mono‐cultures, while the setup proposed here is based on a co‐culture with epithelial cells. Adding this additional layer of physiological parameters might help to clarify the actual effect of stretch on macrophages and their cross‐talk with epithelial cells.

#### 4.1.2 Inflammation with LPS and treatment with BUD at ALI

The Cloud AX12 (Figure 4a) is specifically adapted to the AX12 and was used to nebulize both the proinflammatory mediator LPS and the anti‐inflammatory treatment BUD directly on top of the cells. An initial check of deposition efficiency allowed for the calculation of comparable doses of LPS and BUD to the previous experiments in LCC (Figure S5b).^15^

Under ALI conditions, in vitro models can exhibit very different cytokine and chemokine releases compared to their LCC counterparts.^82^ The same could be observed here, where the apical release of IL‐6 and TNFα was too low to be measured (data not shown). This might be attributed to both physical (e.g. dissolution, agglomeration, and concentration gradients) and biological effects (different morphology and cell physiology under LCC and ALI). The most important differences between LCC and ALI are cell differentiation, substance deposition rates, and dissolution rates.^82^ However, the positive drug effect of nebulized BUD could be demonstrated by measuring the release of the chemokine IL‐8 (Figure 4b). In the future, it might be interesting to add surfactant (including surfactant proteins)^83^, ^84^ in the ALI setup to investigate the effect on inflammatory processes,^85^ as it has already been described for infection dynamics.^5^ To our knowledge, this study represents the first microfluidic model investigating human anti‐inflammatory drug response after aerosolized drug application.

### 4.2 Characterization of epithelial cells on chip

#### 4.2.1 Effect of stretch on epithelial barrier formation

It is in accordance with previous findings that the TEER values of epithelial cells are lower in stretch conditions compared to static conditions on chip (Figure 5a).^8^, ^22^, ^86^, ^87^, ^88^ However, the TEER of primary cells was not affected by stretch, while the reduced TEER of Arlo was comparable to hAEpC (Figure 5b). Especially when using a cell line forming a very tight epithelial barrier such as Arlo, an overestimation of epithelial barrier strength under static conditions may be possible and, therefore, stretch conditions enable the induction of a more physiologically relevant epithelial barrier functionality, which is essential for in vitro modeling.

The observed delocalization of occludin and actin in the epithelial cells subjected to stretch (Figure 5e) confirm earlier observations that mechanical forces on various epithelia induce changes in the actin cytoskeleton and remodeling of tight junctions, leading to increased paracellular permeability and peri‐junctional actin levels.^89^, ^90^ Stretch also decreases ZO‐1 and occludin protein levels at tight junction sites, causing cytoskeletal rearrangements that disrupt the uniform localization of tight junction proteins along cell–cell junctions, ultimately disrupting epithelial barrier function.^91^ It has been shown in the intestinal Caco‐2 cell line that such effects on the tight junctions can be caused by a mechanism dependent on JNK2 (a MAP kinase associated with cell stress), c‐Src (a tyrosine kinase phosphorylating junction proteins amongst others), and MLCK (a kinase regulating the structure of actin filaments).^92^

#### 4.2.2 Short‐term stretch effect

Measuring cell stress and cell death directly after initiating the stretch revealed a good adaptation of Arlo to the mechanical stimulus. Neither increased cell stress nor death was measured by outer leaflet phosphatidylserine and free DNA (Figure 6a,b). While changes in epithelial barrier function (TEER), cytoskeleton, and tight junction organization could be observed with Arlo grown on chip in stretch conditions (Figure 5), the lack of cell stress or lack of increased cell death in the first hours after starting stretch highlighted the good short‐term adaptation of Arlo to chip culture and cyclic stretch. This concurs with observations in the context of VILI (ventilator‐induced lung injury), where it has been shown that short‐term and high mechanical stress (37% stretch) caused by high‐tidal‐volume mechanical ventilation leads to increased epithelial barrier permeability and lung edema, whereas lower stress (12% or 25% stretch) does not lead to any changes on the physiological level.^86^, ^87^, ^88^, ^93^, ^94^, ^95^ The mechanisms for VILI include both macroscopic structural damage^96^ and strong inflammation at the cellular level, caused among others by increased oxidative stress.^97^

#### 4.2.3 Long‐term stretch effect

The stretch was applied as soon as the epithelial cells grew confluent and it was maintained for the whole duration of the experiment (Figure 5a). RNA sequencing was performed after epithelial barrier formation to screen for any long‐term changes after stretch in control conditions, such as cell stress, cell differentiation, and junction formation. In healthy conditions, the long‐term effects of mechanical stretch seem to exclude changes in the transcriptome (Figure 6c,d), and prolonged mechanical stretch does not appear to exert any significant measurable influence on the transcriptome. The differences between static and stretch conditions affect functional parameters (TEER, p_app_, and cellular morphology, Figure 5), but are not reflected in the transcriptomic data. Therefore, the functional changes might be regulated at translational and posttranslational protein levels, for which further investigation would be very promising. Both primary cells and Arlo demonstrate the same high degree of adaptability to prolonged mechanical stretch, as no stress response‐related pathways were upregulated.

### 4.3 TNFα/IFNγ‐induced cytokine storm on chip

A cytokine storm was initated by inflaming the cells with high doses of TNFα and IFNγ and treated with BUD 2 h after inflammation (Figure 7). Contrary to previous publications, where the use of starving medium (devoid of FCS and hydrocortisone) was needed to induce a reduction in TEER after inflammation with TNFα/INFγ,^18^, ^31^ this setup on chip could be operated with the complete medium. The presence of macrophages to multiply inflammatory signaling^98^, ^99^, ^100^, ^101^, ^102^ in combination with stretch (Figure 7d) provided the best conditions for disease modeling and drug effect investigation, demonstrated by strong epithelial inflammation with epithelial barrier disruption, as well as epithelial barrier protection after BUD treatment. This concurs with previous findings that using a chip system with a stretchable membrane increases the sensibility of the system to inflammatory triggers, thereby creating a more sensitive measuring tool that is potentially physiologically more relevant.^8^, ^22^, ^26^

## 5 CONCLUSION

This study describes different setups to study inflammatory lung diseases and the effects of anti‐inflammatory drugs in vitro, based on the human alveolar epithelial cell line Arlo and dTHP‐1 macrophages cultivated on a microfluidic chip which imitates lateral stretch during breathing motion.

Under appropriate conditions, co‐cultures of Arlo with macrophages provide characteristic inflammatory read‐outs, but also respond to treatment with BUD. The setups were (1) LPS‐induced acute lung injury in LCC with cytokine measurement, (2) LPS‐induced acute lung injury at ALI after aerosolization of LPS and BUD with chemokine measurement, and (3) TNFα/IFNγ‐induced cytokine storm with TEER measurement.

The LPS‐induced acute lung injury on chip was not feasible with epithelial mono‐cultures, which demonstrates the importance of the presence of macrophages. Increased cytokine/chemokine release after inflammation could be prevented by BUD, also at ALI when delivered as an aerosol. However, the protocol for LPS inflammation could not emulate the most critical clinical outcome, the lung barrier disruption. The TNFα/IFNγ‐induced cytokine storm on chip provided the most prominent clinically translatable outcome, by inducing alveolar epithelial disruption, and therapeutic response to BUD when both macrophages and stretch were present, emphasizing the importance of such a dynamic micro‐environment for mimicking the human air‐blood‐barrier when it comes to disease modeling.

These approaches could be used in the future to develop novel in vitro models that may be validated to serve as predictive tools for developing new pulmonary anti‐inflammatory therapies.

## AUTHOR CONTRIBUTIONS

Clémentine Richter: Conceptualization, Formal Analysis, Investigation, Methodology, Project Administration, Visualization, Writing (original draft). Lorenz Latta: Conceptualization, Formal Analysis, Investigation, Supervision, Writing (original draft). Daria Harig: Investigation, Writing (review and editing). Patrick Carius: Conceptualization, Writing (review and editing). Janick D. Stucki: Conceptualization, Resources, Writing (review and editing). Nina Hobi: Conceptualization, Funding Acquisition, Resources, Writing (review and editing). Andreas Hughi: Methodology, Resources, Writing (review and editing). Paul Schumacher: Methodology, Resources, Writing (review and editing). Tobias Krebs: Methodology, Funding Acquisition, Resources, Writing (review and editing). Alexander Gamrekeli: Resources, Writing (review and editing). Felix Stöckle: Resources, Writing (review and editing). Klaus Urbschat: Resources, Writing (review and editing). Galia Montalvo: Methodology, Investigation, Writing (review and editing). Franziska Lautenschläger: Methodology, Writing (review and editing). Brigitta Loretz: Methodology, Supervision, Writing (review and editing). Alberto Hidalgo: Project Administration, Supervision, Writing (review and editing). Nicole Schneider‐Daum: Conceptualization, Funding Acquisition, Project Administration, Supervision, Writing (review and editing). Claus‐Michael Lehr: Conceptualization, Project Administration, Supervision, Writing (review and editing).

## FUNDING INFORMATION

This project was funded by the Eureka Eurostars program under the number E!12977—AIM4DoC (Advanced Inhalation Model for Drug Discovery on Chip) and internal funding label 01QE1912C.

## CONFLICT OF INTEREST STATEMENT

Some authors of this study are employed by the companies and institutions disclosed on the title page of this manuscript. AH, JS, and NH are employed by AlveoliX AG. JS and NH are minor shareholders of AlveoliX AG. PS is an employee, and TK is an employee and a shareholder of Vitrocell® Systems GmbH. All other authors declare that they have no conflicts of interest.

## Supporting information


**Data S1:** Differential expression analysis report

**Data S2:** Supporting Information

**Suppl. Video S1:** Aggregation and mobility of dTHP‐1 macrophages in co‐culture with Arlo. Arlo was grown to confluence on Fluorodishes (FD35‐100, World Precision Instruments). THP‐1 cells were differentiated and stained with CellTrace Far Red (C34564, Invitrogen) according to supplier protocol. After 24 h of acclimatization, co‐cultures were inflamed, treated, and analyzed under the microscope. Bright‐field and fluorescent images were recorded using an EMCCD camera (Andor Technology, Belfast, Northern Ireland, UK) with a physical pixel size of 0.65 μm, mounted on a Nikon Eclipse Ti epifluorescent microscope, at a 10X magnification and 0.45 numerical aperture (Nikon Plan Apo objective) over 20 h with a frame rate of 2 min (1 s video corresponds to 20 min in real‐time). The cells were kept at a constant atmosphere of 37°C and 5% CO_2_ (Okolab, Pozzuoli NA, Italy) during the entire experiment. Representative videos out of 6 replicates.

**Suppl. Video S2:** Cloud AX12 video. The video displays the nebulization of 300 μL liquid in the Cloud AX12. Several well openings are taped shut to ensure that the aerosol does not reach these wells.

## Data Availability

The data that support the findings of this study are available on request from the corresponding author. The data are not publicly available due to privacy or ethical restrictions.
