# Supplementary material for: A stretchable human lung‐on‐chip model of alveolar inflammation for evaluating anti‐inflammatory drug response
Source: Bioeng Transl Med. 2024 Sep 5;10(1):e10715. doi: 10.1002/btm2.10715 (PMC11711225; doi:10.1002/btm2.10715)
Supplement: Supplementary file 1 — Data S1: Differential expression analysis report [file BTM2-10-e10715-s001.docx]

A stretchable human lung-on-chip model of alveolar inflammation for evaluating anti-inflammatory drug response – Supplementary Material

Clémentine Richter^ab^, Lorenz Latta^a^, Daria Harig^ab^, Patrick Carius^ab^, Janick D. Stucki^cd^, Nina Hobi^cd^, Andreas Hugi^c^, Paul Schumacher^e^, Tobias Krebs^e^, Alexander Gamrekeli^f^, Felix Stöckle^f^, Klaus Urbschat^g^, Galia Montalvo^hj^, Franziska Lautenschläger^hi^, Brigitta Loretz^a^, Alberto Hidalgo^a^, Nicole Schneider-Daum^a*^, Claus-Michael Lehr^ab^

1. Helmholtz Institute for Pharmaceutical Research Saarland, 66123 Saarbrücken, Germany
2. Department of Pharmacy, Saarland University, 66123 Saarbrücken, Germany
3. AlveoliX AG, Swiss Organs-on-Chip Innovation, 3010 Bern, Switzerland
4. ARTORG Center for Biomedical Engineering Research, Organs-on-Chip Technologies, University of Bern, 3008 Bern, Switzerland
5. Vitrocell® Systems GmbH, 79183 Waldkirch, Germany
6. Center for Thorax Medicine, Clinic Saarbrücken, 66119 Saarbrücken, Germany
7. Section of Thoracic Surgery of the Saar Lung Center, SHG Clinics, 66333 Völklingen, Germany
8. Department of Experimental Physics, Saarland University, 66123 Saarbrücken, Germany
9. Center for Biophysics, Saarland University, 66123 Saarbrücken, Germany
10. Biophysics, Center for Integrative Physiology and Molecular Medicine (CIPMM), School of Medicine, Saarland University, 66421 Homburg, Germany

*corresponding author

# Conflict of Interest Declaration

Some authors of this study are employed by the companies and institutions disclosed on the title page of this manuscript. AH, JS, and NH are employed by AlveoliX AG. JS and NH are minor shareholders of AlveoliX AG. PS is an employee and TK is an employee and a shareholder of Vitrocell® Systems GmbH. All other authors declare that they have no conflicts of interest.

# Data availability statement

The data that support the findings of this study are available on request from Nicole Schneider-Daum (nicole.schneider-daum@helmholtz-hips.de). The RNA-Sequencing dataset is available upon request at the research data archive RADAR (doi.org/10.22000/nfYyspONBoAAnRGx and doi.org/10.22000/xSdEBCHTPOQlmpBi). These data are not publicly available due to privacy or ethical restrictions.

# Funding statement

This project was funded by the Eureka Eurostars program under the number E!12977 - AIM4DoC (Advanced Inhalation Model for Drug Discovery on Chip) and internal funding label 01QE1912C.

# Supplementary figures and videos


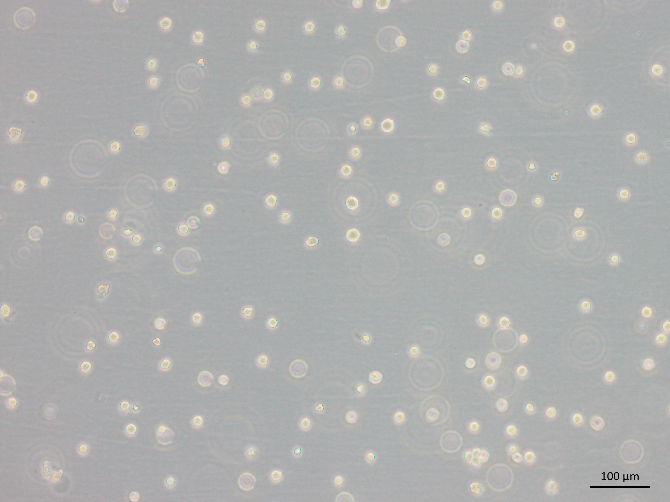

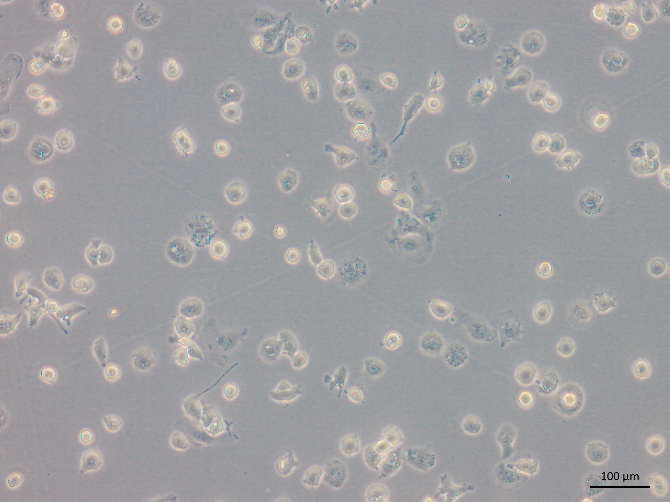


**Suppl. Fig. 1: THP-1 and differentiated THP-1 cells displaying distinct morphological differences**

The THP-1 cell line^1^ was differentiated to a macrophage-like state using 7.5 ng/mL PMA (25 fg/cell) for 2-3 days and left to recover after detaching and seeding.^2-4^ This protocol ensures that the positive control would be differentiated into macrophages (as shown by morphologic changes and attachment to the plastic surface). At the same time, no cytokine release should be detectable in the absence of additional inflammatory stimuli (Suppl. Fig. 2A).^5-7^

Images were taken with an Axio Vert.A1 light microscope (Zeiss) equipped with an AxioCam Erc 5s camera (Zeiss) and analyzed with ZEN Imaging Software (Version ZEN 3.0, blue edition, Zeiss).

**B**

**A**

**Suppl. Fig. 2: Setup of LPS inflammation and BUD treatment parameters on differentiated THP-1 cells**

Setting up a new *in vitro* model requires the characterization of a complex network of parameters, all influencing each other. The concentration of LPS and treatment time point of BUD were first investigated in macrophage mono-cultures to set up the inflammation and treatment protocols and to investigate the *in vitro* relevance of the immune cell component of the deep lung to LPS.

In this step, the concentration of the inflammatory stimulus (LPS), the time point of the anti-inflammatory treatment (BUD), the cell type (mono-cultures of dTHP-1), and the read-outs (release of IL-6 and TNFα) were investigated and defined. The release of cytokines IL-6 and TNFα was measured in the supernatant 24h after inflammation with LPS, similar to time points *in vivo* in mouse and human LPS-induced lung injury.^8,9^

**A** LPS dose finding in macrophage mono-culture via cytokine IL-6 and TNFα release in 96-well plates. LPS assay concentrations of 0.05 µg/mL, 0.5 µg/mL, and 5.0 µg/mL are compared to medium controls. The release of cytokines IL-6 and TNFα was measured in the supernatant 24h after inflammation with LPS. Both cytokines are released in a dose-dependent manner. For the following experiments, an LPS concentration of 0.5 µg/mL was applied. Using a higher concentration of LPS did not further enhance cytokine release but increased the variability of the results. Based on the seeding density of macrophages, 0.5 µg/mL LPS corresponds to 1.75 pg/macrophage, which is comparable to LPS doses in human LPS-induced inflammation setups *in vivo*, which are also in the single-digit pg-range per alveolar macrophage.^9-14^ (n = 11-15 out of 5 independent experiments; One-way ANOVA with subsequent Tukey‘s multiple comparison)

**B** Budesonide treatment time point finding in macrophage mono-culture via cytokine IL-6 and TNFα release in 96-well plates. 1 µM Budesonide was added 2h before inflammation, 2h after inflammation, or 6h after inflammation with 0.5 µg/mL LPS. Prophylactic pre-treatment completely prevents cytokine release, both therapeutic post-treatments significantly reduce cytokine release for IL-6 and TNFα. While prophylactic pre-treatment yields the best results in cytokine release prevention, a therapeutic post-treatment was considered more relevant and realistic. All following experiments were performed with 1 µM BUD 2h after inflammation. (n = 14-15 out of 6 independent experiments; One-way ANOVA with subsequent Tukey‘s multiple comparison)

BUD is applied with maximum doses in humans of 800 µg twice daily.^15,16^ Although the lung fraction is highly dependent on the patient’s inhalation technique, approx. 20% of inhaled BUD should reach the lung without the use of a spacer.^17^ That would correspond to approx. 50 fg/macrophage or 0.3 ng/cm² *in vivo*. The BUD dose per cm² *in vitro* on chip has been chosen to be approx. 1000 times higher (350 ng/cm²) than the maximum daily *in vivo* dose to ensure that a strong drug effect should be measurable *in vitro*. This corresponds to an assay concentration of 1 µM. For future investigations, it would be interesting to see the dose-response curves of different doses of anti-inflammatory drugs on these presented models here.


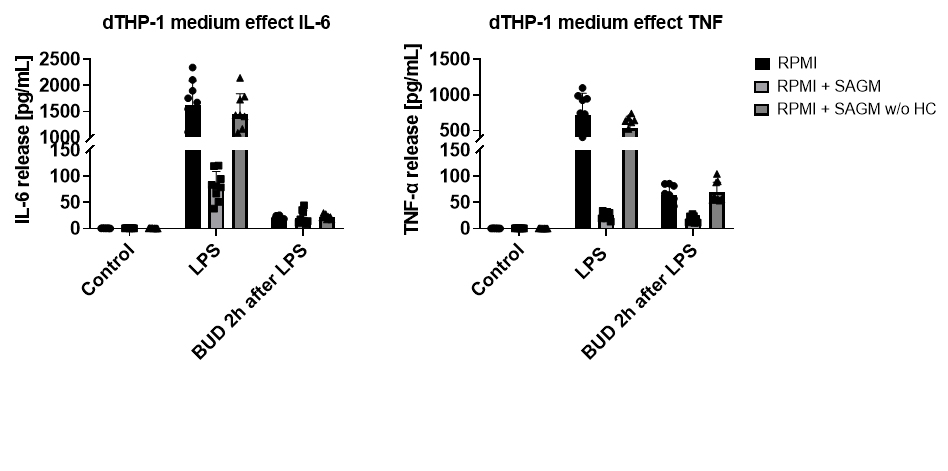


**Suppl. Fig. 3: IL-6 and TNFα release in dTHP-1 mono-cultures cultured with different cell culture media to uncover the effect of hydrocortisone (HC) in the standard co-culture medium on macrophages**

Investigating the variables influencing cytokine release demonstrated a difference in released cytokines from macrophages in mono- or co-cultures (Suppl. Fig. 2B vs. Fig. 2). According to the literature, this may be due to macrophage-epithelial cross-talk to maintain homeostasis,^18^ or the presence of a mixed cell culture medium in the co-cultures, which may bind LPS differently.^19^ These results indicate the presence or absence of HC (chemically identical to the human steroid hormone cortisol) in the co-culture medium as the most critical factor. Hydrocortisone has long been identified as essential for the development of a tight epithelial barrier^20-22^ and is therefore added to the medium for epithelial cells, including the mixed medium for co-cultures, in physiological concentrations (0.5 µg/mL measured via UV spectrometry in the SAGM Single Quots, data not shown; the exact concentration is not disclosed by the supplier).

Legend explanation:

RPMI = standard medium for dTHP-1 (contains no HC)

SAGM = standard medium for Arlo (contains HC)

RPMI + SAGM = standard medium for co-cultures (1:1 v/v, also contains HC)

(n = 9 out of 3 independent experiments)


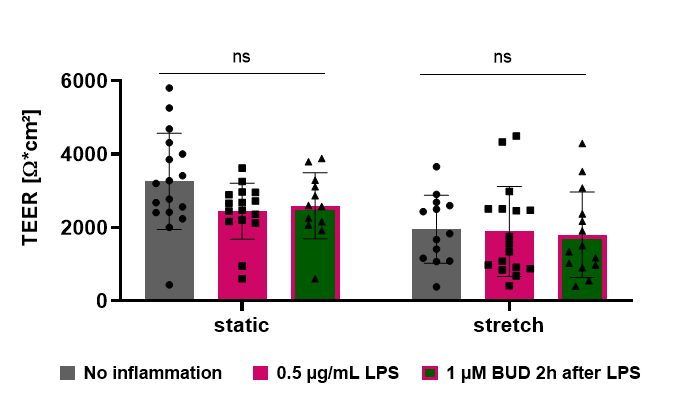


**Suppl. Fig. 4: TEER values of LPS-inflamed co-cultures on chip in LCC**

The lack of TLR4 and its associated proteins CD14 and MD-2 for LPS recognition leads to no effect on the barrier properties of alveolar epithelial cells.^23,24^ Even in the co-culture or with added stretch, there is no measurable effect of LPS on TEER. Stronger inflammatory stimuli are needed to model a critical loss of lung barrier stability. (n = 14-17 out of 7 independent experiments)


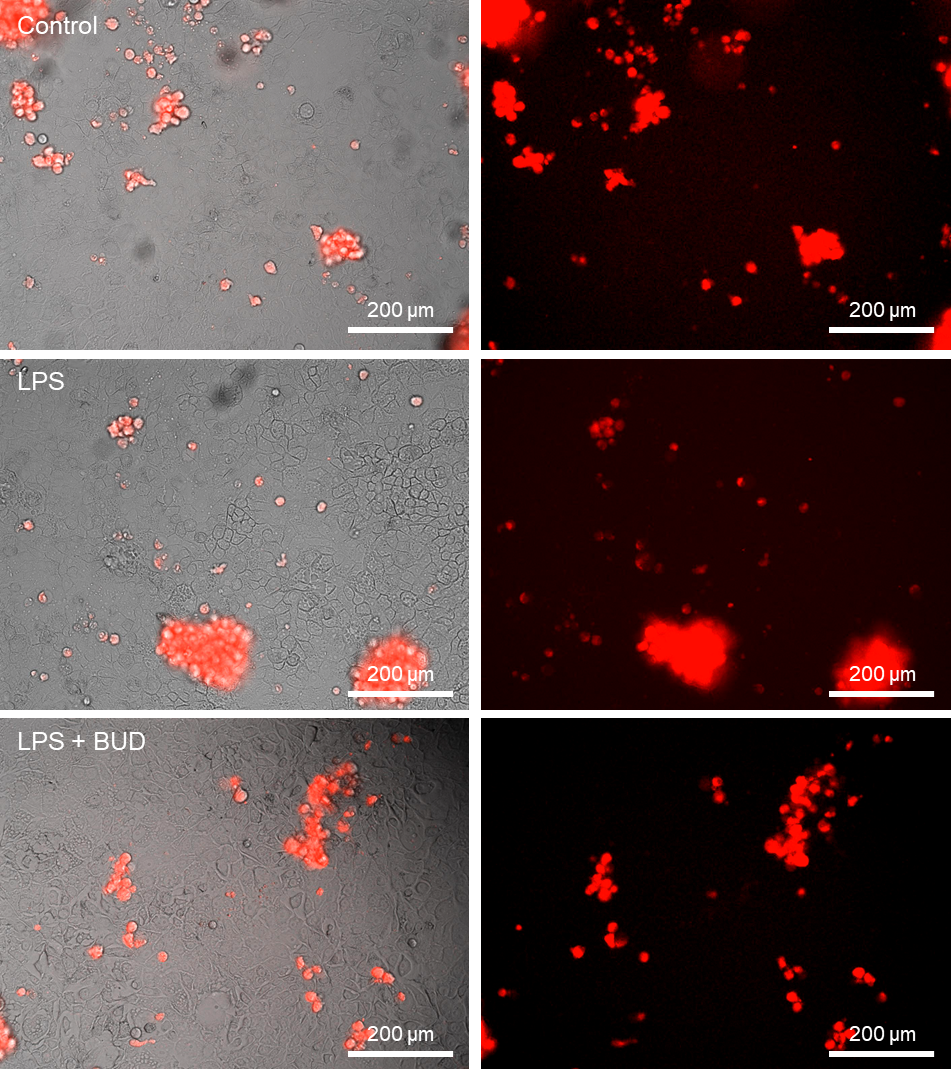


**Suppl. Video 1: Aggregation and mobility of dTHP-1 macrophages in co-culture with Arlo**

These images are placeholders. Please refer to the additional data attached to this publication to view the videos.

Arlo was grown to confluence on Fluorodishes (FD35-100, World Precision Instruments). THP-1 cells were differentiated and stained with CellTrace Far Red (C34564, Invitrogen) according to supplier protocol. After 24h of acclimatization, co-cultures were inflamed, treated, and analyzed under the microscope.

Brigth-field and fluorescent images were recorded using an EMCCD camera (Andor Technology, Belfast, Northern Ireland, UK) with a physical pixel size of 0.65 μm, mounted on a Nikon Eclipse Ti epifluorescent microscope, at a 10X magnification and 0.45 numerical aperture (Nikon Plan Apo objective) over 20 h with a frame rate of 2 min (1 s video corresponds to 20 min in real-time). The cells were kept at a constant atmosphere of 37 °C and 5% CO_2_ (Okolab, Pozzuoli NA, Italy) during the entire experiment. Representative videos out of 6 replicates.


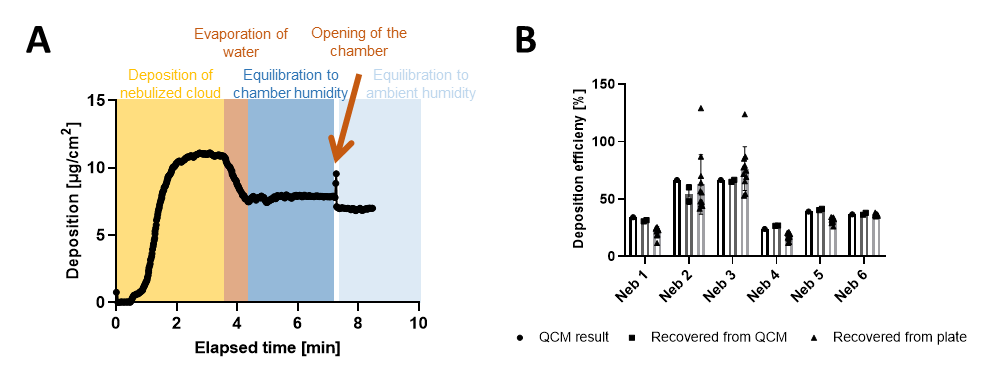


**Suppl. Fig. 5: Deposition efficiency of the Cloud AX12**

**A** Time course of aerosol deposition as monitored by the quartz crystal microbalance (QCM). The initial increase is due to the deposition of aqueous layers, and the decrease after approx. 3 min is due to the evaporation of residual water. Once the value has stabilized, the nebulization chamber is removed (spike in the value) and the final deposition is measured after approx. one minute of equilibration. The weight of PBS salts must be subtracted to calculate the amount of deposited drug (here fluorescein-Na). The final deposition is read after opening the chamber and equilibration of the QCM value to ambient humidity.

**B** Validation of QCM Cloud AX12 deposition efficiency using aerosolized fluorescein-Na. Values from the QCM are compared to the fluorescence signal after recovery of fluorescein from the plate and QCM respectively, indicating acceptable efficacy and reproducibility for six independent nebulizations. This allows for the calculation and use of comparable doses of LPS and BUD as in the previous experiments in LCC by estimating that 30% of the nebulized dose is deposited on the cells. (QCM result: n = 1; recovered from QCM: n = 2; recovered from plate: n = 11-12; out of 6 independent experiments (nebulizations)).


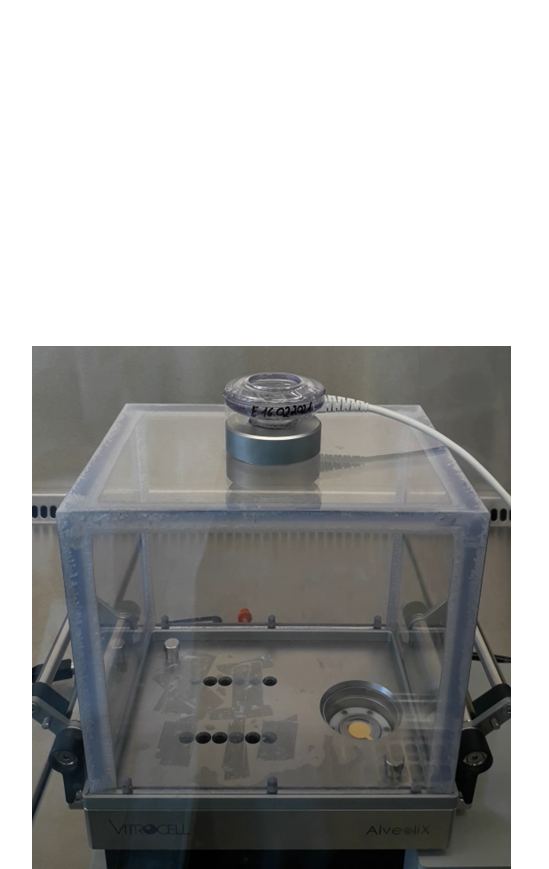


**Suppl. Video 2: Cloud AX12 video**

This image is a placeholder. Please refer to the additional data attached to this publication to view the video.

The video displays the nebulization of 300 µL liquid in the Cloud AX12. Several well openings are taped shut to ensure that the aerosol does not reach these wells.

**Suppl. Fig. 6: TEER development after challenge with 10% DMSO**

The TEER values of the wells used in the experiments for cell stress and cell death measurements after initiating stretch display normal TEER development in static and stretch conditions. Wells challenged with DMSO as negative control displayed no TEER development, confirming the absence of healthy epithelial cells. (n = 4-24 out of 3-4 independent experiments)

# Differential Expression Analysis Report


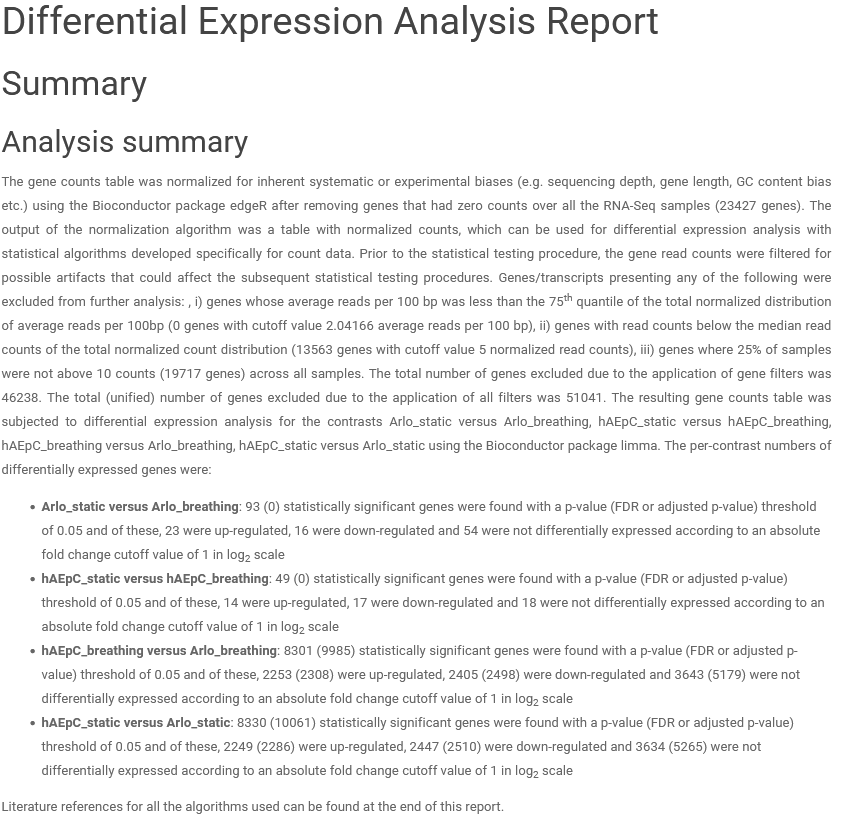


This image is a screenshot of the first page of the analysis report and a placeholder. Please refer to the additional data attached to this publication to view the complete analysis and report.

# Extended Materials and Methods

**Cell culture methods**

**Arlo cell line**

The cell line “Arlo”, a monoclonal lentivirus-immortalized epithelial cell line of the deep lung, was developed in-house and cultured according to a recent publication.^25^

In short, cells were cultivated in a T25 cm^2^ cell culture flask with 7 mL (8 mL over the weekend) SAGM^TM^ (small airway growth medium bullet kit, Lonza, CC-3118)) supplemented with 100 U/mL penicillin, 100 µg/mL streptomycin (GibcoTM Thermo Fisher Scientific Inc., 15140122), 1% FCS (fetal calf serum, Life Technologies, Cat. No. 10270106). The medium was changed every 2-3 days and cells passaged once a week. Before passaging, cells were washed twice with 7 mL PBS buffer (Dulbecco’s PBS, Sigma-Aldrich, D8537) and detached with 2 mL Trypsin-EDTA (0.05%, GibcoTM Thermo Fisher Scientific Inc., 25300-054) for 6 to 8 min. 5 mL PBS supplemented with 1% FCS was used to stop the trypsin reaction. After centrifugation at 300xg for 4 min, the cell pellet was resuspended in 5 mL SAGM medium, cells counted, and 0.7 x 10^6^ cells seeded 7 mL SAGM medium in a freshly coated T25 cm^2^ cell culture flask. Surface coating for Arlo was always performed as described below. For experiments on AX12, Arlo was seeded with a density of 4 x 10^5^ cells/cm² in 70 µL.

**Surface coating**

All surfaces were coated before Arlo cell seeding according to the following protocol, adapted from a previous publication:^26^ collagen type 1 (bovine, 3 mg/mL, Sigma-Aldrich, C4243) and fibronectin (human, 1 mg/mL in distilled and sterile filtered water; Corning^TM^ 356008) were diluted in 1% v/v in distilled and sterile filtered water to constitute the coating solution. The following volumes were used for coating at 37 °C for 2h resp. overnight in the case of AX12: 2 mL in T25 cm^2^ and 30 µL in AX12. Before cell seeding, the coating solution was aspirated, and the surface was left to dry under laminar airflow.

**THP-1 cell line**

The THP-1 cell line^1^ (No. ACC-16, DSMZ (Deutsche Sammlung für Mikroorganismen und Zellkulturen)) was cultivated and differentiated according to a previously published protocol with minor changes.^2^

In short, THP-1 cells were cultivated in T75 cm^2^ cell culture flasks with RPMI medium (Gibco^TM^ Thermo Fisher Scientific Inc., Cat. No. 21875-034) supplemented with 10% FCS (Life Technologies, Cat. No. 10270106) and passaged twice weekly upon reaching a concentration of 1 x 10^6^ cells/mL.

To differentiate THP-1 cells in alveolar macrophage surrogates (dTHP-1), 3 x 10^6^ THP-1 cells were differentiated with 7.5 ng/mL of PMA (phorbol 12-myristate 13-acetate, Sigma-Aldrich, PA585) in 10 mL RPMI medium supplemented with 10% FCS for two to three days.^3^ dTHP-1 cells were washed twice with PBS (Phosphate-buffered saline, Sigma-Aldrich D8537), incubated with 3 mL Accutase (Sigma-Aldrich A6964) for 30 min at 37 °C, and gently detached with a cell scraper (Greiner bio-one 541070). After centrifugation at 300xg for 4 min, the cell pellet was resuspended in 1.5 mL RPMI medium, and cells were counted and seeded in mono- or co-cultures according to the following protocol. Mono-cultures were set up with the same number of cells in the same medium volume as the co-culture they were compared to.

**Co-cultures of Arlo and dTHP-1 cell lines**

Co-cultures of Arlo and dTHP-1 cells were set up according to a previous publication.^2^ In short, upon reaching a tight epithelial barrier with Arlo cells (confirmed via TEER measurement, approx. 500 Ω*cm^2^ in breathing conditions, and approx. 1000 Ω*cm^2^ in static conditions), dTHP-1 cells were seeded on top of the epithelial barrier with 2.4 x 10^5^ cells/cm² in 70 µL (LCC) or 3 µL (ALI).^27^ After macrophage cell seeding, the cells were allowed to interact for 24h before starting inflammation experiments (Fig. 1C, Fig. 5, Suppl. Video 1).^4^ After the co-culture set-up, only small volumes of liquid were added or exchanged apically to avoid disturbing the co-culture, except for cytokine sampling at ALI at the end of the experiment. Feeding of co-cultures was performed only from the basal side and co-cultures were not cultured for longer than 72h in total.

**Human alveolar epithelial cells (hAEpC)**

Primary hAEpCs were isolated according to a previously published protocol.^26^ The human tissue was provided by the Clinic Saarbrücken and the SHG Clinics Völklingen. The procedure and use of patient material are in accordance with the Helsinki Declaration of 1975 (revised 2008) and were permitted by the local ethics committee of the state of Saarland, Germany (May 21, 2019 reference number 113/19 and May 10, 2021 reference number 97/21). All patient materials were delivered with anonymized labels, ensuring patient privacy. The local ethics committee of the state of Saarland, Germany has reviewed the patient consent forms as well.^26^ The cells were seeded and cultivated on chip with 3.5 x 10^5^ cells/cm².

**^AX^Lung-On-Chip System**

**AX12**

The ^AX^Lung-On-Chip system (AlveoliX AG) has been previously described in detail (Fig. 1B).^28,29^ In brief, the ^AX^Lung-On-Chip System consists of the AX12 containing a porous ultrathin membrane, connected to the electro-pneumatic control units (^AX^Exchanger and ^AX^Breather) through the ^AX^Dock. The ^AX^Exchanger is used for medium exchange, sampling of basolateral samples, and TEER measurements. The ^AX^Breather is applying cyclic 3-dimensional stretch. All experiments were conducted according to manufacturer specifications. AX12 were kept at 37°C, 95% humidity, and 5% CO_2_, only to be taken out for cell care and experimental procedures.

**ALI and nebulizing**

Arlo cultures were switched to ALI after the formation of a tight epithelial barrier approx. on day 12 after seeding, when a tight barrier was formed, confirmed via TEER measurement. Nebulization of LPS and BUD was performed using the Cloud AX12^30,31^ (Vitrocell® Systems GmbH) with an Aeroneb® Lab Nebulizer^32^ (standard VMAD, 4.0–6.0 µm droplet diameter) connected to an Aerogen® USB controller according to supplier instructions. LPS was nebulized at a concentration of 907 µg/mL, and BUD at 33.5 µg/mL, both with 300 µL. The volume is given by the manufacturer's instructions, and the concentrations are calculated according to the measured deposition efficiency (Suppl. Fig. 5B).

**Inflammation and treatment protocols**

**Inflammation with LPS**

LPS from *E. coli O26:B6* (Sigma-Aldrich L2762-5MG) stock solution was prepared with 1 µg/mL in PBS, aliquoted, and frozen at -20 °C until use. An LPS response curve (0.05, 0.5, and 5.0 µg/mL) was performed in mono-cultures of dTHP-1 cells to select the optimal concentration for further experiments (Suppl. Fig. 2).^5-7^ The rest of the experiments in mono- and co-cultures were performed with 0.5 µg/mL LPS when in LCC (liquid-covered conditions). In the case of ALI cultures on chip, LPS was nebulized using the Cloud AX12, a system for air-liquid interface exposure^30^ adapted for the lung-on-chip with an Aeroneb® Lab Nebulizer (Aerogen®, standard VMAD, 4.0–6.0 µm droplet diameter) connected to an Aerogen® USB controller (Aerogen®) according to manufacturer’s instructions. In short, the Cloud AX12 System was cleaned and sterilized by wiping with 70% EtOH and set up under laminar flow. The nebulizer was primed by nebulizing 200 µL PBS until output was steady and reproducible. AX12 were placed in the Cloud AX12, 300 µL of LPS solution in PBS were nebulized and left to settle for 5 min.

LPS was deposited with 20 µg/cm^2^, corresponding to 0.5 µg/mL in LCC or 1,75 pg/macrophage, as calculated according to the measured deposition efficiency (Suppl. Fig. 5B).^9-14^ The deposition was monitored using a QCM (quartz crystal microbalance) according to manufacturer instructions.

**Inflammation with TNFα/IFNγ**

TNFα (Sigma-Aldrich H8916) stock solution was prepared with 1.0 µg/mL in PBS, aliquoted, and frozen at -20 °C until use. IFNγ (Miltenyi Biotec 130-096-48) stock solution was prepared with 100 µg/mL in PBS, aliquoted, and frozen at -20 °C. Inflammation with a combination of TNFα/ IFNγ^20^ was performed in LCC with assay concentrations of ~0.1 µg/mL each, corresponding to 96 ng/cm² or 0.33 fg/macrophage for TNFα and 113 ng/cm² or 0.39 fg/macrophage for INFγ.

**Treatment with BUD**

BUD (Sigma-Aldrich, Pharmaceutical Secondary Standard PHR1178) stock was suspended in 100% ethanol at 3 mg/mL. Cultures were treated with an assay concentration of 1 µM BUD apically in LCC.^15-17^ In the case of ALI cultures on chip, BUD was nebulized with the Cloud AX12, according to the same protocol as LPS nebulization, with a deposition of 350 ng/cm² or 1.5 pg/macrophage. To ensure reproducibility and avoid cross-contamination, an output control was performed regularly and the nebulizer was thoroughly cleaned according to manufacturer’s specifications.

**Read-outs**

**Cytokine quantification**

Released cytokines were measured via bead-based FACS (fluorescence-activated cell sorting) assay using Human Soluble Protein Flex Sets for IL-6 (558276), TNFα (560112), and IL-8 (558277) with the Human Soluble Protein Master Buffer Kit (558264, all BD Biosciences). All samples were taken 24h after LPS inflammation.^8,9^ An aliquot of 60 µL apical medium was centrifuged for 4 min at 300xg and 55 µL supernatant was immediately frozen at -80°C. In the case of ALI cultures, LCC were re-established 30 min before the end of the 24h and sampled the same way as LCC cultures. All samples were thawed only once directly before performing the quantification.

The beads were sorted and analyzed with a BD LSRFortessaTM FACS (BD Biosciences). The data was analyzed with FCAP Array Version 3.0.1 for Windows (BD Biosciences).

**Epithelial barrier measurement methods**

TEER on AX12 was measured with an EVOM2 with an adapted range (World Precision Instruments 300523) and electrodes for 96-well plates (World Precision Instruments STX100M). Raw resistance data was corrected for cell growth area with the following formula:


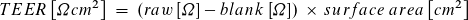


The value for the blank in the AX12 is 450 Ω, the porous surface area is 0.071 cm^2^

The p_app_ (apparent permeability) of the small molecule FluNa (Fluorescein sodium salt, Sigma-Aldrich F6377) was determined according to a previous protocol with minor changes:^33^ After initial TEER measurement, the medium was exchanged for HBSS (Gibco Thermo Fisher Scientific Inc. 14025-050) for one hour before adding FluNa, because the SAGM medium is fluorescent in the green spectrum. After this hour, HBSS containing 10 µg/mL FluNa apically with or without 8 mM EDTA (ethylenediamine tetraacetic acid disodium salt dihydrate, Carl ROTH® 8043.1). 70 µL basal HBSS was sampled every hour, and the missing volume was replaced with fresh HBSS for 7h. The amount of FluNa in the samples was measured via fluorescence with Tecan Infinite 200Pro Photometer (Tecan, λ_ex_ = 485 nm; λ_em_ = 530 nm), and p_app_ [cm/s] was calculated according to the following equation:


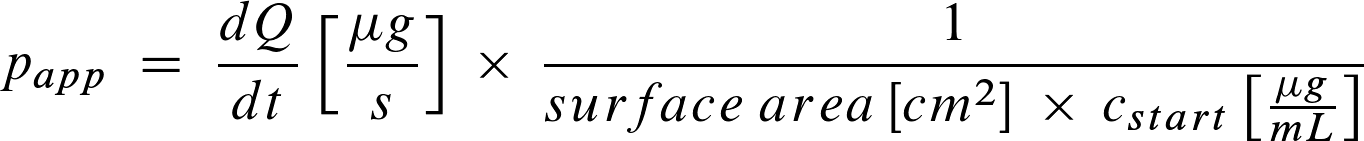


**Cell stress and cell death measurement**

Cell stress and death on chip after the start of the application of stretch were measured with the RealTime-Glo™ Annexin V Apoptosis and Necrosis Assay (Promega JA1011), adapted to smaller volumes to match the AX12. In short, 3 µL of each component of the assay kit (Annexin NanoBiT, Necrosis Detection Reagent, Annexin V-SmBiT, and Annexin) was mixed with 900 µL medium. An aliquot of 30 µL of this mixture was added to the 70 µL medium in the apical compartment, for a total apical volume of 100 µL. Dead control cells were challenged with 10% DMSO (dimethyl sulfoxide, Sigma-Aldrich D2438-5X10ML).

Luminescence (1000 ms) and fluorescence (λ_ex_ = 485 nm; λ_em_ = 525 nm) were measured with a Tecan Spark Cyto 600 cell imager and plate reader (Tecan). Fluorescence was measured from the top without plate cover. The excitation wavelength was 485 nm, and the emission wavelength was 525 nm, each of them with 15.0 nm bandwidth. The gain was manually set to 30%, and the optimal z-position was automatically calculated for each experiment and found to be fluctuating by less than 3%. Luminescence was measured from the top without plate cover for 1000 ms. Blanks were measured and subtracted from all values.

**Confocal microscopy**

Macrophages were stained before seeding with Far Red Cell Tracer (Invitrogen C34564) according to the manufacturer’s specifications. In short, macrophages were stained with 1mL staining solution (1:1000 in PBS) per 1 million cells in suspension in a shaking water bath at 37 °C for 20 min protected from light. The reaction was stopped with five times the reaction volume of RPMI medium with 10% FCS for 5 min. Afterward, the stained cells were centrifuged, washed, counted, and seeded on top of the epithelial cells. Co-cultures with stained macrophages were not used for experiments with other read-outs than confocal microscopy, such as barrier measurement or cytokine release measurement.

Fixation and staining of epithelial cells were performed according to a previous publication with modifications to adapt it to macrophage-epithelial co-cultures:^34^ cultures were washed three times with PBS very gently to minimize loss of macrophages, fixated with 4% PFA (paraformaldehyde, Sigma-Aldrich 30525-89-4) for 15 min at RT (room temperature), washed again very gently three times with PBS and kept under PBS at 4 °C until staining.

Before staining, cultures were permeabilized and blocked with permeabilization buffer (0.05% Saponin (Sigma-Aldrich 43036) and 1% BSA (bovine serum albumin, Sigma-Aldrich A9647) in PBS) for 1h. Occludin was detected with primary antibody (Invitrogen 33-1500, dil. 1:400) overnight at 4 °C and secondary antibody (Invitrogen A21050, dil. 1:2000) for 1h at RT. Actin was stained with Phalloidin with Alexa 488 (Invitrogen A12379, dil. 1:1000) for 30 min at RT. Cell nuclei were stained with DAPI (4′, 6-Diamidino-2-phenylindole dihydrochloride, Sigma-Aldrich 32670, dil. 1:1000) for 30 min at RT. All staining solutions were diluted in the permeabilization buffer. Cells were washed three times for 10 min with PBS while gently shaking between each staining. After staining, AX12 were disassembled according to manufacturer specification and mounted with DAKO Fluorescence Mounting Medium (Agilent 85 S302380-2) on coverslips and left to dry overnight at room temperature.

Confocal images were taken with a confocal laser scanning microscope (Leica, Dmi8 Confocal Laser Scanning Microscope) with a 25x water immersion objective. Lasers at 461 nm (DAPI), 518 nm (Phalloidin), and 650 nm (Occludin, Cell Tracer) were used for detection. Images were analyzed with Imaris Version 9.7.2 for Windows (Oxford Instruments).

**RNA Sequencing and Analysis**

6-7 days after seeding, TEER was measured to ensure that a tight epithelial barrier was formed on chip. Directly after TEER measurement, cells were washed twice with PBS, and RNA was harvested by incubation in lysis buffer (RLT Buffer, Qiagen 79216) from the basal and apical side for 5 min. Two wells were pooled for one sample, and a total of 6 samples out of three independent passages for Arlo or patients for hAEpC were collected. Isolation of RNA was performed with the RNeasy Micro Kit (Qiagen 74004) and the RNase-Free DNase Set (Qiagen 79254) according to the manufacturer’s instructions. Samples with guanidin salt contaminations were additionally cleaned with the Monarch® RNA Cleanup Kit (New England Biolabs T2030L) to achieve the minimum requirements for sequencing (500 ng total RNA, RQN > 8; 260/280 ratio > 1.8 and 230/260 ration < 1.8 respectively).

Sequencing was performed by strand-specific mRNA sequencing. mRNA library was prepared with the NEB Next Ultra II Directional RNA Library Prep Kit (New England Biolabs E7765). Sequencing was performed on NovaSeq 6000, PE50 (2x 50bp) with 30 mio NGS reads per sample and 800 mio cluster flow cell output.

Fastq data was analyzed with a preset pipeline using RNAdetector^35^ running in a docker container. Star alignment with feature counts for read summarizing was chosen as the alignment algorithm.^36^ The gene counts table was normalized for inherent systematic or experimental biases using the Bioconductor package edgeR. A complete analysis summary with a run log can be found in the supplementary data (Suppl. Data 1). Fastq files are deposited at the RADAR portal and can be downloaded upon request (doi.org/10.22000/nfYyspONBoAAnRGx and doi.org/10.22000/xSdEBCHTPOQlmpBi).

**Statistics**

Numerical data are presented as mean ± standard deviation. Graphs were created with GraphPad Prism Version 9.5.0 for Windows. To compare the statistical significance of the results, one-way ANOVA with subsequent Tukey’s multiple comparisons was used. The statistical thresholds for p values were set as follows: 0.12 (ns), 0.033 (*), 0.002 (**), <0.001 (***), according to NEJM (The New England Journal of Medicine) policies.^37^

All experiments were performed at least three independent times, with the exact number of replicates specified in the corresponding figure legends. For all experiments on chip, the control and test wells were randomly chosen to avoid bias caused by slight differences in cell growth between the inner and the outer wells.

References

1. Chanput W, Peters V, Wichers H. The Impact of Food Bioactives on Health: in vitro and ex vivo models: THP-1 and U937 Cells. 2015. doi:10.1007/978-3-319-16104-4_14

2. Kletting S, Barthold S, Repnik U, et al. Co-culture of human alveolar epithelial (hAELVi) and macrophage (THP-1) cell lines. *ALTEX*. 2018;35(2):211-222. doi:10.14573/altex.1607191

3. Chanput W, Mes JJ, Wichers HJ. THP-1 cell line: an in vitro cell model for immune modulation approach. *Int Immunopharmacol*. 2014;23(1):37-45. doi:10.1016/j.intimp.2014.08.002

4. Giambelluca S, Ochs M, Lopez-Rodriguez E. Resting time after phorbol 12-myristate 13-acetate in THP-1 derived macrophages provides a non-biased model for the study of NLRP3 inflammasome. *Front Immunol*. 2022;13:958098. doi:10.3389/fimmu.2022.958098

5. Bisig C, Voss C, Petri-Fink A, Rothen-Rutishauser B. The crux of positive controls - Pro-inflammatory responses in lung cell models. *Toxicol In Vitro*. 2019;54:189-193. doi:10.1016/j.tiv.2018.09.021

6. Park EK, Jung HS, Yang HI, Yoo MC, Kim C, Kim KS. Optimized THP-1 differentiation is required for the detection of responses to weak stimuli. *Inflamm Res*. 2007;56(1):45-50. doi:10.1007/s00011-007-6115-5

7. Takashiba S, van Dyke TE, Amar S, Murayama Y, Soskolne AW, Shapira L. Differentiation of monocytes to macrophages primes cells for lipopolysaccharide stimulation via accumulation of cytoplasmic nuclear factor kappaB. *Infect Immun*. 1999;67(11):5573-5578. doi:10.1128/iai.67.11.5573-5578.1999

8. Matute-Bello G, Frevert CW, Martin TR. Animal models of acute lung injury. *Am J Physiol Lung Cell Mol Physiol*. 2008;295(3):L379-99. doi:10.1152/ajplung.00010.2008

9. Brooks D, Barr LC, Wiscombe S, McAuley DF, Simpson AJ, Rostron AJ. Human lipopolysaccharide models provide mechanistic and therapeutic insights into systemic and pulmonary inflammation. *Eur Respir J*. 2020;56(1). doi:10.1183/13993003.01298-2019

10. Sandström T, Bjermer L, Rylander R. Lipopolysaccharide (LPS) inhalation in healthy subjects increases neutrophils, lymphocytes and fibronectin levels in bronchoalveolar lavage fluid. *Eur Respir J*. 1992;5(8):992-996.

11. Michel O, Nagy AM, Schroeven M, et al. Dose-response relationship to inhaled endotoxin in normal subjects. *Am J Respir Crit Care Med*. 1997;156(4 Pt 1):1157-1164. doi:10.1164/ajrccm.156.4.97-02002

12. Thorn J, Rylander R. Inflammatory response after inhalation of bacterial endotoxin assessed by the induced sputum technique. *Thorax*. 1998;53(12):1047-1052. doi:10.1136/thx.53.12.1047

13. O'Grady NP, Preas HL, Pugin J, et al. Local inflammatory responses following bronchial endotoxin instillation in humans. *Am J Respir Crit Care Med*. 2001;163(7):1591-1598. doi:10.1164/ajrccm.163.7.2009111

14. Zielen S, Trischler J, Schubert R. Lipopolysaccharide challenge: immunological effects and safety in humans. *Expert Rev Clin Immunol*. 2015;11(3):409-418. doi:10.1586/1744666X.2015.1012158

15. Brogden RN, McTavish D. Budesonide. An updated review of its pharmacological properties, and therapeutic efficacy in asthma and rhinitis. *Drugs*. 1992;44(3):375-407. doi:10.2165/00003495-199244030-00007

16. Davis KC, Small RE. Budesonide inhalation powder: a review of its pharmacologic properties and role in the treatment of asthma. *Pharmacotherapy*. 1998;18(4):720-728.

17. Hirst PH, Bacon RE, Pitcairn GR, SILVASTI M, Newman. A comparison of the lung deposition of budesonide from Easyhaler®, Turbuhaler®and pMDI plus spacer in asthmatic patients // A comparison of the lung deposition of budesonide from Easyhaler, Turbuhaler and pMDI plus spacer in asthmatic patients. *Respiratory Medicine*. 2001;95(9):720-727. doi:10.1053/rmed.2001.1107

18. Bissonnette EY, Lauzon-Joset J-F, Debley JS, Ziegler SF. Cross-Talk Between Alveolar Macrophages and Lung Epithelial Cells is Essential to Maintain Lung Homeostasis. *Front Immunol*. 2020;11:583042. doi:10.3389/fimmu.2020.583042

19. Stromberg LR, Mendez HM, Kubicek-Sutherland JZ, Graves SW, Hengartner NW, Mukundan H. Presentation matters: Impact of association of amphiphilic LPS with serum carrier proteins on innate immune signaling. *PLoS One*. 2018;13(6):e0198531. doi:10.1371/journal.pone.0198531

20. Metz JK, Wiegand B, Schnur S, et al. Modulating the Barrier Function of Human Alveolar Epithelial (hAELVi) Cell Monolayers as a Model of Inflammation. *Altern Lab Anim*. 2020;48(5-6):252-267. doi:10.1177/0261192920983015

21. Kürti L, Veszelka S, Bocsik A, et al. Retinoic acid and hydrocortisone strengthen the barrier function of human RPMI 2650 cells, a model for nasal epithelial permeability. *Cytotechnology*. 2013;65(3):395-406. doi:10.1007/s10616-012-9493-7

22. Zhaeentan S, Amjadi FS, Zandie Z, Joghataei MT, Bakhtiyari M, Aflatoonian R. The effects of hydrocortisone on tight junction genes in an in vitro model of the human fallopian epithelial cells. *Eur J Obstet Gynecol Reprod Biol*. 2018;229:127-131. doi:10.1016/j.ejogrb.2018.05.034

23. Sikkema L, Ramírez-Suástegui C, Strobl DC, et al. An integrated cell atlas of the lung in health and disease. *Nat Med*. 2023;29(6):1563-1577. doi:10.1038/s41591-023-02327-2

24. Mills-Goodlet R, Schenck M, Chary A, et al. Biological effects of allergen–nanoparticle conjugates: uptake and immune effects determined on hAELVi cells under submerged vs. air–liquid interface conditions. *Environ Sci.: Nano*. 2020;7(7):2073-2086. doi:10.1039/C9EN01353A

25. Carius P, Jungmann A, Bechtel M, et al. A Monoclonal Human Alveolar Epithelial Cell Line ("Arlo") with Pronounced Barrier Function for Studying Drug Permeability and Viral Infections. *Adv Sci (Weinh)*. 2023:e2207301. doi:10.1002/advs.202207301

26. Daum N, Kuehn A, Hein S, Schaefer UF, Huwer H, Lehr C-M. Isolation, cultivation, and application of human alveolar epithelial cells. *Methods Mol Biol*. 2012;806:31-42. doi:10.1007/978-1-61779-367-7_3

27. Stone KC, Mercer RR, Gehr P, Stockstill B, Crapo JD. Allometric relationships of cell numbers and size in the mammalian lung. *Am J Respir Cell Mol Biol*. 1992;6(2):235-243. doi:10.1165/ajrcmb/6.2.235

28. Stucki JD, Hobi N, Galimov A, et al. Medium throughput breathing human primary cell alveolus-on-chip model. *Sci Rep*. 2018;8(1):14359. doi:10.1038/s41598-018-32523-x

29. Sengupta A, Roldan N, Kiener M, et al. A New Immortalized Human Alveolar Epithelial Cell Model to Study Lung Injury and Toxicity on a Breathing Lung-On-Chip System. *Front Toxicol*. 2022;4:840606. doi:10.3389/ftox.2022.840606

30. Lenz AG, Karg E, Lentner B, et al. A dose-controlled system for air-liquid interface cell exposure and application to zinc oxide nanoparticles. *Part Fibre Toxicol*. 2009;6:32. doi:10.1186/1743-8977-6-32

31. Sengupta A, Dorn A, Jamshidi M, et al. A multiplex inhalation platform to model in situ like aerosol delivery in a breathing lung-on-chip. *Front Pharmacol*. 2023;14:1114739. doi:10.3389/fphar.2023.1114739

32. Byron PR. Drug delivery devices: issues in drug development. *Proc Am Thorac Soc*. 2004;1(4):321-328. doi:10.1513/pats.200403-023MS

33. Kuehn A, Kletting S, Souza Carvalho-Wodarz C de, et al. Human alveolar epithelial cells expressing tight junctions to model the air-blood barrier. *ALTEX*. 2016;33(3):251-260. doi:10.14573/altex.1511131

34. Montefusco-Pereira CV, Horstmann JC, Ebensen T, et al. P. aeruginosa Infected 3D Co-Culture of Bronchial Epithelial Cells and Macrophages at Air-Liquid Interface for Preclinical Evaluation of Anti-Infectives. *J Vis Exp*. 2020;(160). doi:10.3791/61069

35. La Ferlita A, Alaimo S, Di Bella S, et al. RNAdetector: a free user-friendly stand-alone and cloud-based system for RNA-Seq data analysis. *BMC Bioinformatics*. 2021;22(1):298. doi:10.1186/s12859-021-04211-7

36. Dobin A, Davis CA, Schlesinger F, et al. STAR: ultrafast universal RNA-seq aligner. *Bioinformatics*. 2013;29(1):15-21. doi:10.1093/bioinformatics/bts635

37. The New England Journal of Medicine. New Manuscripts: Statistical Reporting Guidelines. Accessed August 2, 2023. https://www.nejm.org/author-center/new-manuscripts
