# Supplementary material for: A stretchable human lung‐on‐chip model of alveolar inflammation for evaluating anti‐inflammatory drug response
Source: Bioeng Transl Med. 2024 Sep 5;10(1):e10715. doi: 10.1002/btm2.10715 (PMC11711225; doi:10.1002/btm2.10715)
Supplement: Supplementary file 2 — Data S2: Supporting Information [file BTM2-10-e10715-s002.zip › index.html]

Differential Expression Analysis Report


# Differential Expression Analysis Report

# Summary

## Analysis summary

The gene counts table was normalized for inherent systematic or experimental biases (e.g. sequencing depth, gene length, GC content bias etc.) using the Bioconductor package edgeR after removing genes that had zero counts over all the RNA-Seq samples (23427 genes). The output of the normalization algorithm was a table with normalized counts, which can be used for differential expression analysis with statistical algorithms developed specifically for count data. Prior to the statistical testing procedure, the gene read counts were filtered for possible artifacts that could affect the subsequent statistical testing procedures. Genes/transcripts presenting any of the following were excluded from further analysis: , i) genes whose average reads per 100 bp was less than the 75th quantile of the total normalized distribution of average reads per 100bp (0 genes with cutoff value 2.04166 average reads per 100 bp), ii) genes with read counts below the median read counts of the total normalized count distribution (13563 genes with cutoff value 5 normalized read counts), iii) genes where 25% of samples were not above 10 counts (19717 genes) across all samples. The total number of genes excluded due to the application of gene filters was 46238. The total (unified) number of genes excluded due to the application of all filters was 51041. The resulting gene counts table was subjected to differential expression analysis for the contrasts Arlo\_static versus Arlo\_breathing, hAEpC\_static versus hAEpC\_breathing, hAEpC\_breathing versus Arlo\_breathing, hAEpC\_static versus Arlo\_static using the Bioconductor package limma. The per-contrast numbers of differentially expressed genes were:

- **Arlo\_static versus Arlo\_breathing**: 93 (0) statistically significant genes were found with a p-value (FDR or adjusted p-value) threshold of 0.05 and of these, 23 were up-regulated, 16 were down-regulated and 54 were not differentially expressed according to an absolute fold change cutoff value of 1 in log2 scale
- **hAEpC\_static versus hAEpC\_breathing**: 49 (0) statistically significant genes were found with a p-value (FDR or adjusted p-value) threshold of 0.05 and of these, 14 were up-regulated, 17 were down-regulated and 18 were not differentially expressed according to an absolute fold change cutoff value of 1 in log2 scale
- **hAEpC\_breathing versus Arlo\_breathing**: 8301 (9985) statistically significant genes were found with a p-value (FDR or adjusted p-value) threshold of 0.05 and of these, 2253 (2308) were up-regulated, 2405 (2498) were down-regulated and 3643 (5179) were not differentially expressed according to an absolute fold change cutoff value of 1 in log2 scale
- **hAEpC\_static versus Arlo\_static**: 8330 (10061) statistically significant genes were found with a p-value (FDR or adjusted p-value) threshold of 0.05 and of these, 2249 (2286) were up-regulated, 2447 (2510) were down-regulated and 3634 (5265) were not differentially expressed according to an absolute fold change cutoff value of 1 in log2 scale

Literature references for all the algorithms used can be found at the end of this report.

## Input options

**Read counts file:** imported custom data frame  
 **Conditions:** Arlo\_breathing, Arlo\_static, hAEpC\_breathing, hAEpC\_static  
 **Samples included:** X008\_Arlo\_br\_N1, X010\_Arlo\_br\_N2, X012\_Arlo\_br\_N3, X007\_Arlo\_st\_N1, X009\_Arlo\_st\_N2, X011\_Arlo\_st\_N3, X002\_hAEpC\_br\_N1, X004\_hAEpC\_br\_N2, X006\_hAEpC\_br\_N3, X001\_hAEpC\_st\_N1, X003\_hAEpC\_st\_N2, X005\_hAEpC\_st\_N3  
 **Samples excluded:** none  
 **Requested contrasts:** Arlo\_static\_vs\_Arlo\_breathing, hAEpC\_static\_vs\_hAEpC\_breathing, hAEpC\_breathing\_vs\_Arlo\_breathing, hAEpC\_static\_vs\_Arlo\_static  
 **Library sizes:** not available  
 **Annotation:** embedded  
 **Organism:**   
 **Annotation source:** Ensembl genomes  
 **Count type:** gene

**Gene filters:**

- *avg.reads*
  - average.per.bp: 100
  - quantile: 0.75
- *expression*
  - median: 1
  - mean: 0
- *presence*
  - frac: 0.25
  - min.count: 10
  - per.condition: FALSE
  - min\_count: 10
  - per\_condition: 1

**Filter application:** before normalization  
 **Normalization algorithm:** edgeR  
 **Normalization arguments:** method, refColumn, logratioTrim, sumTrim, doWeighting, Acutoff, p   

- method: TMM
- logratioTrim: 0.3
- sumTrim: 0.05
- doWeighting: TRUE
- Acutoff: -1e+10
- p: 0.75

**Statistical algorithm(s):**  limma  
 **Statistical arguments for limma:**

- normalize.method: none

**Meta-analysis method:** Simes correction and combination method  
 **Multiple testing correction:**  Storey-Tibshirani FDR  
 **p-value threshold:**  0.05  
 **Logarithmic tranformation offset:**  1  
 **Analysis preset:**  not available  
 **Quality control plots:** multidimensional scaling, correlation heatmap and correlogram, pairwise scatterplots between samples, boxplots, DEG heatmap, volcano plot  
 **Figure format:**  png, pdf  
 **Output directory:**  /rnadetector/ws/storage/app/public/jobs/176/deg\_report\_all-in-one-comparsion  
 **Output data:**  Annotation, p-value, Adjusted p-value (FDR), Fold change, Statistics  
 **Output scale(s):**  Natural scale, log2 scale  
 **Output values:**  Raw values, Normalized values  
 **Output statistics:** Mean, Median, Standard deviation  
 **Total run time:** 04 minutes 11 seconds

## Filtered genes

**Number of filtered genes:** 51041 *which is the* ***union*** *of*

- Filtered because of zero reads: 23427
- Filtered because of exon filters: 0
- Filtered because of gene filters: 27614 *which is the* ***union*** *of*
  - avg.reads: 26520 genes with filter cutoff value 2.041664
  - expression: 13563 genes further decomposed to (filter name, filtered genes, filter cutoff):
    - median: 13563 genes with filter cutoff value 5
  - presence: 19717 genes with filter cutoff value 3

## Differentially expressed genes

**Number of differentially expressed genes per contrast:**

- **Arlo\_static\_vs\_Arlo\_breathing:** 93 (0) statistically significant genes of which 23 up regulated, 16 down regulated and 54 not differentially expressed according to a p-value (FDR or adjusted p-value) threshold of 0.05 and an absolute fold change cutoff value of 1 in log2 scale.
- **hAEpC\_static\_vs\_hAEpC\_breathing:** 49 (0) statistically significant genes of which 14 up regulated, 17 down regulated and 18 not differentially expressed according to a p-value (FDR or adjusted p-value) threshold of 0.05 and an absolute fold change cutoff value of 1 in log2 scale.
- **hAEpC\_breathing\_vs\_Arlo\_breathing:** 8301 (9985) statistically significant genes of which 2253 (2308) up regulated, 2405 (2498) down regulated and 3643 (5179) not differentially expressed according to a p-value (FDR or adjusted p-value) threshold of 0.05 and an absolute fold change cutoff value of 1 in log2 scale.
- **hAEpC\_static\_vs\_Arlo\_static:** 8330 (10061) statistically significant genes of which 2249 (2286) up regulated, 2447 (2510) down regulated and 3634 (5265) not differentially expressed according to a p-value (FDR or adjusted p-value) threshold of 0.05 and an absolute fold change cutoff value of 1 in log2 scale.

## Run log

```
WARN  [2023-08-29 15:35:03] The column that contains the gene biotypes ("bt.col") is missing with embedded annotation! Biotype filters and certain plots will not be available...  
WARN  [2023-08-29 15:35:03] The creation of a Venn diagram is possible only when more than one statistical algorithms are used (meta-analysis)! Removing from figures list...  
WARN  [2023-08-29 15:35:03] The following edger normalization argument names are invalid and will be ignored: locfunc  
INFO  [2023-08-29 15:35:03] 2023-08-29 15:35:03: Data processing started...  
INFO  [2023-08-29 15:35:03] Read counts file: imported custom data frame  
INFO  [2023-08-29 15:35:03] Conditions: Arlo_breathing, Arlo_static, hAEpC_breathing, hAEpC_static  
INFO  [2023-08-29 15:35:03] Samples to include: X008_Arlo_br_N1, X010_Arlo_br_N2, X012_Arlo_br_N3, X007_Arlo_st_N1, X009_Arlo_st_N2, X011_Arlo_st_N3, X002_hAEpC_br_N1, X004_hAEpC_br_N2, X006_hAEpC_br_N3, X001_hAEpC_st_N1, X003_hAEpC_st_N2, X005_hAEpC_st_N3  
INFO  [2023-08-29 15:35:03] Samples to exclude: none  
INFO  [2023-08-29 15:35:03] Requested contrasts: Arlo_static_vs_Arlo_breathing, hAEpC_static_vs_hAEpC_breathing, hAEpC_breathing_vs_Arlo_breathing, hAEpC_static_vs_Arlo_static  
INFO  [2023-08-29 15:35:03] Annotation: embedded  
INFO  [2023-08-29 15:35:03] Organism: custom  
INFO  [2023-08-29 15:35:03] Reference source: ensembl  
INFO  [2023-08-29 15:35:03] Count type: gene  
INFO  [2023-08-29 15:35:03] Transcriptional level: gene  
INFO  [2023-08-29 15:35:03] Exon filters: min.active.exons  
INFO  [2023-08-29 15:35:03]   min.active.exons:   
INFO  [2023-08-29 15:35:03]     exons.per.gene: 5  
INFO  [2023-08-29 15:35:03]     min.exons: 2  
INFO  [2023-08-29 15:35:03]     frac: 0.2  
INFO  [2023-08-29 15:35:03] Gene filters: avg.reads, expression, presence  
INFO  [2023-08-29 15:35:03]   avg.reads:   
INFO  [2023-08-29 15:35:03]     average.per.bp: 100  
INFO  [2023-08-29 15:35:03]     quantile: 0.75  
INFO  [2023-08-29 15:35:03]   expression:   
INFO  [2023-08-29 15:35:03]     median: 1  
INFO  [2023-08-29 15:35:03]     mean: 0  
INFO  [2023-08-29 15:35:03]     custom: NA  
INFO  [2023-08-29 15:35:03]   presence:   
INFO  [2023-08-29 15:35:03]     frac: 0.25  
INFO  [2023-08-29 15:35:03]     min.count: 10  
INFO  [2023-08-29 15:35:03]     per.condition: FALSE  
INFO  [2023-08-29 15:35:03]     min_count: 10  
INFO  [2023-08-29 15:35:03]     per_condition: 1  
INFO  [2023-08-29 15:35:03] Filter application: prenorm  
INFO  [2023-08-29 15:35:03] Normalization algorithm: edger  
INFO  [2023-08-29 15:35:03] Normalization arguments:   
INFO  [2023-08-29 15:35:03]   method: TMM  
INFO  [2023-08-29 15:35:03]   logratioTrim: 0.3  
INFO  [2023-08-29 15:35:03]   sumTrim: 0.05  
INFO  [2023-08-29 15:35:03]   doWeighting: TRUE  
INFO  [2023-08-29 15:35:03]   Acutoff: -1e+10  
INFO  [2023-08-29 15:35:03]   p: 0.75  
INFO  [2023-08-29 15:35:03] Statistical algorithm: limma  
INFO  [2023-08-29 15:35:03] Statistical arguments:   
INFO  [2023-08-29 15:35:03]   limma: none  
INFO  [2023-08-29 15:35:03] Meta-analysis method: simes  
INFO  [2023-08-29 15:35:03] Multiple testing correction: qvalue  
INFO  [2023-08-29 15:35:03] p-value threshold: 0.05  
INFO  [2023-08-29 15:35:03] Logarithmic transformation offset: 1  
INFO  [2023-08-29 15:35:03] Quality control plots: mds, readnoise, correl, pairwise, boxplot, deheatmap, volcano  
INFO  [2023-08-29 15:35:03] Figure format: png, pdf  
INFO  [2023-08-29 15:35:03] Output directory: /rnadetector/ws/storage/app/public/jobs/176/deg_report_all-in-one-comparsion  
INFO  [2023-08-29 15:35:03] Output data: annotation, p.value, adj.p.value, meta.p.value, adj.meta.p.value, fold.change, stats  
INFO  [2023-08-29 15:35:03] Output scale(s): natural, log2  
INFO  [2023-08-29 15:35:03] Output values: raw, normalized  
INFO  [2023-08-29 15:35:03] Output statistics: mean, median, sd  
INFO  [2023-08-29 15:35:03] Saving gene model to /rnadetector/ws/storage/app/public/jobs/176/deg_report_all-in-one-comparsion/data/gene_model.RData  
INFO  [2023-08-29 15:35:03] Removing genes with zero counts in all samples...  
INFO  [2023-08-29 15:35:04] Prefiltering normalization with: edger  
INFO  [2023-08-29 15:35:05] Applying gene filter avg.reads...  
INFO  [2023-08-29 15:35:05]   Threshold below which ignored: 2.04166386697721  
INFO  [2023-08-29 15:35:05] Applying gene filter expression...  
INFO  [2023-08-29 15:35:05]   Threshold below which ignored: 5  
INFO  [2023-08-29 15:35:05] Applying gene filter presence...  
INFO  [2023-08-29 15:35:05]   Threshold below which ignored: 3  
INFO  [2023-08-29 15:35:05] Normalizing with: edger  
INFO  [2023-08-29 15:35:05] 51041 genes filtered out  
INFO  [2023-08-29 15:35:05] 11662 genes remain after filtering  
INFO  [2023-08-29 15:35:05] Running statistical tests with: limma  
INFO  [2023-08-29 15:35:05]   Contrast: Arlo_static_vs_Arlo_breathing  
INFO  [2023-08-29 15:35:06]   Contrast: hAEpC_static_vs_hAEpC_breathing  
INFO  [2023-08-29 15:35:06]   Contrast: hAEpC_breathing_vs_Arlo_breathing  
INFO  [2023-08-29 15:35:07]   Contrast: hAEpC_static_vs_Arlo_static  
INFO  [2023-08-29 15:35:08]   Contrast Arlo_static_vs_Arlo_breathing: found 93 genes  
INFO  [2023-08-29 15:35:08]   Contrast hAEpC_static_vs_hAEpC_breathing: found 49 genes  
INFO  [2023-08-29 15:35:08]   Contrast hAEpC_breathing_vs_Arlo_breathing: found 8301 genes  
INFO  [2023-08-29 15:35:08]   Contrast hAEpC_static_vs_Arlo_static: found 8330 genes  
INFO  [2023-08-29 15:35:08] Exporting and compressing normalized read counts table to /rnadetector/ws/storage/app/public/jobs/176/deg_report_all-in-one-comparsion/lists/normalized_counts_table.txt  
INFO  [2023-08-29 15:35:09] Building output files...  
INFO  [2023-08-29 15:35:09]   Contrast: Arlo_static_vs_Arlo_breathing  
INFO  [2023-08-29 15:35:09]     Adding non-filtered data...  
INFO  [2023-08-29 15:35:09]       binding annotation...  
INFO  [2023-08-29 15:35:09]       binding p-values...  
INFO  [2023-08-29 15:35:09]       binding FDRs...  
INFO  [2023-08-29 15:35:09]       binding natural normalized fold changes...  
INFO  [2023-08-29 15:35:09]       binding log2 normalized fold changes...  
INFO  [2023-08-29 15:35:09]       binding natural raw fold changes...  
INFO  [2023-08-29 15:35:11]       binding log2 raw fold changes...  
INFO  [2023-08-29 15:35:11]       binding normalized mean counts...  
INFO  [2023-08-29 15:35:11]       binding normalized median counts...  
INFO  [2023-08-29 15:35:12]       binding normalized count sds...  
INFO  [2023-08-29 15:35:12]       binding raw mean counts...  
INFO  [2023-08-29 15:35:12]       binding raw median counts...  
INFO  [2023-08-29 15:35:13]       binding raw counts sds...  
INFO  [2023-08-29 15:35:13]       binding normalized mean counts...  
INFO  [2023-08-29 15:35:13]       binding normalized median counts...  
INFO  [2023-08-29 15:35:14]       binding normalized count sds...  
INFO  [2023-08-29 15:35:14]       binding raw mean counts...  
INFO  [2023-08-29 15:35:14]       binding raw median counts...  
INFO  [2023-08-29 15:35:15]       binding raw counts sds...  
INFO  [2023-08-29 15:35:15]     Writing output...  
INFO  [2023-08-29 15:35:15]     Adding filtered data...  
INFO  [2023-08-29 15:35:15]       binding annotation...  
INFO  [2023-08-29 15:35:15]       binding p-values...  
INFO  [2023-08-29 15:35:15]       binding FDRs...  
INFO  [2023-08-29 15:35:16]       binding natural normalized fold changes...  
INFO  [2023-08-29 15:35:16]       binding log2 normalized fold changes...  
INFO  [2023-08-29 15:35:16]       binding natural raw fold changes...  
INFO  [2023-08-29 15:35:16]       binding log2 raw fold changes...  
INFO  [2023-08-29 15:35:16]       binding normalized mean counts...  
INFO  [2023-08-29 15:35:16]       binding normalized median counts...  
INFO  [2023-08-29 15:35:19]       binding normalized count sds...  
INFO  [2023-08-29 15:35:21]       binding raw mean counts...  
INFO  [2023-08-29 15:35:21]       binding raw median counts...  
INFO  [2023-08-29 15:35:24]       binding raw counts sds...  
INFO  [2023-08-29 15:35:27]       binding normalized mean counts...  
INFO  [2023-08-29 15:35:28]       binding normalized median counts...  
INFO  [2023-08-29 15:35:30]       binding normalized count sds...  
INFO  [2023-08-29 15:35:31]       binding raw mean counts...  
INFO  [2023-08-29 15:35:32]       binding raw median counts...  
INFO  [2023-08-29 15:35:34]       binding raw counts sds...  
INFO  [2023-08-29 15:35:35]     Writing output...  
INFO  [2023-08-29 15:35:38]   Contrast: hAEpC_static_vs_hAEpC_breathing  
INFO  [2023-08-29 15:35:38]     Adding non-filtered data...  
INFO  [2023-08-29 15:35:38]       binding annotation...  
INFO  [2023-08-29 15:35:38]       binding p-values...  
INFO  [2023-08-29 15:35:38]       binding FDRs...  
INFO  [2023-08-29 15:35:38]       binding natural normalized fold changes...  
INFO  [2023-08-29 15:35:38]       binding log2 normalized fold changes...  
INFO  [2023-08-29 15:35:38]       binding natural raw fold changes...  
INFO  [2023-08-29 15:35:38]       binding log2 raw fold changes...  
INFO  [2023-08-29 15:35:38]       binding normalized mean counts...  
INFO  [2023-08-29 15:35:38]       binding normalized median counts...  
INFO  [2023-08-29 15:35:39]       binding normalized count sds...  
INFO  [2023-08-29 15:35:39]       binding raw mean counts...  
INFO  [2023-08-29 15:35:39]       binding raw median counts...  
INFO  [2023-08-29 15:35:40]       binding raw counts sds...  
INFO  [2023-08-29 15:35:40]       binding normalized mean counts...  
INFO  [2023-08-29 15:35:40]       binding normalized median counts...  
INFO  [2023-08-29 15:35:41]       binding normalized count sds...  
INFO  [2023-08-29 15:35:41]       binding raw mean counts...  
INFO  [2023-08-29 15:35:41]       binding raw median counts...  
INFO  [2023-08-29 15:35:42]       binding raw counts sds...  
INFO  [2023-08-29 15:35:42]     Writing output...  
INFO  [2023-08-29 15:35:42]     Adding filtered data...  
INFO  [2023-08-29 15:35:42]       binding annotation...  
INFO  [2023-08-29 15:35:42]       binding p-values...  
INFO  [2023-08-29 15:35:42]       binding FDRs...  
INFO  [2023-08-29 15:35:43]       binding natural normalized fold changes...  
INFO  [2023-08-29 15:35:43]       binding log2 normalized fold changes...  
INFO  [2023-08-29 15:35:43]       binding natural raw fold changes...  
INFO  [2023-08-29 15:35:43]       binding log2 raw fold changes...  
INFO  [2023-08-29 15:35:43]       binding normalized mean counts...  
INFO  [2023-08-29 15:35:43]       binding normalized median counts...  
INFO  [2023-08-29 15:35:46]       binding normalized count sds...  
INFO  [2023-08-29 15:35:47]       binding raw mean counts...  
INFO  [2023-08-29 15:35:48]       binding raw median counts...  
INFO  [2023-08-29 15:35:50]       binding raw counts sds...  
INFO  [2023-08-29 15:35:52]       binding normalized mean counts...  
INFO  [2023-08-29 15:35:52]       binding normalized median counts...  
INFO  [2023-08-29 15:35:55]       binding normalized count sds...  
INFO  [2023-08-29 15:35:56]       binding raw mean counts...  
INFO  [2023-08-29 15:35:59]       binding raw median counts...  
INFO  [2023-08-29 15:36:01]       binding raw counts sds...  
INFO  [2023-08-29 15:36:02]     Writing output...  
INFO  [2023-08-29 15:36:05]   Contrast: hAEpC_breathing_vs_Arlo_breathing  
INFO  [2023-08-29 15:36:05]     Adding non-filtered data...  
INFO  [2023-08-29 15:36:05]       binding annotation...  
INFO  [2023-08-29 15:36:05]       binding p-values...  
INFO  [2023-08-29 15:36:05]       binding FDRs...  
INFO  [2023-08-29 15:36:05]       binding natural normalized fold changes...  
INFO  [2023-08-29 15:36:05]       binding log2 normalized fold changes...  
INFO  [2023-08-29 15:36:05]       binding natural raw fold changes...  
INFO  [2023-08-29 15:36:05]       binding log2 raw fold changes...  
INFO  [2023-08-29 15:36:05]       binding normalized mean counts...  
INFO  [2023-08-29 15:36:05]       binding normalized median counts...  
INFO  [2023-08-29 15:36:05]       binding normalized count sds...  
INFO  [2023-08-29 15:36:06]       binding raw mean counts...  
INFO  [2023-08-29 15:36:06]       binding raw median counts...  
INFO  [2023-08-29 15:36:06]       binding raw counts sds...  
INFO  [2023-08-29 15:36:07]       binding normalized mean counts...  
INFO  [2023-08-29 15:36:07]       binding normalized median counts...  
INFO  [2023-08-29 15:36:07]       binding normalized count sds...  
INFO  [2023-08-29 15:36:08]       binding raw mean counts...  
INFO  [2023-08-29 15:36:08]       binding raw median counts...  
INFO  [2023-08-29 15:36:08]       binding raw counts sds...  
INFO  [2023-08-29 15:36:09]     Writing output...  
INFO  [2023-08-29 15:36:09]     Adding filtered data...  
INFO  [2023-08-29 15:36:09]       binding annotation...  
INFO  [2023-08-29 15:36:09]       binding p-values...  
INFO  [2023-08-29 15:36:09]       binding FDRs...  
INFO  [2023-08-29 15:36:10]       binding natural normalized fold changes...  
INFO  [2023-08-29 15:36:10]       binding log2 normalized fold changes...  
INFO  [2023-08-29 15:36:10]       binding natural raw fold changes...  
INFO  [2023-08-29 15:36:10]       binding log2 raw fold changes...  
INFO  [2023-08-29 15:36:10]       binding normalized mean counts...  
INFO  [2023-08-29 15:36:11]       binding normalized median counts...  
INFO  [2023-08-29 15:36:13]       binding normalized count sds...  
INFO  [2023-08-29 15:36:14]       binding raw mean counts...  
INFO  [2023-08-29 15:36:15]       binding raw median counts...  
INFO  [2023-08-29 15:36:18]       binding raw counts sds...  
INFO  [2023-08-29 15:36:19]       binding normalized mean counts...  
INFO  [2023-08-29 15:36:20]       binding normalized median counts...  
INFO  [2023-08-29 15:36:22]       binding normalized count sds...  
INFO  [2023-08-29 15:36:24]       binding raw mean counts...  
INFO  [2023-08-29 15:36:25]       binding raw median counts...  
INFO  [2023-08-29 15:36:27]       binding raw counts sds...  
INFO  [2023-08-29 15:36:29]     Writing output...  
INFO  [2023-08-29 15:36:31]   Contrast: hAEpC_static_vs_Arlo_static  
INFO  [2023-08-29 15:36:31]     Adding non-filtered data...  
INFO  [2023-08-29 15:36:31]       binding annotation...  
INFO  [2023-08-29 15:36:31]       binding p-values...  
INFO  [2023-08-29 15:36:31]       binding FDRs...  
INFO  [2023-08-29 15:36:32]       binding natural normalized fold changes...  
INFO  [2023-08-29 15:36:32]       binding log2 normalized fold changes...  
INFO  [2023-08-29 15:36:32]       binding natural raw fold changes...  
INFO  [2023-08-29 15:36:32]       binding log2 raw fold changes...  
INFO  [2023-08-29 15:36:32]       binding normalized mean counts...  
INFO  [2023-08-29 15:36:32]       binding normalized median counts...  
INFO  [2023-08-29 15:36:33]       binding normalized count sds...  
INFO  [2023-08-29 15:36:33]       binding raw mean counts...  
INFO  [2023-08-29 15:36:33]       binding raw median counts...  
INFO  [2023-08-29 15:36:34]       binding raw counts sds...  
INFO  [2023-08-29 15:36:34]       binding normalized mean counts...  
INFO  [2023-08-29 15:36:34]       binding normalized median counts...  
INFO  [2023-08-29 15:36:35]       binding normalized count sds...  
INFO  [2023-08-29 15:36:36]       binding raw mean counts...  
INFO  [2023-08-29 15:36:36]       binding raw median counts...  
INFO  [2023-08-29 15:36:36]       binding raw counts sds...  
INFO  [2023-08-29 15:36:37]     Writing output...  
INFO  [2023-08-29 15:36:37]     Adding filtered data...  
INFO  [2023-08-29 15:36:37]       binding annotation...  
INFO  [2023-08-29 15:36:37]       binding p-values...  
INFO  [2023-08-29 15:36:37]       binding FDRs...  
INFO  [2023-08-29 15:36:38]       binding natural normalized fold changes...  
INFO  [2023-08-29 15:36:38]       binding log2 normalized fold changes...  
INFO  [2023-08-29 15:36:38]       binding natural raw fold changes...  
INFO  [2023-08-29 15:36:38]       binding log2 raw fold changes...  
INFO  [2023-08-29 15:36:38]       binding normalized mean counts...  
INFO  [2023-08-29 15:36:40]       binding normalized median counts...  
INFO  [2023-08-29 15:36:42]       binding normalized count sds...  
INFO  [2023-08-29 15:36:43]       binding raw mean counts...  
INFO  [2023-08-29 15:36:44]       binding raw median counts...  
INFO  [2023-08-29 15:36:47]       binding raw counts sds...  
INFO  [2023-08-29 15:36:49]       binding normalized mean counts...  
INFO  [2023-08-29 15:36:49]       binding normalized median counts...  
INFO  [2023-08-29 15:36:51]       binding normalized count sds...  
INFO  [2023-08-29 15:36:53]       binding raw mean counts...  
INFO  [2023-08-29 15:36:53]       binding raw median counts...  
INFO  [2023-08-29 15:36:56]       binding raw counts sds...  
INFO  [2023-08-29 15:36:57]     Writing output...  
INFO  [2023-08-29 15:37:00] Creating quality control graphs...  
INFO  [2023-08-29 15:37:00] Plotting in png format...  
INFO  [2023-08-29 15:37:00]   Plotting mds...  
INFO  [2023-08-29 15:37:00]   Plotting readnoise...  
INFO  [2023-08-29 15:37:01]   Plotting correl...  
INFO  [2023-08-29 15:37:01]   Plotting pairwise...  
INFO  [2023-08-29 15:37:38]   Plotting boxplot...  
INFO  [2023-08-29 15:37:38]   Plotting boxplot...  
INFO  [2023-08-29 15:37:38]   Plotting deheatmap...  
INFO  [2023-08-29 15:37:38]   Contrast: Arlo_static_vs_Arlo_breathing  
INFO  [2023-08-29 15:37:39]   Contrast: hAEpC_static_vs_hAEpC_breathing  
INFO  [2023-08-29 15:37:39]   Contrast: hAEpC_breathing_vs_Arlo_breathing  
INFO  [2023-08-29 15:37:39]   Contrast: hAEpC_static_vs_Arlo_static  
INFO  [2023-08-29 15:37:39]   Plotting volcano...  
INFO  [2023-08-29 15:37:39]   Contrast: Arlo_static_vs_Arlo_breathing  
INFO  [2023-08-29 15:37:39]   Contrast: hAEpC_static_vs_hAEpC_breathing  
INFO  [2023-08-29 15:37:40]   Contrast: hAEpC_breathing_vs_Arlo_breathing  
INFO  [2023-08-29 15:37:40]   Contrast: hAEpC_static_vs_Arlo_static  
INFO  [2023-08-29 15:37:40] Plotting in pdf format...  
INFO  [2023-08-29 15:37:40]   Plotting mds...  
INFO  [2023-08-29 15:37:40]   Plotting readnoise...  
INFO  [2023-08-29 15:37:43]   Plotting correl...  
INFO  [2023-08-29 15:37:43]   Plotting pairwise...  
INFO  [2023-08-29 15:39:08]   Plotting boxplot...  
INFO  [2023-08-29 15:39:08]   Plotting boxplot...  
INFO  [2023-08-29 15:39:08]   Plotting deheatmap...  
INFO  [2023-08-29 15:39:08]   Contrast: Arlo_static_vs_Arlo_breathing  
INFO  [2023-08-29 15:39:08]   Contrast: hAEpC_static_vs_hAEpC_breathing  
INFO  [2023-08-29 15:39:08]   Contrast: hAEpC_breathing_vs_Arlo_breathing  
INFO  [2023-08-29 15:39:09]   Contrast: hAEpC_static_vs_Arlo_static  
INFO  [2023-08-29 15:39:09]   Plotting volcano...  
INFO  [2023-08-29 15:39:09]   Contrast: Arlo_static_vs_Arlo_breathing  
INFO  [2023-08-29 15:39:09]   Contrast: hAEpC_static_vs_hAEpC_breathing  
INFO  [2023-08-29 15:39:09]   Contrast: hAEpC_breathing_vs_Arlo_breathing  
INFO  [2023-08-29 15:39:10]   Contrast: hAEpC_static_vs_Arlo_static  
INFO  [2023-08-29 15:39:10] Creating HTML report...  
INFO  [2023-08-29 15:39:10] Compressing figures...
```

# Figures

## Multidimensional scaling

The Multi-Dimensional Scaling (MDS) plots comprise a means of visualizing the level of similarity of individual cases of a dataset. It is similar to Principal Component Analysis (PCA), but instead of using the covariance matrix to find similarities among cases, MDS uses absolute distance metrics such as the classical Euclidean distance. Because of the relative linear relations among sequencing samples, it provides a more realistic clustering among samples. MDS serves quality control and it can be interpreted as follows: when the distance among samples of the same biological condition in the MDS space is small, this is an indication of high correlation and reproducibility among them. When this distance is larger or heterogeneous (e.g. the 3rd sample of a triplicate set is further from the other 2), this constitutes an indication of low correlation and reproducibility among samples. It can help exclude poor samples from further analysis.

## RNA-Seq reads noise

The read noise plots depict the percentage of biological features detected when subsampling the total number of reads. Very steep curves in read noise plots indicate that although the sequencing depth reaches its maximum, a relatively small percentage of total features is detected, indicating that the level of background noise is relatively high. Less steep RNA composition curves, indicate less noise. When a sample’s curve deviate from the rest, it could indicate lower or higher quality, depending on the curves of the rest of the samples.

## Correlation plots

The sample correlation plots depict the accordance among the RNA-Seq samples, as this is manifested through the read counts table used with the metaseqr pipeline, with two representations that both use the correlation matrix (a matrix which depicts all the pairwise correlations between each pair of samples) of the read counts matrix. The first is a correlation clustered heatmap which depicts the correlations among samples as color-scaled image and the hierarchical clustering tree depicts the grouping of the samples according to their correlation. Samples from the same group that are not clustered together provides an indication that there might be a quality problem with the dataset. The second is a ‘correlogram’ plot, where again the samples are hierarchically clustered and grouped but this time correlations are presented as ellipses inside each cell. Each cell represents a pairwise comparison and each correlation coefficient is represented by an ellipse whose ‘diameter’, direction and color depict the accordance for that pair of samples. Highly correlated samples are depicted as ellipses with narrow diameter, while poorly correlated samples are depicted as ellipses with wide diameters. Also, highly correlated samples are depicted as ellipses with a left-to-right upwards direction while poorly correlated samples are depicted as ellipses with a right-to-left upwards direction.

Correlation heatmap

Data correlogram

## Pairwise scatterplots

The pairwise comparison plots are split in three parts: the upper diagonal consists of simple scatterplots for all pairwise sample comparisons, together with their Pearson correlation coefficient. It is a simple measure of between sample correlation using all the available data points instead of only the correlation matrix. The lower diagonal consists of mean-difference plots for all pairwise sample comparisons. A mean-difference plot (or a Bland-Altman plots) is a method of data plotting used in analyzing the agreement between two different assays/variables. In this graphical method the differences (or alternatively the ratios) between the two variables are plotted against the averages of the two. Such a plot is useful, for example, for analyzing data with strong correlation between x and y axes, when the (x,y) dots on the plot are close to the diagonal x=y. In this case, the value of the transformed variable X is about the same as x and y and the variable Y shows the difference between x and y. In both represantations, irregular shapes of the red smoother lines are an indication of poor correlation between samples or of other systematic bias sources, which is usually corrected through data normalization.

## Boxplots

The boxplot comprises a means of summarizing the read counts distribution of a sample in the form of a bar with extending lines, as a commonly used way of graphically presenting groups of numerical data. A boxplot also indicates which observations, if any, might be considered outliers and is able to visually show different types of populations, without making any assumptions about the underlying statistical distribution. The spacings between the different parts of the box help indicate variance, skewness and identify outliers. The thick bar inside the colored box is the median of the observations while the box extends over the Interquartile Range of the observations. The whiskers extend up (down) to +/-1.5xIQR. Boxplots at similar levels indicate good quality of the normalization. If boxplots remain at different levels after normalization, maybe another normalization algorithm may have to be examined. The un-normalized boxplots show the need for data normalization in order for the data from different samples to follow the same underlying distribution and statistical testing becoming possible.

Boxplot of un-normalized data

Boxplot of normalized data

## Volcano plots

A volcano plot is a scatterplot that is often used when analyzing high-throughput -omics data (e.g. microarray data, RNA-Seq data) to give an overview of interesting genes. The log2 fold change is plotted on the x-axis and the negative log10 p-value is plotted on the y-axis. A volcano plot combines the results of a statistical test (aka, p-values) with the magnitude of the change enabling quick visual identification of those genes that display large-magnitude changes that are also statistically significant. The horizontal dashed line sets the threshold for statistical significance, while the vertical dashed lines set the thresholds for biological significance. It should be noted that the volcano plots become harder to interpret when using more than one statistical algorithm and performing meta-analysis. This happens because the genes that have stronger evidence of being differentially expressed obtain lower p-values while the rest either remain at similar levels or obtain higher p-values. The result is a ‘warped’ volcano plot, with two main data clouds: one in the upper part of the plot, and one in the lower part of the plot. You can always zoom in when using interacting mode (the default).

## DEG heatmaps

The Differentially Expressed Genes (DEGs) heatmaps depict how well samples from different conditions cluster together according to their expression values after normalization and statistical testing, for each requested statistical contrast. If samples from the same biological condition do not cluster together, this would constitute a warning sign regarding the quality of the samples. In addition, DEG heatmaps provide an initial view of possible clusters of co-expressed genes.

DEG heatmap for the contrast Arlo\_static vs Arlo\_breathing

DEG heatmap for the contrast hAEpC\_static vs hAEpC\_breathing

DEG heatmap for the contrast hAEpC\_breathing vs Arlo\_breathing

DEG heatmap for the contrast hAEpC\_static vs Arlo\_static

## Download Figures

Figures are available in the following formats:

- PNG
- PDF

# Results

## Arlo\_static vs Arlo\_breathing

The following table presents the top 10% statistically significant genes (use the download links below the table to retrieve the whole list) for the contrast **Arlo\_static vs Arlo\_breathing**. The fields of the table correspond to the requested features to be exported. The table can be searched using the search field on the top right.

**Download the DEG result list for Arlo\_static vs Arlo\_breathing.**

**Download the whole result list for Arlo\_static vs Arlo\_breathing.**

## hAEpC\_static vs hAEpC\_breathing

The following table presents the top 10% statistically significant genes (use the download links below the table to retrieve the whole list) for the contrast **hAEpC\_static vs hAEpC\_breathing**. The fields of the table correspond to the requested features to be exported. The table can be searched using the search field on the top right.

**Download the DEG result list for hAEpC\_static vs hAEpC\_breathing.**

**Download the whole result list for hAEpC\_static vs hAEpC\_breathing.**

## hAEpC\_breathing vs Arlo\_breathing

The following table presents the top 10% statistically significant genes (use the download links below the table to retrieve the whole list) for the contrast **hAEpC\_breathing vs Arlo\_breathing**. The fields of the table correspond to the requested features to be exported. The table can be searched using the search field on the top right.

**Download the DEG result list for hAEpC\_breathing vs Arlo\_breathing.**

**Download the whole result list for hAEpC\_breathing vs Arlo\_breathing.**

## hAEpC\_static vs Arlo\_static

The following table presents the top 10% statistically significant genes (use the download links below the table to retrieve the whole list) for the contrast **hAEpC\_static vs Arlo\_static**. The fields of the table correspond to the requested features to be exported. The table can be searched using the search field on the top right.

**Download the DEG result list for hAEpC\_static vs Arlo\_static.**

**Download the whole result list for hAEpC\_static vs Arlo\_static.**

**Download the normalized read counts table for the experiment.**

# References

1. Robinson, M.D., McCarthy, D.J., and Smyth, G.K. (2010). edgeR: a Bioconductor package for differential expression analysis of digital gene expression data. Bioinformatics 26, 139-140.
2. Smyth, G. (2005). Limma: linear models for microarray data. In Bioinformatics and Computational Biology Solutions using R and Bioconductor, G. R., C. V., D. S., I. R., and H. W., eds. (New York, Springer), pp. 397-420.
3. Planet, E., Attolini, C.S., Reina, O., Flores, O., and Rossell, D. (2012). htSeqTools: high-throughput sequencing quality control, processing and visualization in R. Bioinformatics 28, 589-590.
4. Tarazona, S., Garcia-Alcalde, F., Dopazo, J., Ferrer, A., and Conesa, A. (2011). Differential expression in RNA-seq: a matter of depth. Genome Res 21, 2213-2223.
5. Storey, J.D., and Tibshirani, R. (2003). Statistical significance for genomewide studies. Proc Natl Acad Sci U S A 100, 9440-9445.
6. Simes, R. J. (1986). An improved Bonferroni procedure for multiple tests of significance. Biometrika 73 (3): 751-754.
7. Moulos, P. and Hatzis P. (2014). Systematic integration of RNA-Seq statistical algorithms for accurate detection of differential gene expression patterns. Nucleic Acids Research, 10.1093/nar/gku1273.

---

Copyright © 2013 Talianidis Lab/BSRC Alexander Fleming, Original design by Panagiotis Moulos - Custom design for RNAdetector by Salvatore Alaimo
