# Supplementary figures and images for: A stretchable human lung‐on‐chip model of alveolar inflammation for evaluating anti‐inflammatory drug response
Source: Bioeng Transl Med. 2024 Sep 5;10(1):e10715. doi: 10.1002/btm2.10715 (PMC11711225; doi:10.1002/btm2.10715)

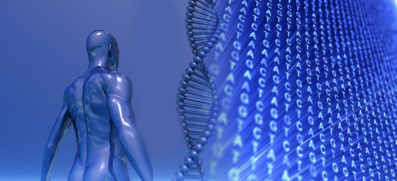

Supplement: Supplementary file 2 — Data S2: Supporting Information [file BTM2-10-e10715-s002.zip › media/logo.png]

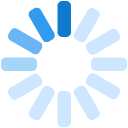

Supplement: Supplementary file 2 — Data S2: Supporting Information [file BTM2-10-e10715-s002.zip › media/loader.gif]

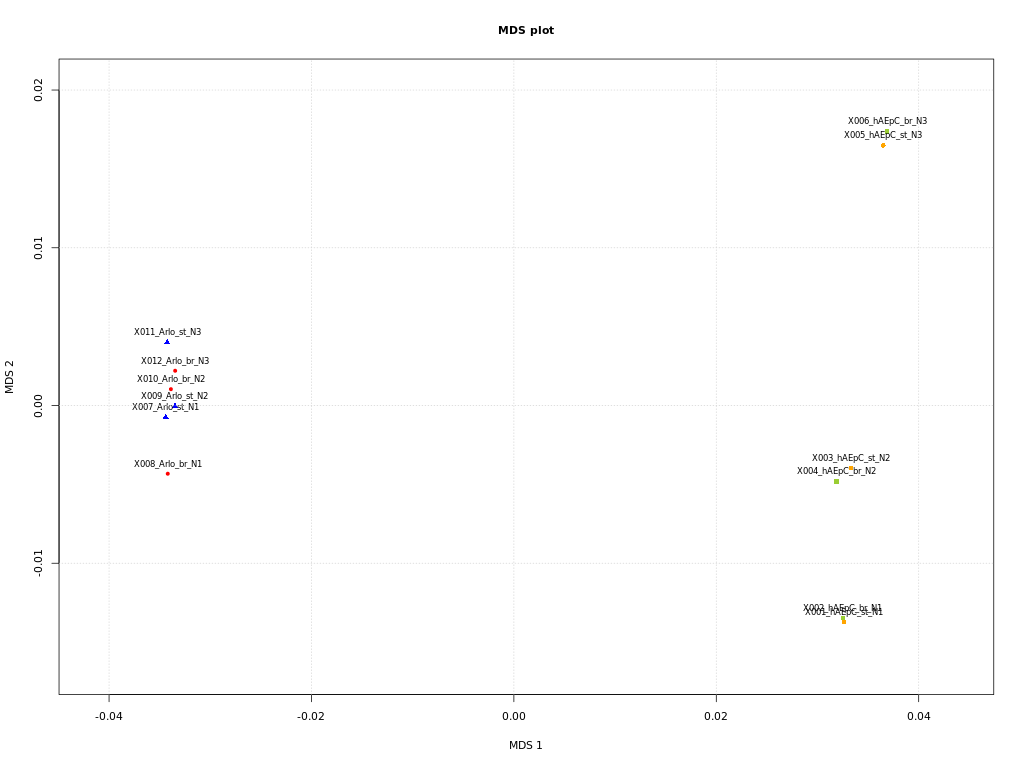

Supplement: Supplementary file 2 — Data S2: Supporting Information [file BTM2-10-e10715-s002.zip › plots/qc/mds.png]

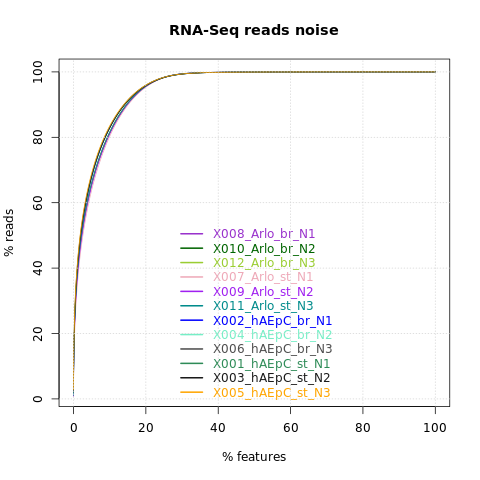

Supplement: Supplementary file 2 — Data S2: Supporting Information [file BTM2-10-e10715-s002.zip › plots/qc/readnoise.png]

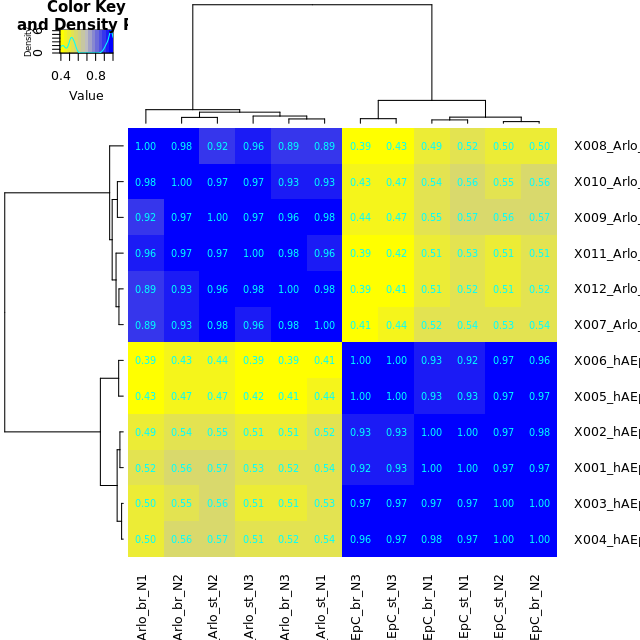

Supplement: Supplementary file 2 — Data S2: Supporting Information [file BTM2-10-e10715-s002.zip › plots/qc/correlation_heatmap.png]

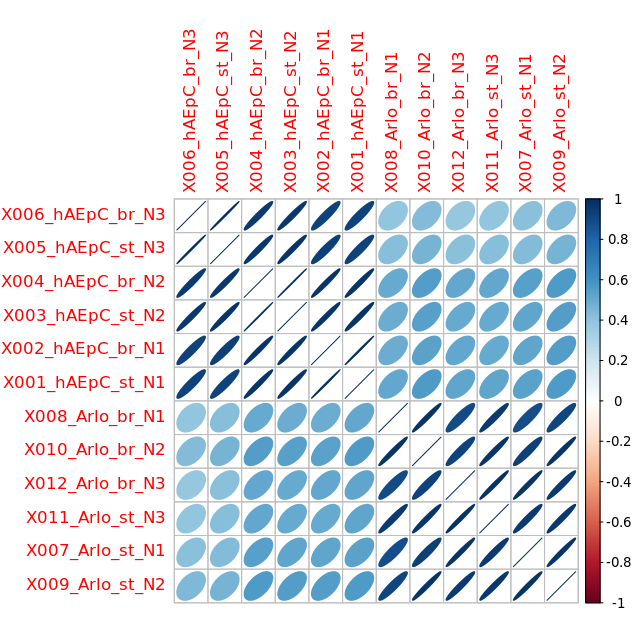

Supplement: Supplementary file 2 — Data S2: Supporting Information [file BTM2-10-e10715-s002.zip › plots/qc/correlation_correlogram.png]

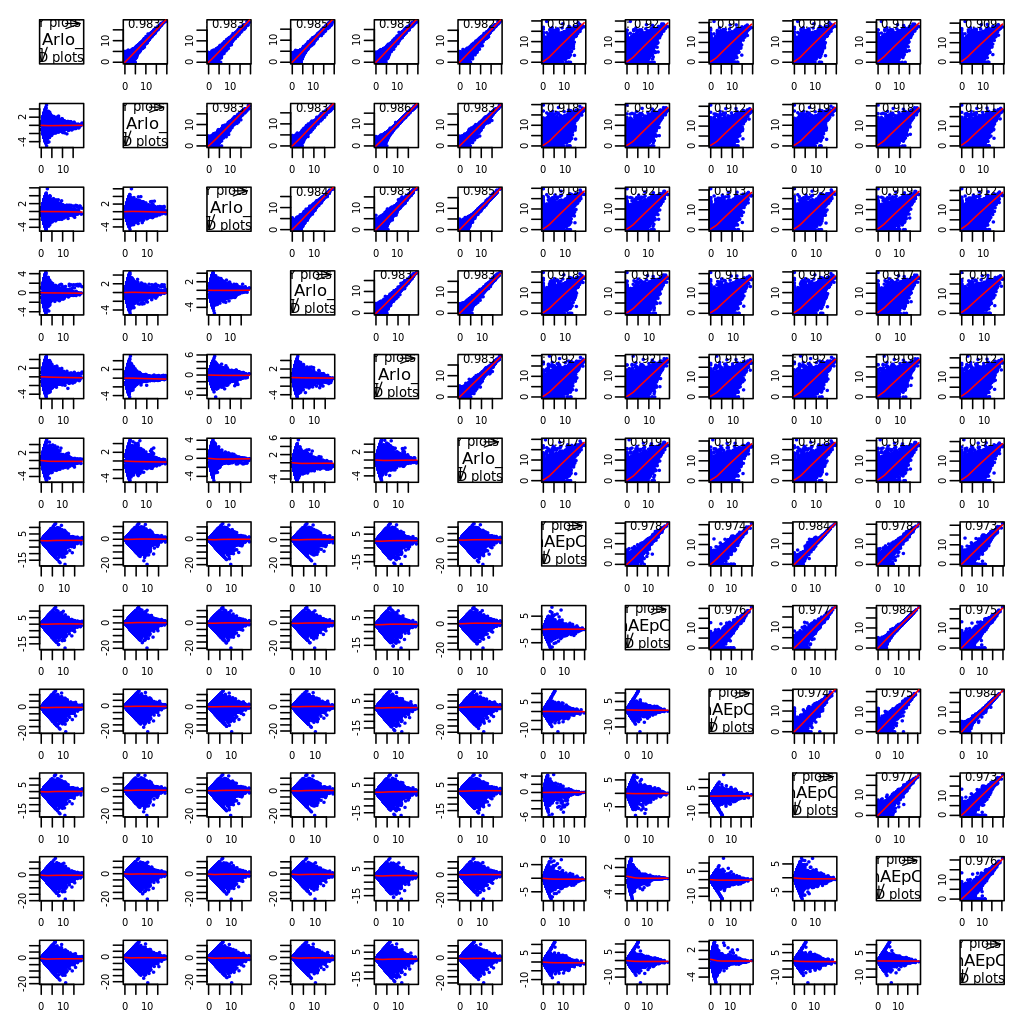

Supplement: Supplementary file 2 — Data S2: Supporting Information [file BTM2-10-e10715-s002.zip › plots/qc/correlation_pairs.png]

MDS plot

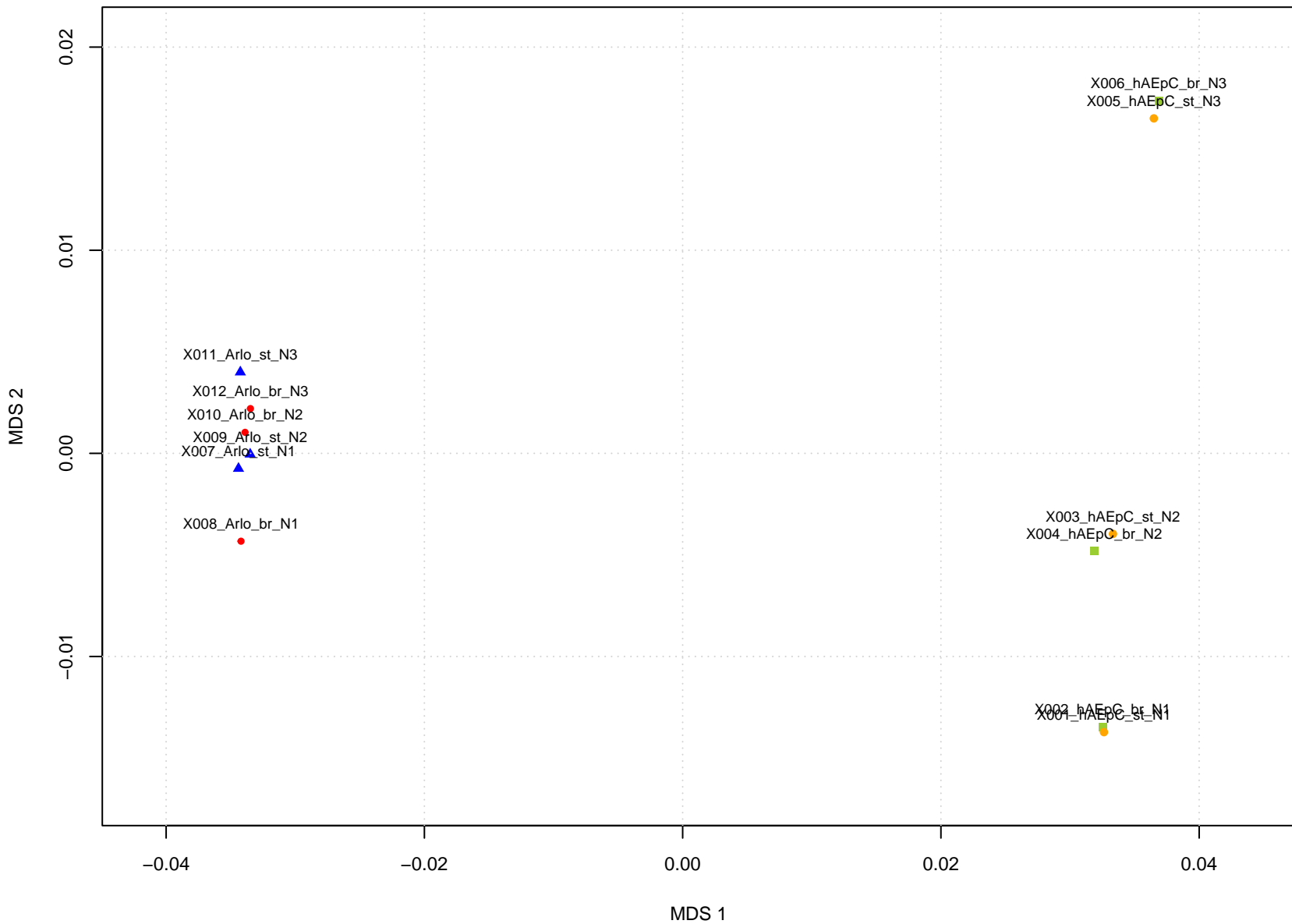

Supplement: Supplementary file 2 — Data S2: Supporting Information [file BTM2-10-e10715-s002.zip › plots/qc/mds.pdf]

## RNA-Seq reads noise

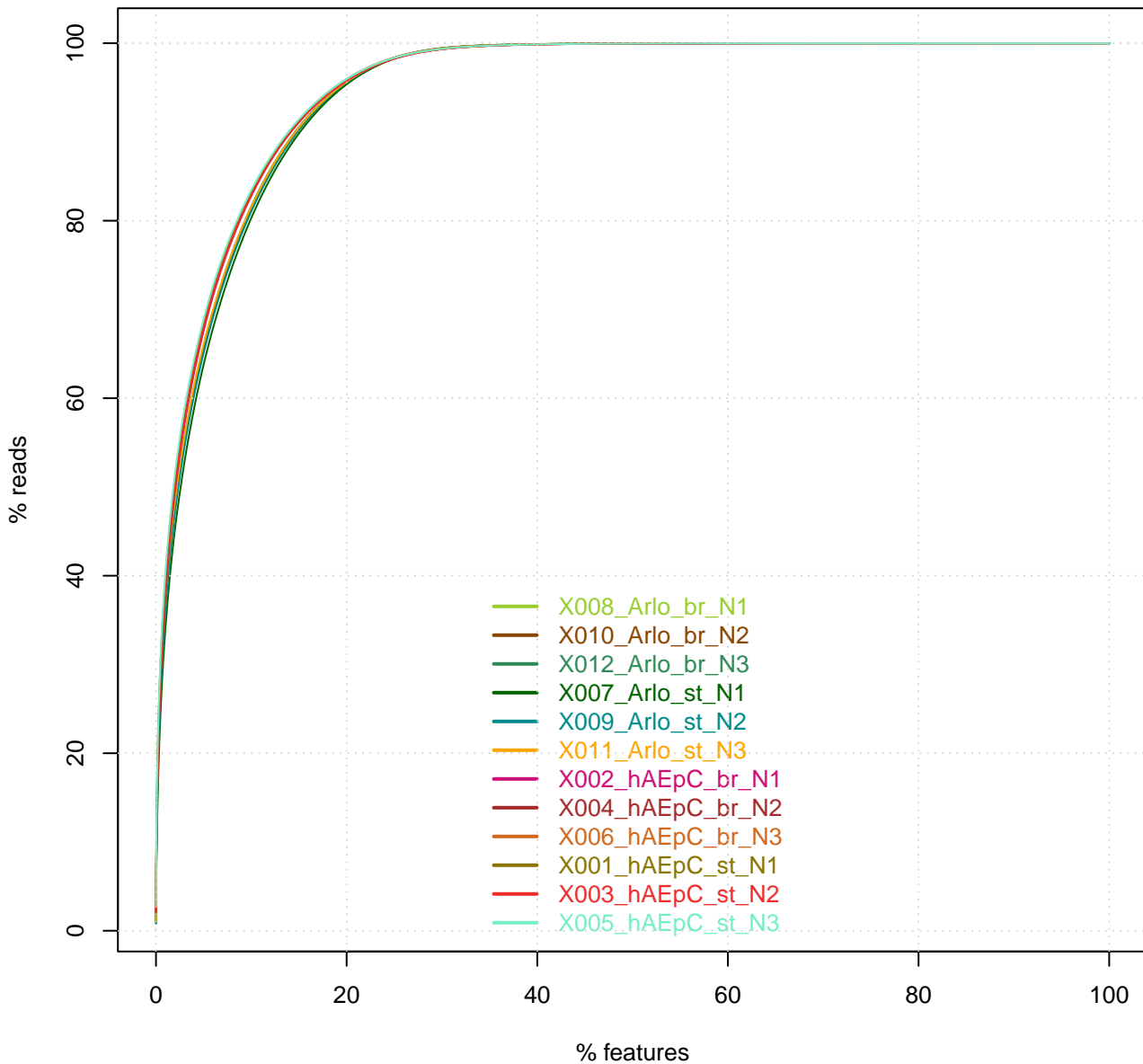

Supplement: Supplementary file 2 — Data S2: Supporting Information [file BTM2-10-e10715-s002.zip › plots/qc/readnoise.pdf]

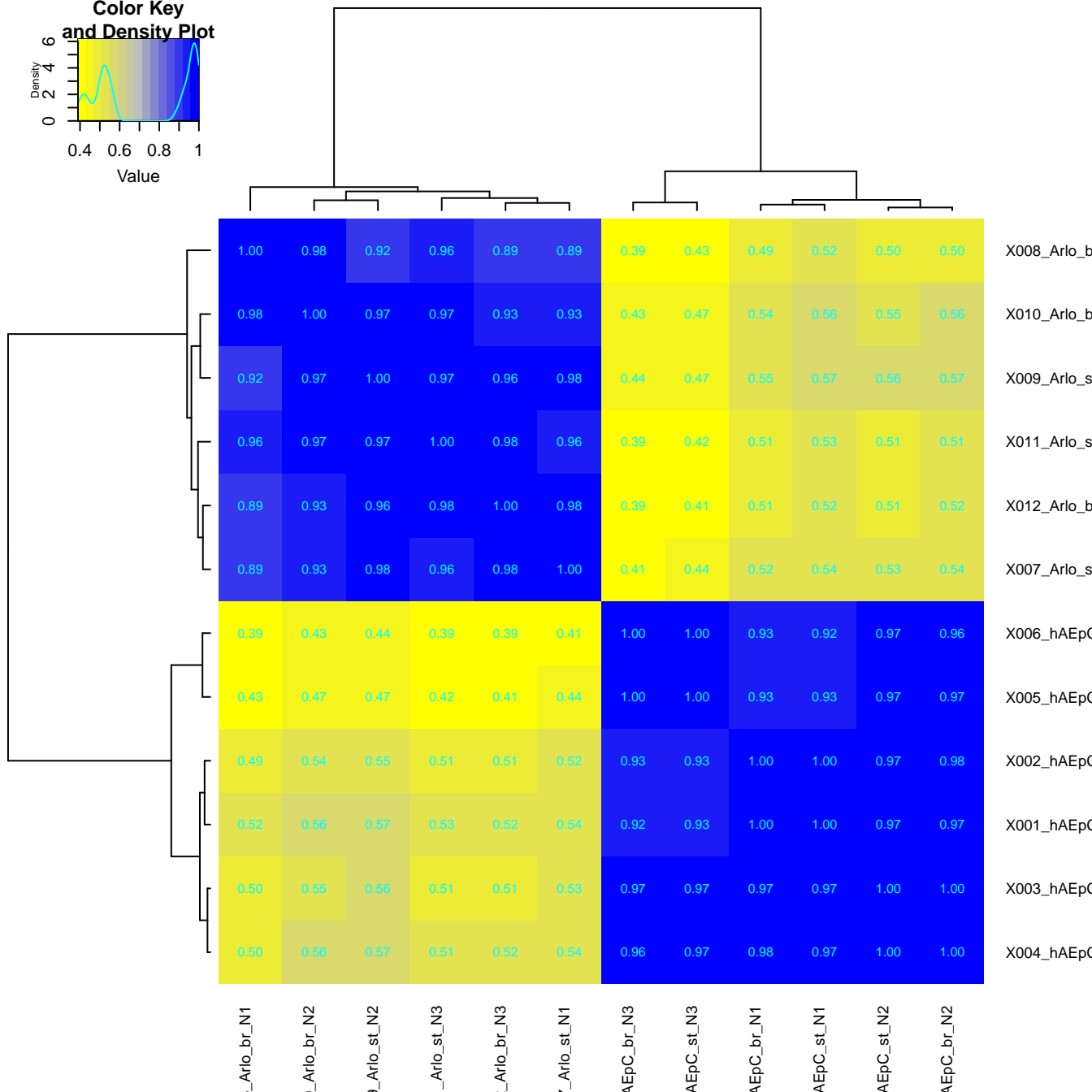

Supplement: Supplementary file 2 — Data S2: Supporting Information [file BTM2-10-e10715-s002.zip › plots/qc/correlation_heatmap.pdf]

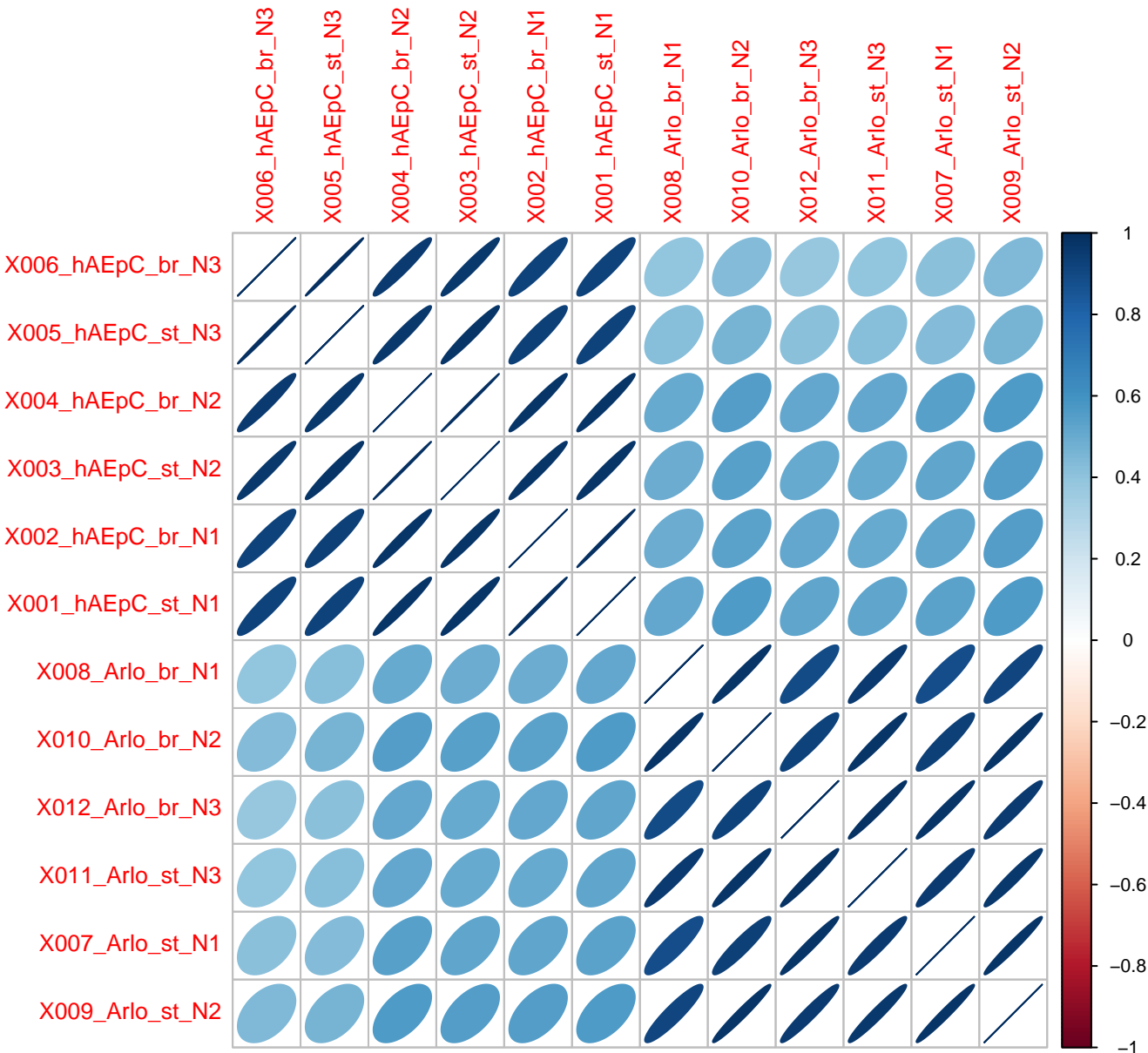

Supplement: Supplementary file 2 — Data S2: Supporting Information [file BTM2-10-e10715-s002.zip › plots/qc/correlation_correlogram.pdf]

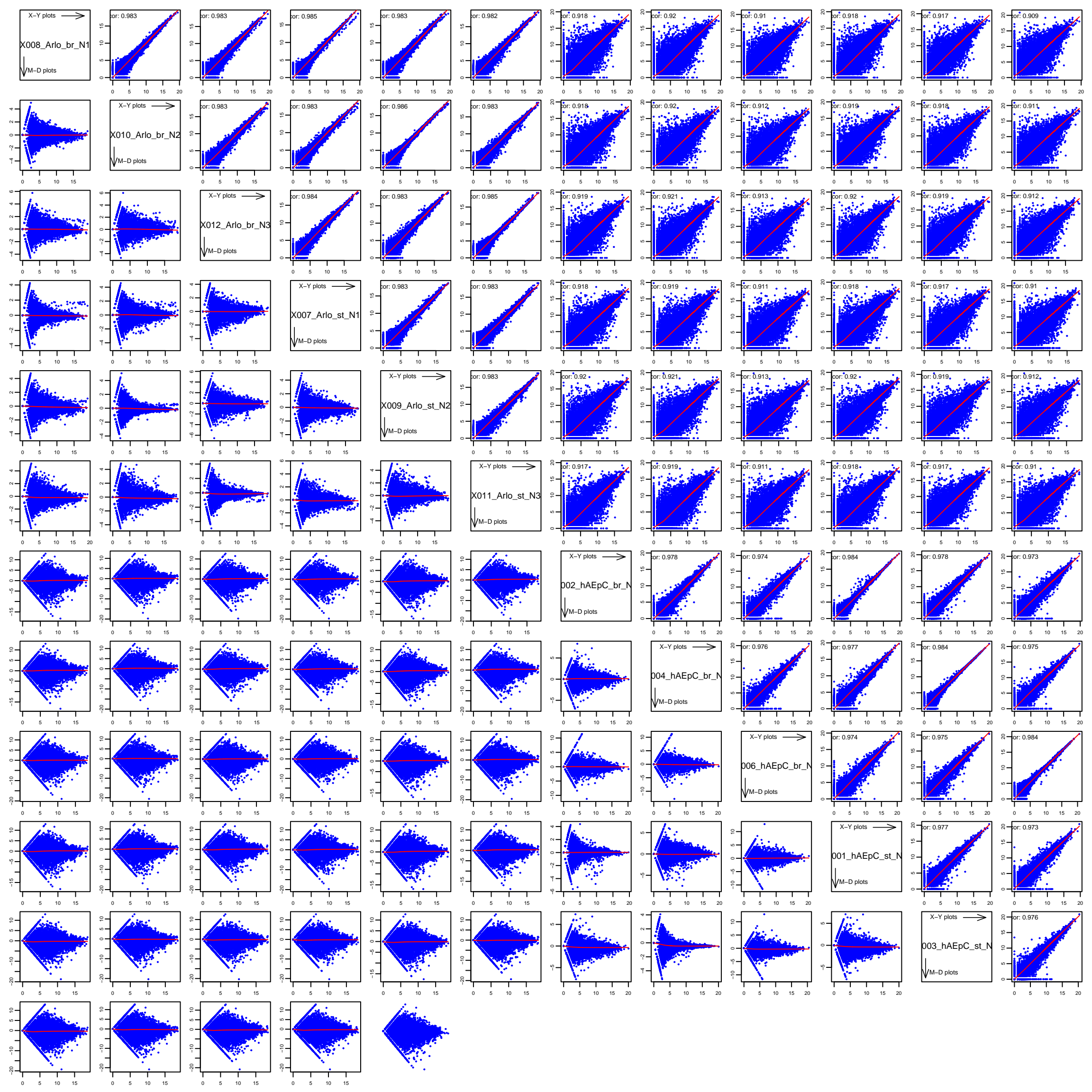

Supplement: Supplementary file 2 — Data S2: Supporting Information [file BTM2-10-e10715-s002.zip › plots/qc/correlation_pairs.pdf]

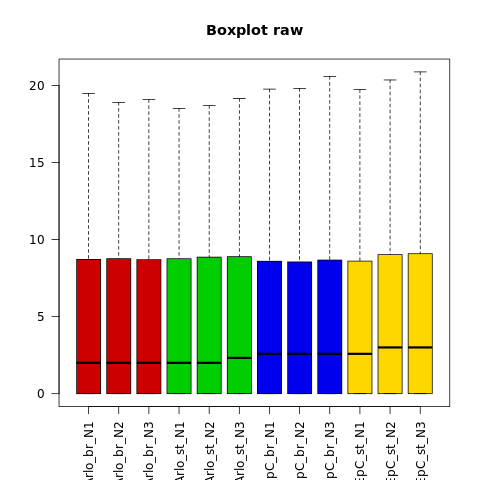

Supplement: Supplementary file 2 — Data S2: Supporting Information [file BTM2-10-e10715-s002.zip › plots/normalization/boxplot_raw.png]

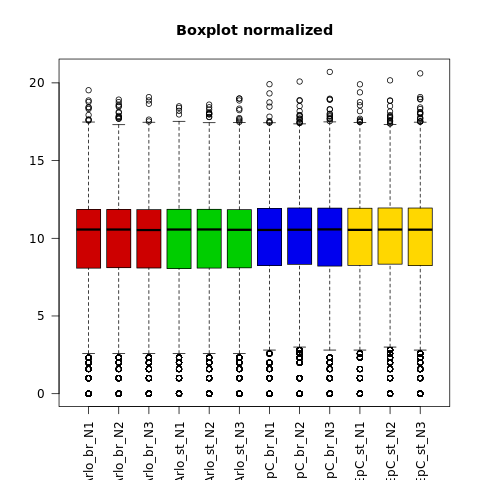

Supplement: Supplementary file 2 — Data S2: Supporting Information [file BTM2-10-e10715-s002.zip › plots/normalization/boxplot_normalized.png]

Boxplot raw

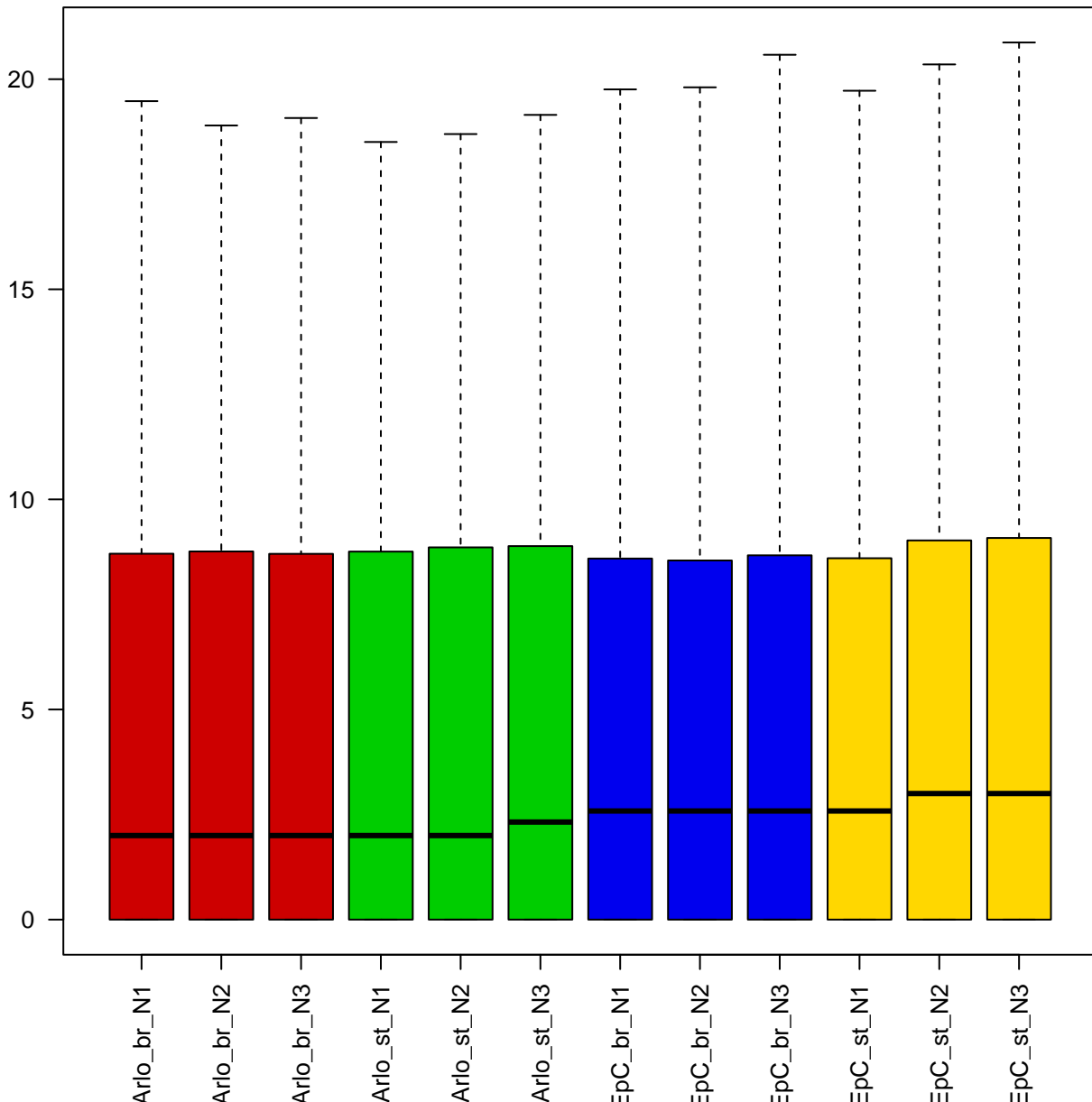

Supplement: Supplementary file 2 — Data S2: Supporting Information [file BTM2-10-e10715-s002.zip › plots/normalization/boxplot_raw.pdf]

Boxplot normalized

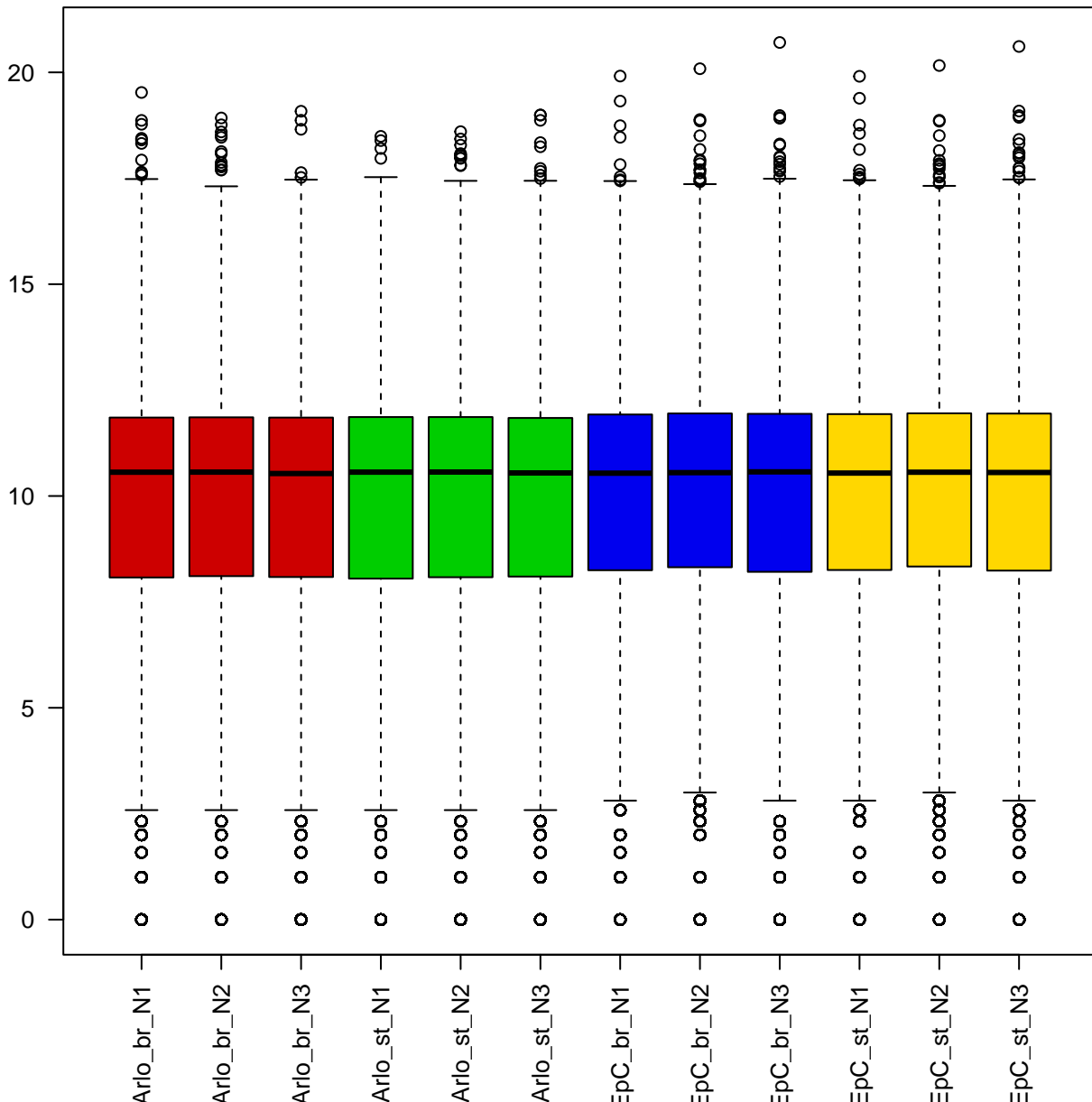

Supplement: Supplementary file 2 — Data S2: Supporting Information [file BTM2-10-e10715-s002.zip › plots/normalization/boxplot_normalized.pdf]

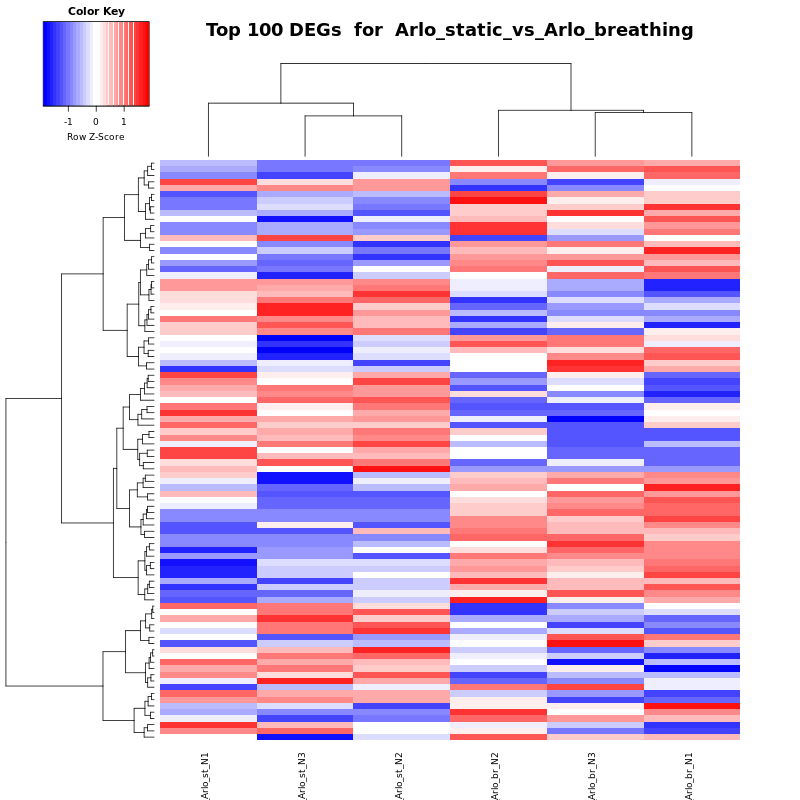

Supplement: Supplementary file 2 — Data S2: Supporting Information [file BTM2-10-e10715-s002.zip › plots/statistics/de_heatmap_Arlo_static_vs_Arlo_breathing.png]

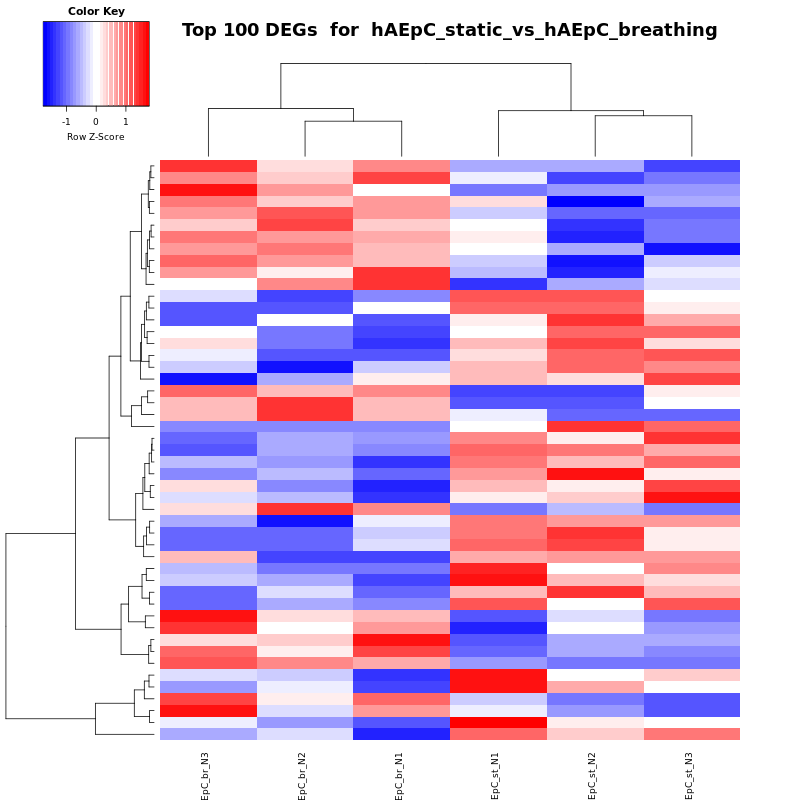

Supplement: Supplementary file 2 — Data S2: Supporting Information [file BTM2-10-e10715-s002.zip › plots/statistics/de_heatmap_hAEpC_static_vs_hAEpC_breathing.png]

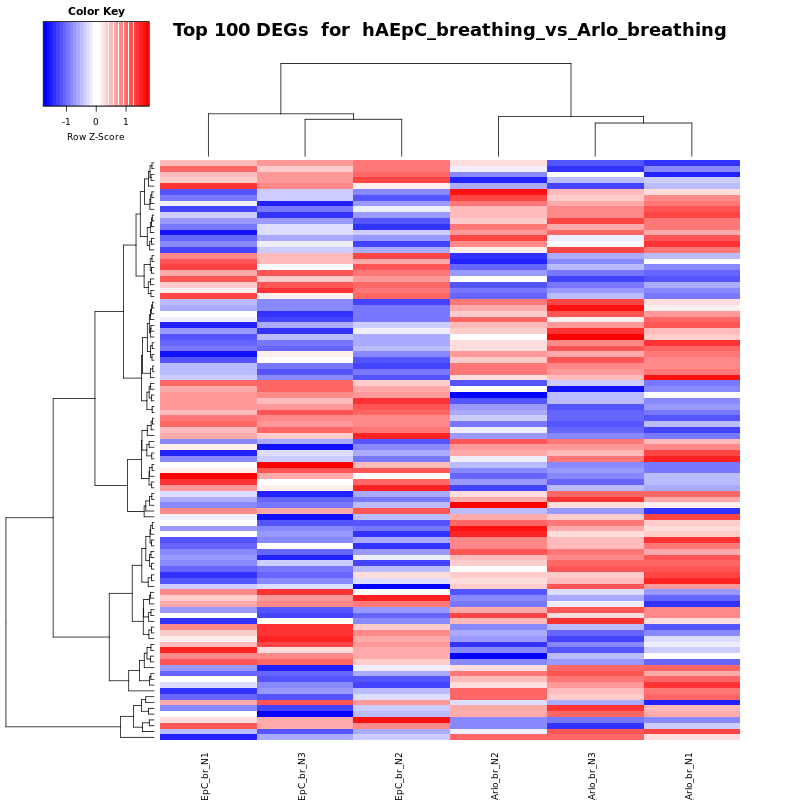

Supplement: Supplementary file 2 — Data S2: Supporting Information [file BTM2-10-e10715-s002.zip › plots/statistics/de_heatmap_hAEpC_breathing_vs_Arlo_breathing.png]

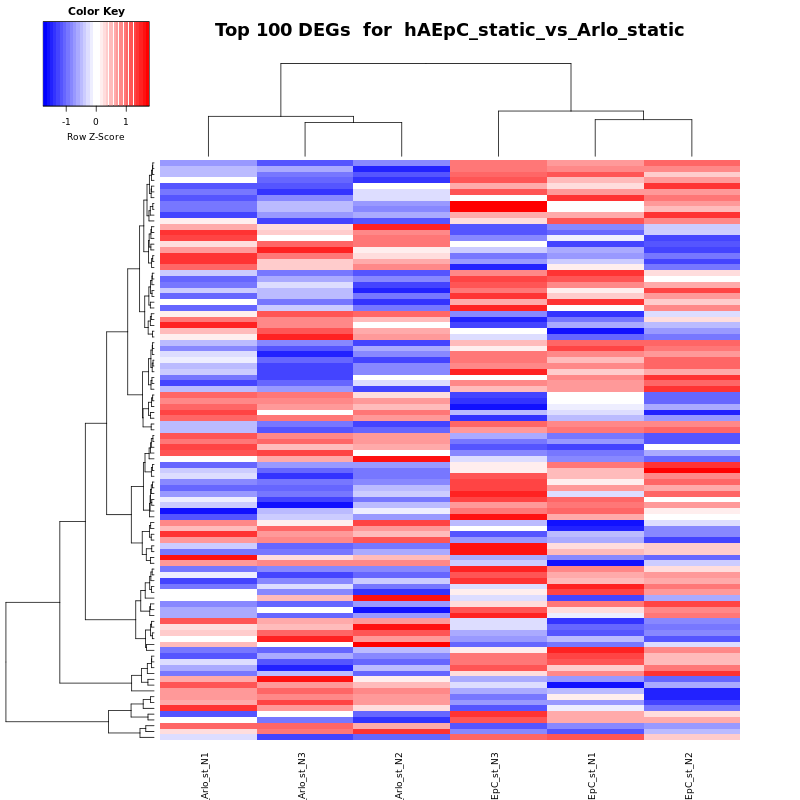

Supplement: Supplementary file 2 — Data S2: Supporting Information [file BTM2-10-e10715-s002.zip › plots/statistics/de_heatmap_hAEpC_static_vs_Arlo_static.png]

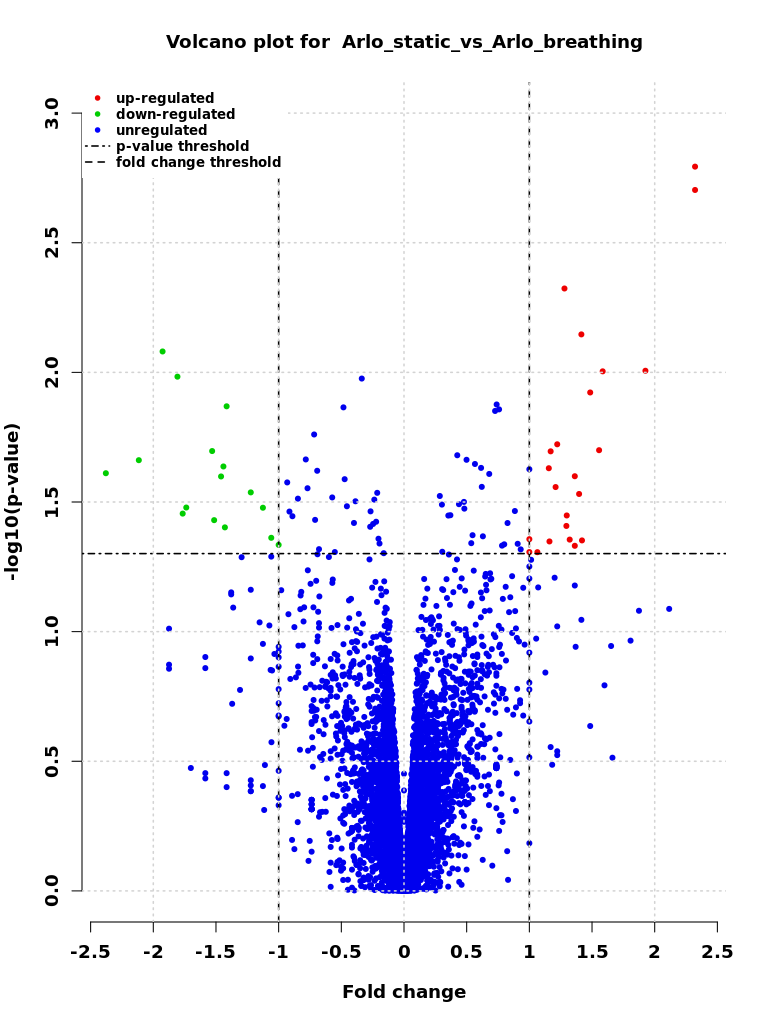

Supplement: Supplementary file 2 — Data S2: Supporting Information [file BTM2-10-e10715-s002.zip › plots/statistics/volcano_plot_Arlo_static_vs_Arlo_breathing.png]

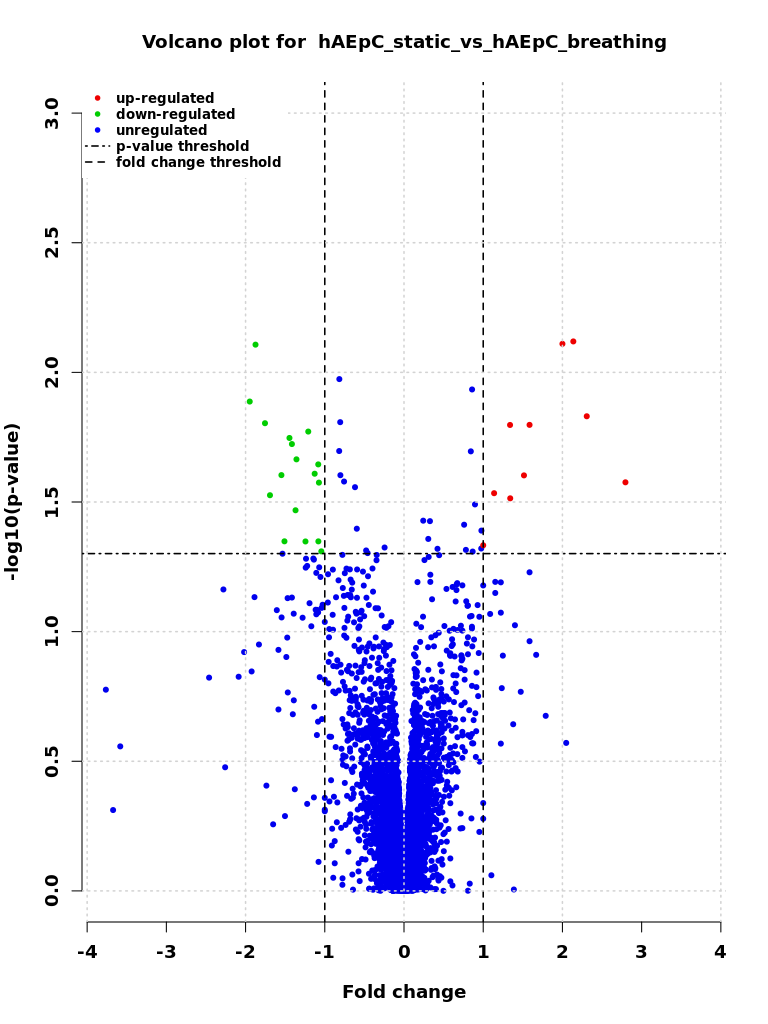

Supplement: Supplementary file 2 — Data S2: Supporting Information [file BTM2-10-e10715-s002.zip › plots/statistics/volcano_plot_hAEpC_static_vs_hAEpC_breathing.png]

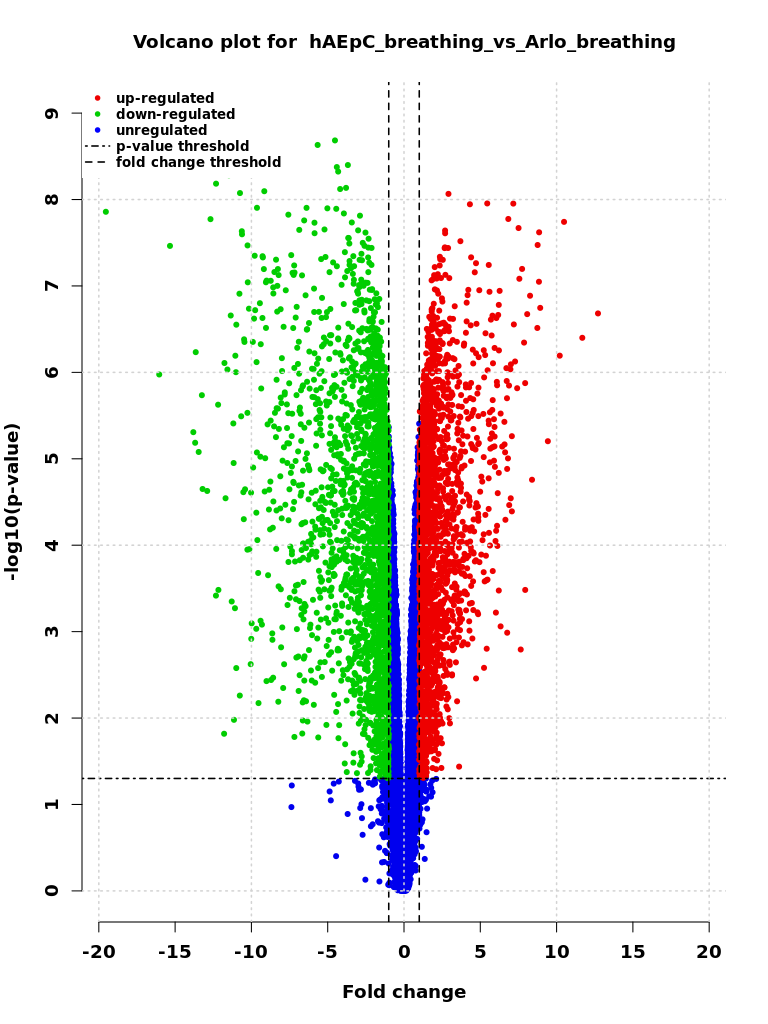

Supplement: Supplementary file 2 — Data S2: Supporting Information [file BTM2-10-e10715-s002.zip › plots/statistics/volcano_plot_hAEpC_breathing_vs_Arlo_breathing.png]

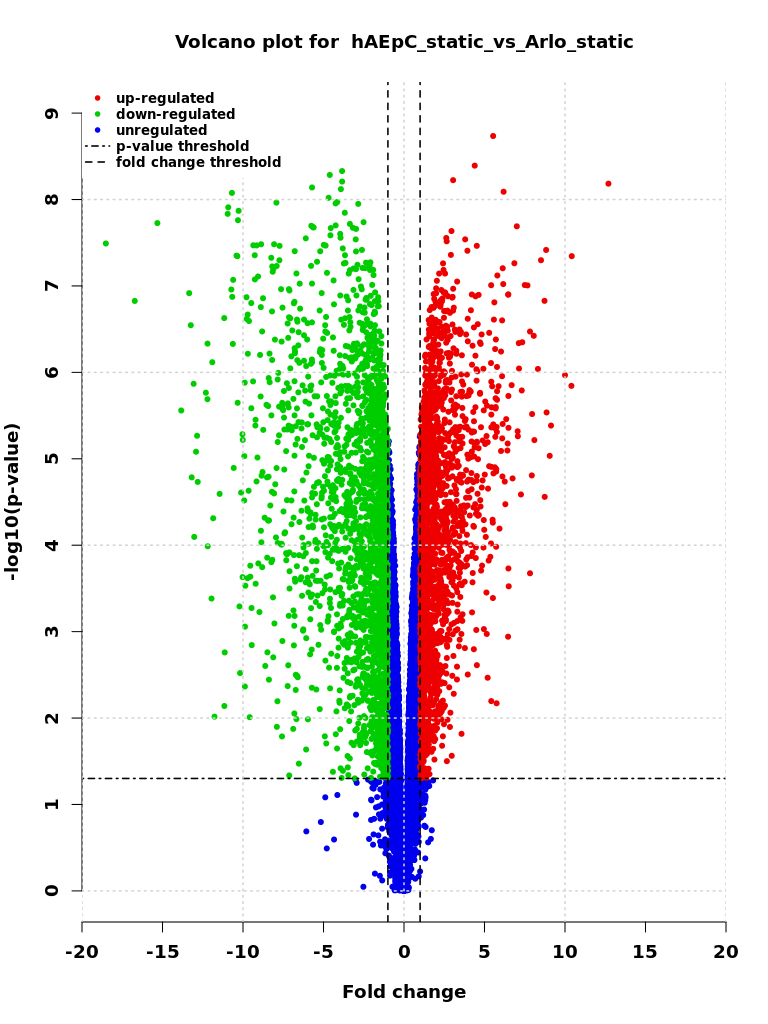

Supplement: Supplementary file 2 — Data S2: Supporting Information [file BTM2-10-e10715-s002.zip › plots/statistics/volcano_plot_hAEpC_static_vs_Arlo_static.png]

Color Key

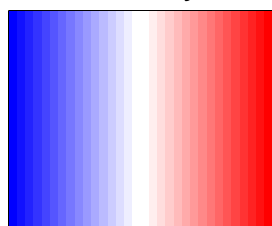

-1 0 1

Row Z-Score

# Top 100 DEGs for Arlo\_static\_vs\_Arlo\_breathing

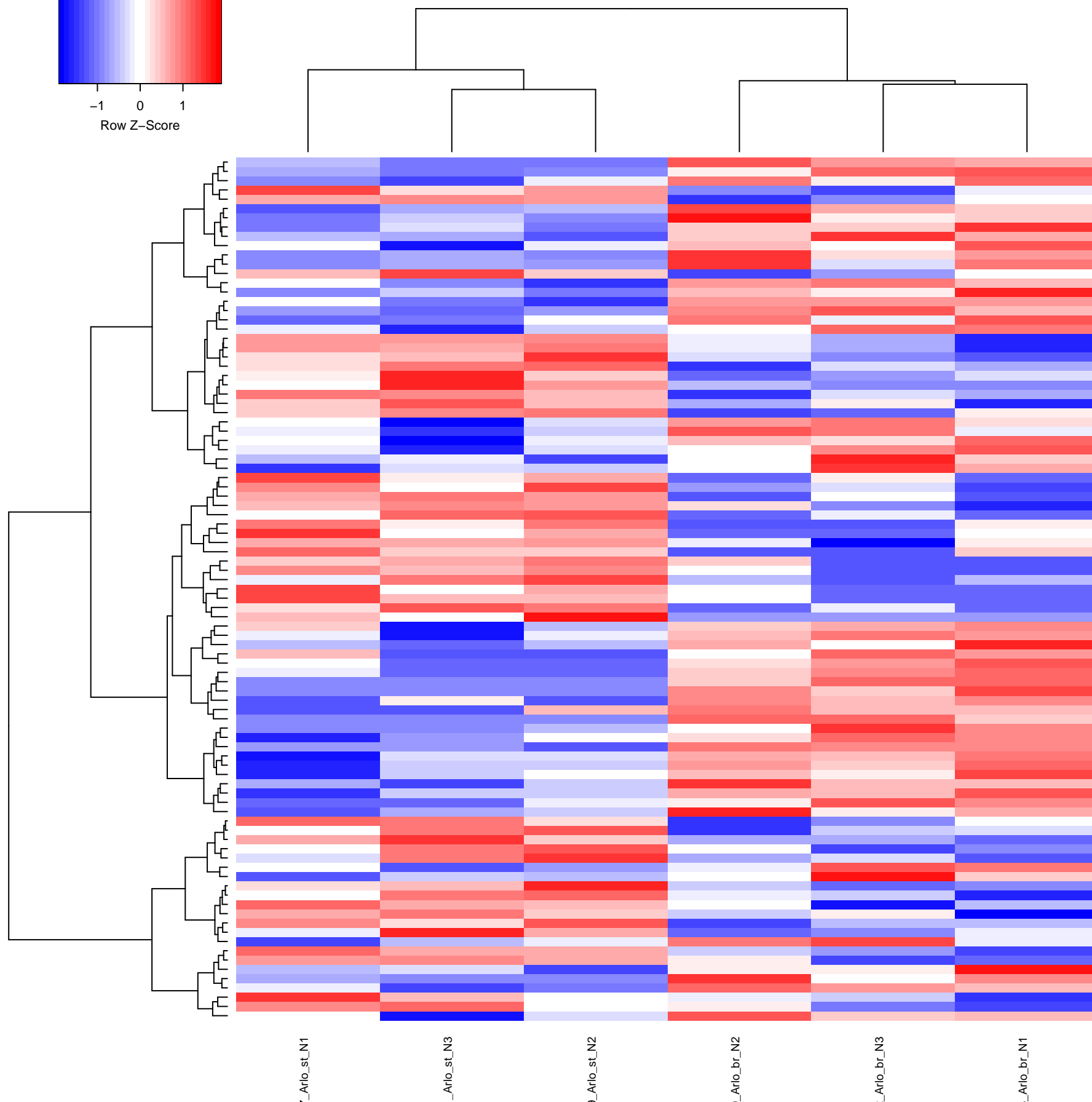

Supplement: Supplementary file 2 — Data S2: Supporting Information [file BTM2-10-e10715-s002.zip › plots/statistics/de_heatmap_Arlo_static_vs_Arlo_breathing.pdf]

Color Key

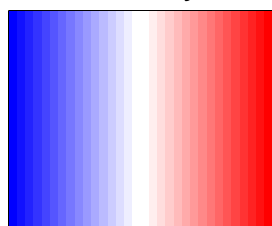

-1 0 1

Row Z-Score

Top 100 DEGs for hAEpC\_static\_vs\_hAEpC\_breathing

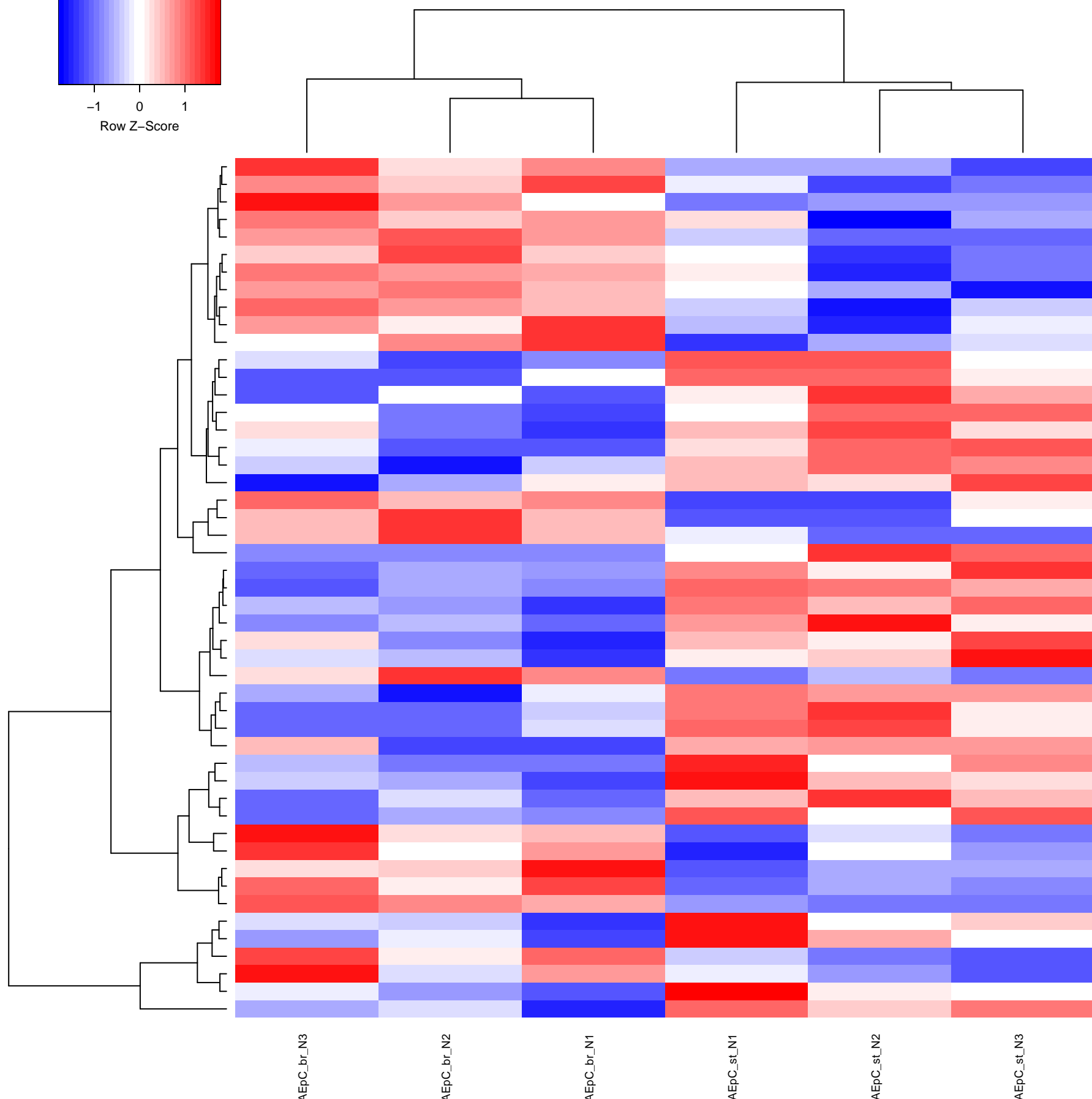

Supplement: Supplementary file 2 — Data S2: Supporting Information [file BTM2-10-e10715-s002.zip › plots/statistics/de_heatmap_hAEpC_static_vs_hAEpC_breathing.pdf]

Color Key

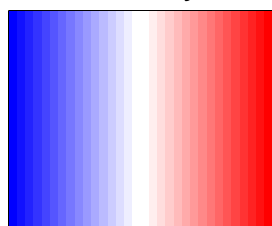

-1 0 1

Row Z-Score

# Top 100 DEGs for hAEpC\_breathing\_vs\_Arlo\_breathing

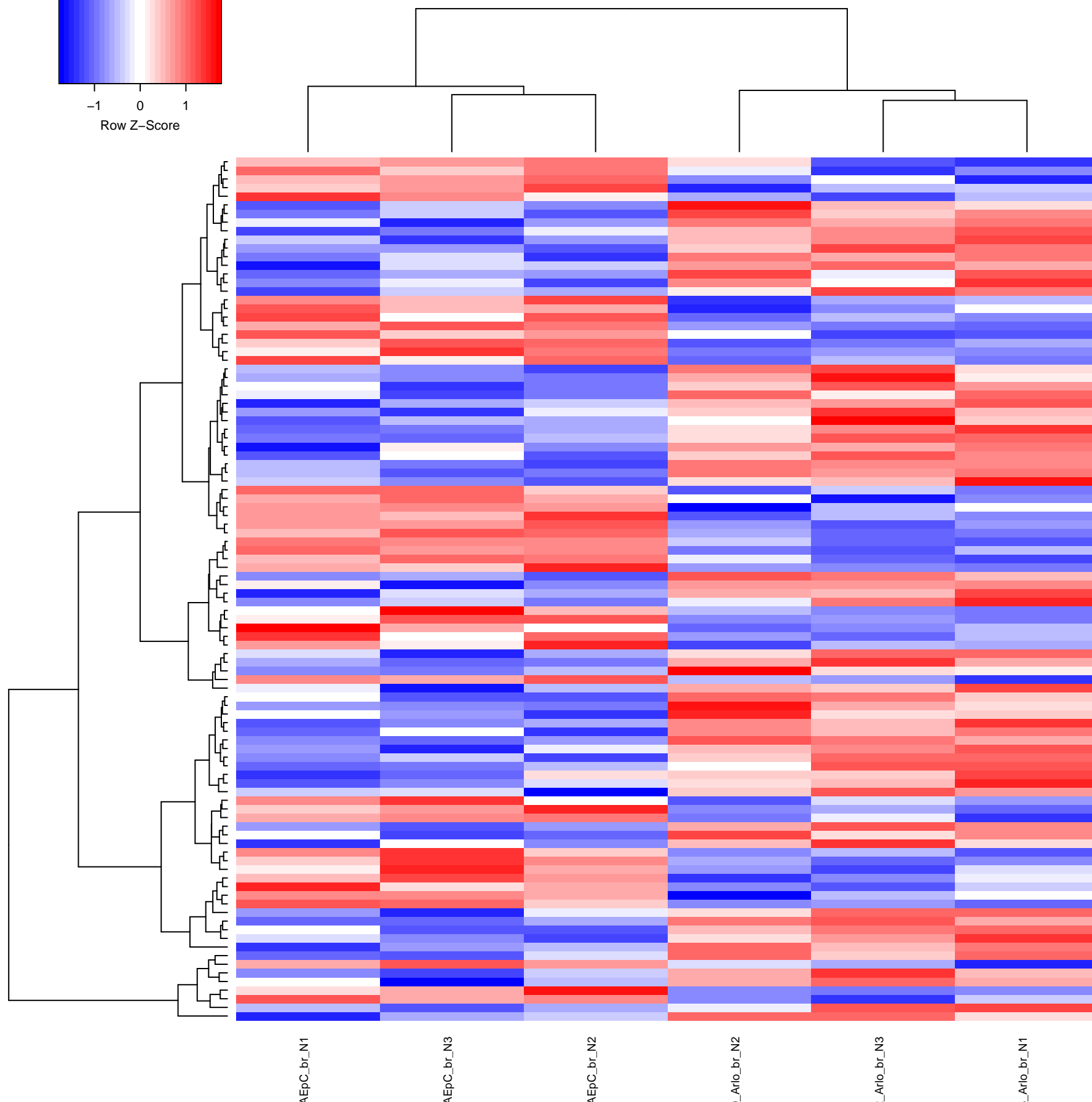

Supplement: Supplementary file 2 — Data S2: Supporting Information [file BTM2-10-e10715-s002.zip › plots/statistics/de_heatmap_hAEpC_breathing_vs_Arlo_breathing.pdf]

Color Key

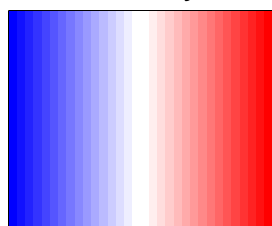

-1 0 1

Row Z-Score

# Top 100 DEGs for hAEpC\_static\_vs\_Arlo\_static

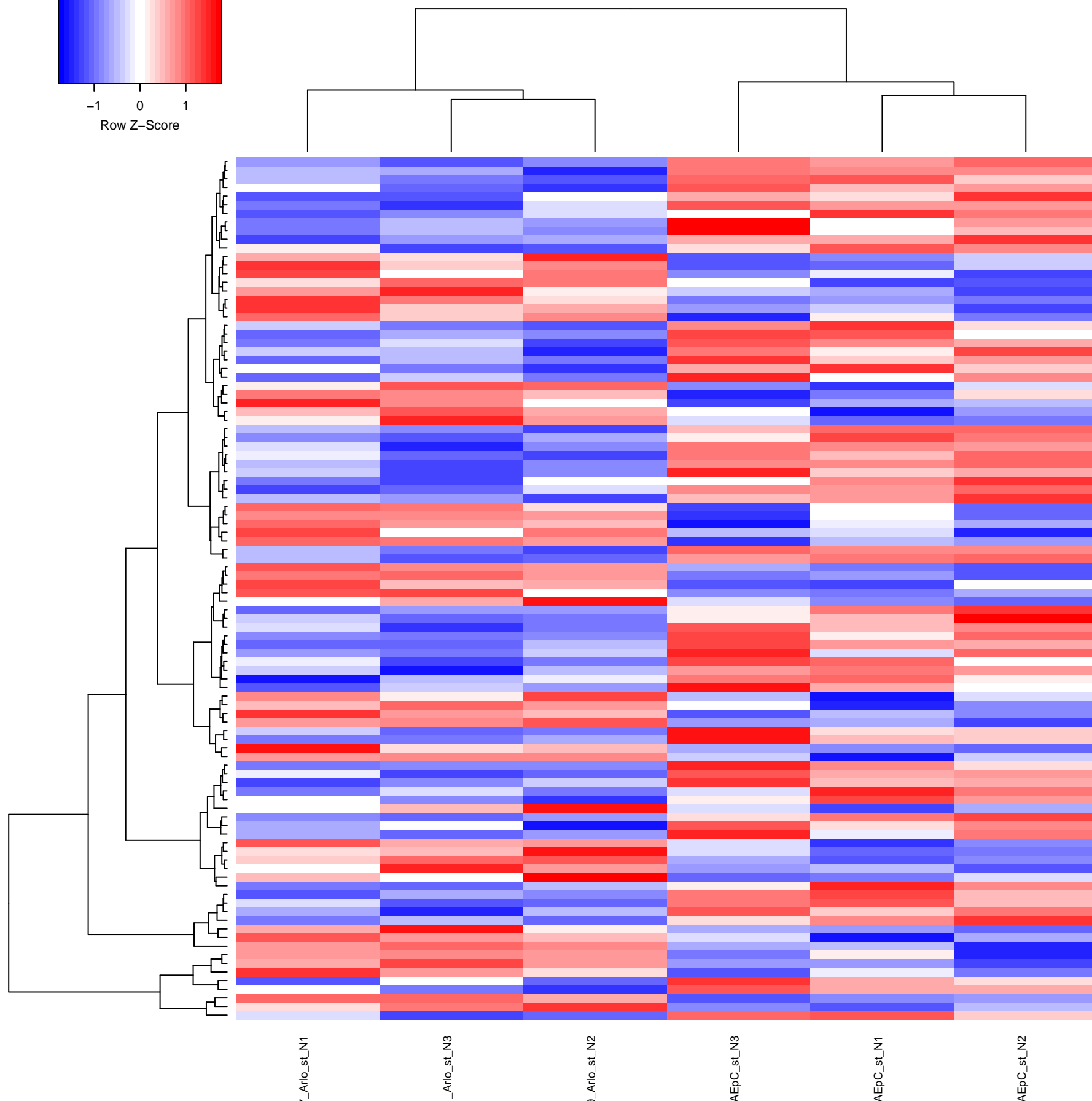

Supplement: Supplementary file 2 — Data S2: Supporting Information [file BTM2-10-e10715-s002.zip › plots/statistics/de_heatmap_hAEpC_static_vs_Arlo_static.pdf]

Volcano plot for Arlo\_static\_vs\_Arlo\_breathing

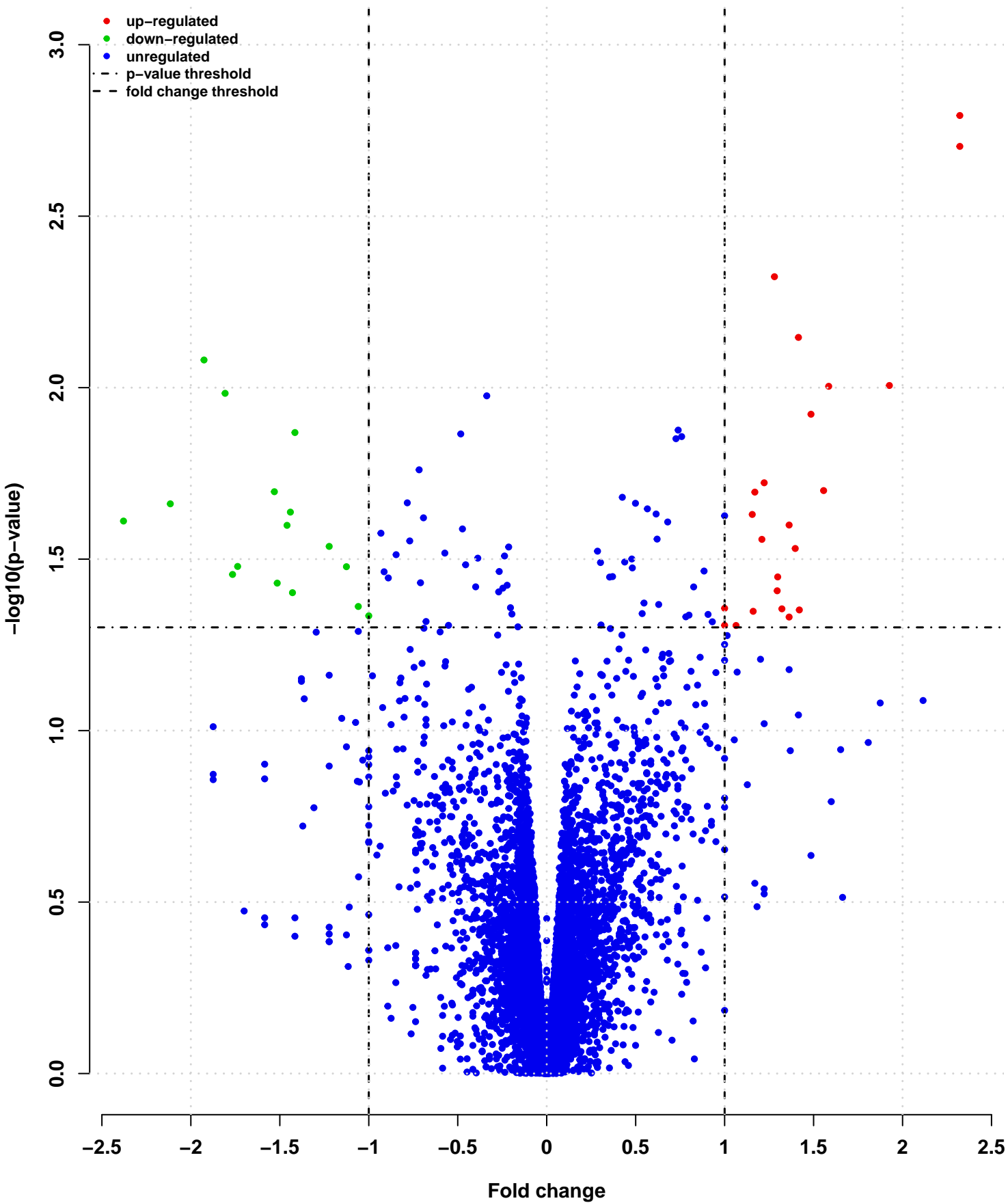

Supplement: Supplementary file 2 — Data S2: Supporting Information [file BTM2-10-e10715-s002.zip › plots/statistics/volcano_plot_Arlo_static_vs_Arlo_breathing.pdf]

Volcano plot for hAEpC\_static\_vs\_hAEpC\_breathing

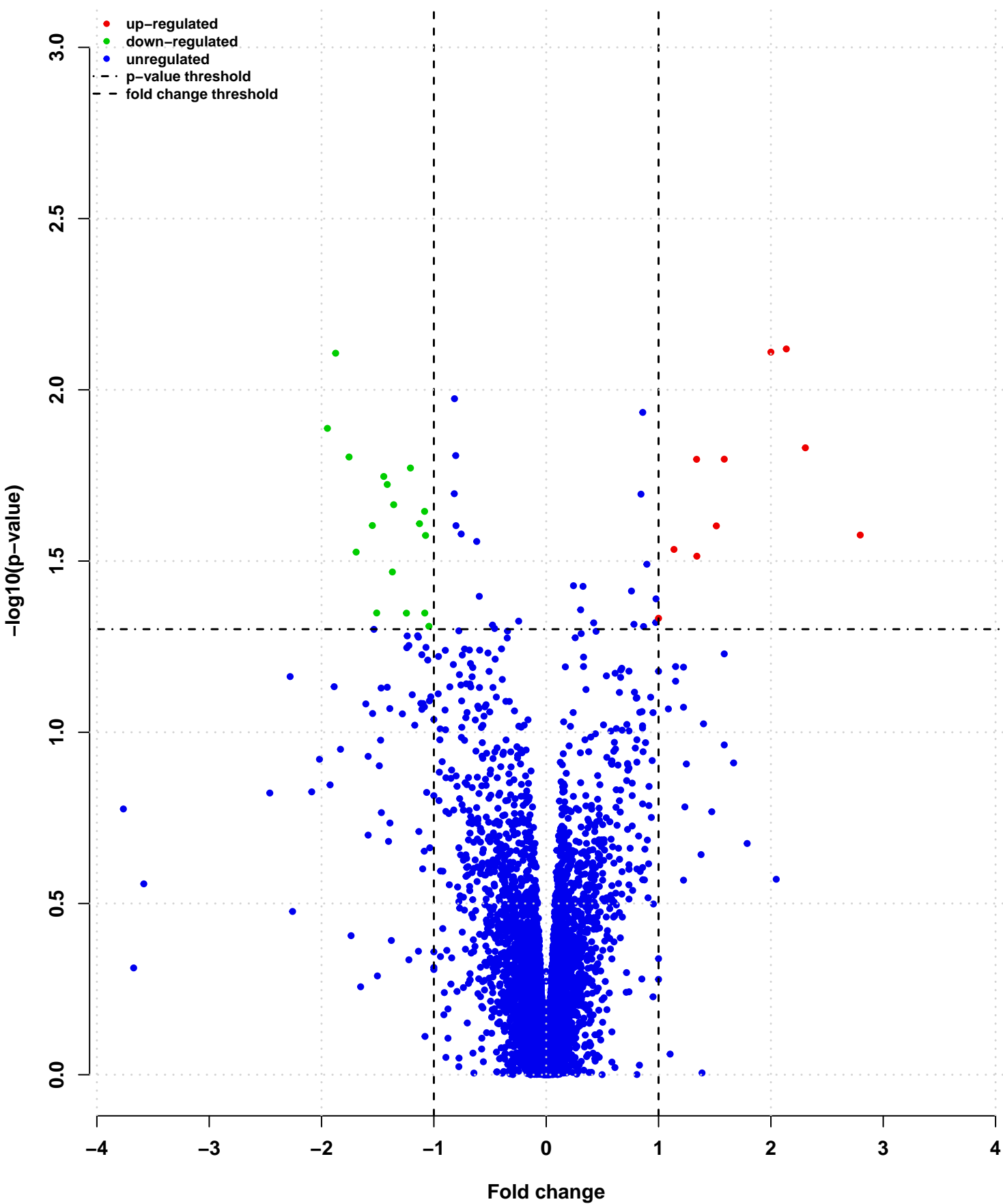

Supplement: Supplementary file 2 — Data S2: Supporting Information [file BTM2-10-e10715-s002.zip › plots/statistics/volcano_plot_hAEpC_static_vs_hAEpC_breathing.pdf]

Volcano plot for hAEpC\_breathing\_vs\_Arlo\_breathing

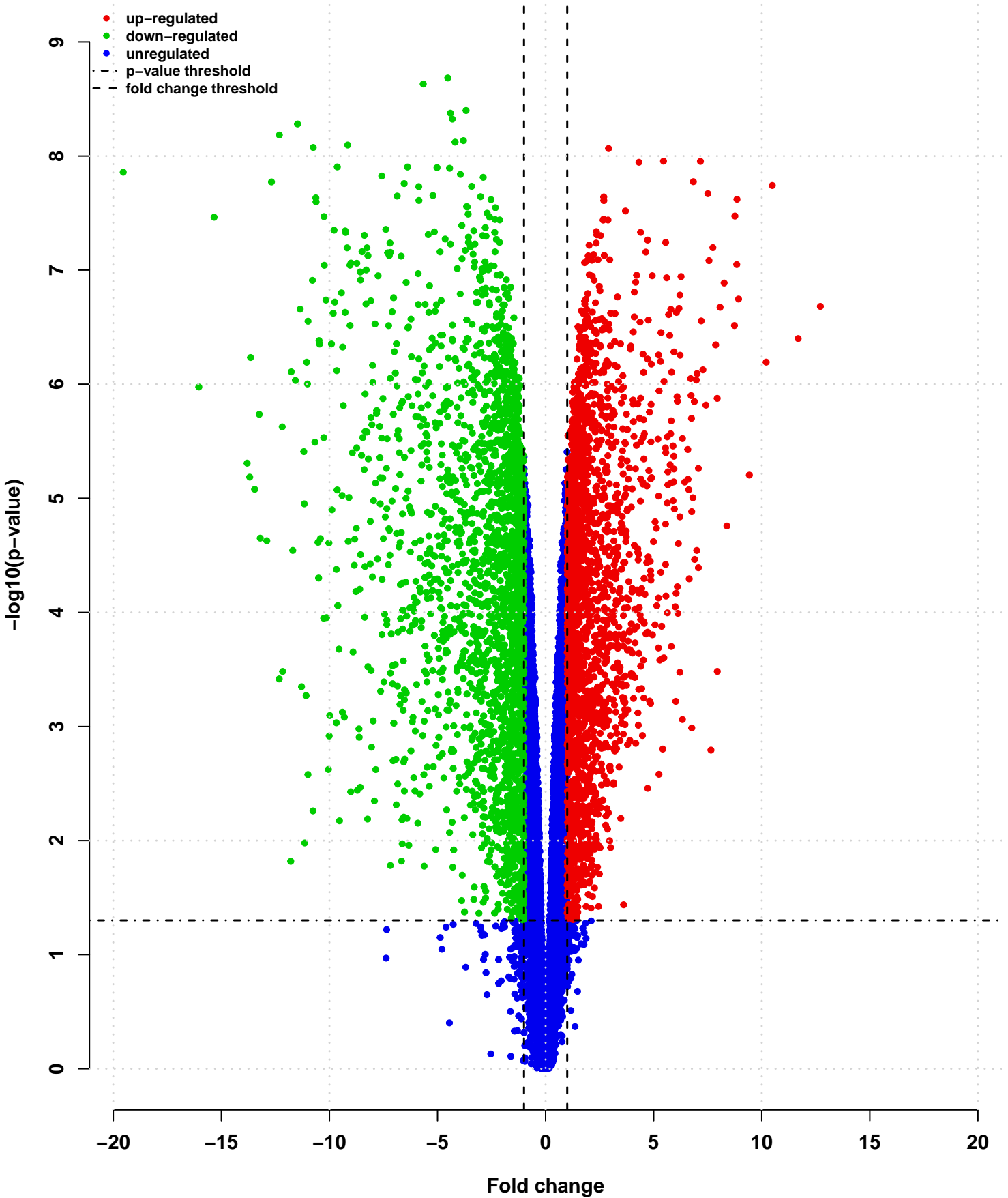

Supplement: Supplementary file 2 — Data S2: Supporting Information [file BTM2-10-e10715-s002.zip › plots/statistics/volcano_plot_hAEpC_breathing_vs_Arlo_breathing.pdf]

Volcano plot for hAEpC\_static\_vs\_Arlo\_static

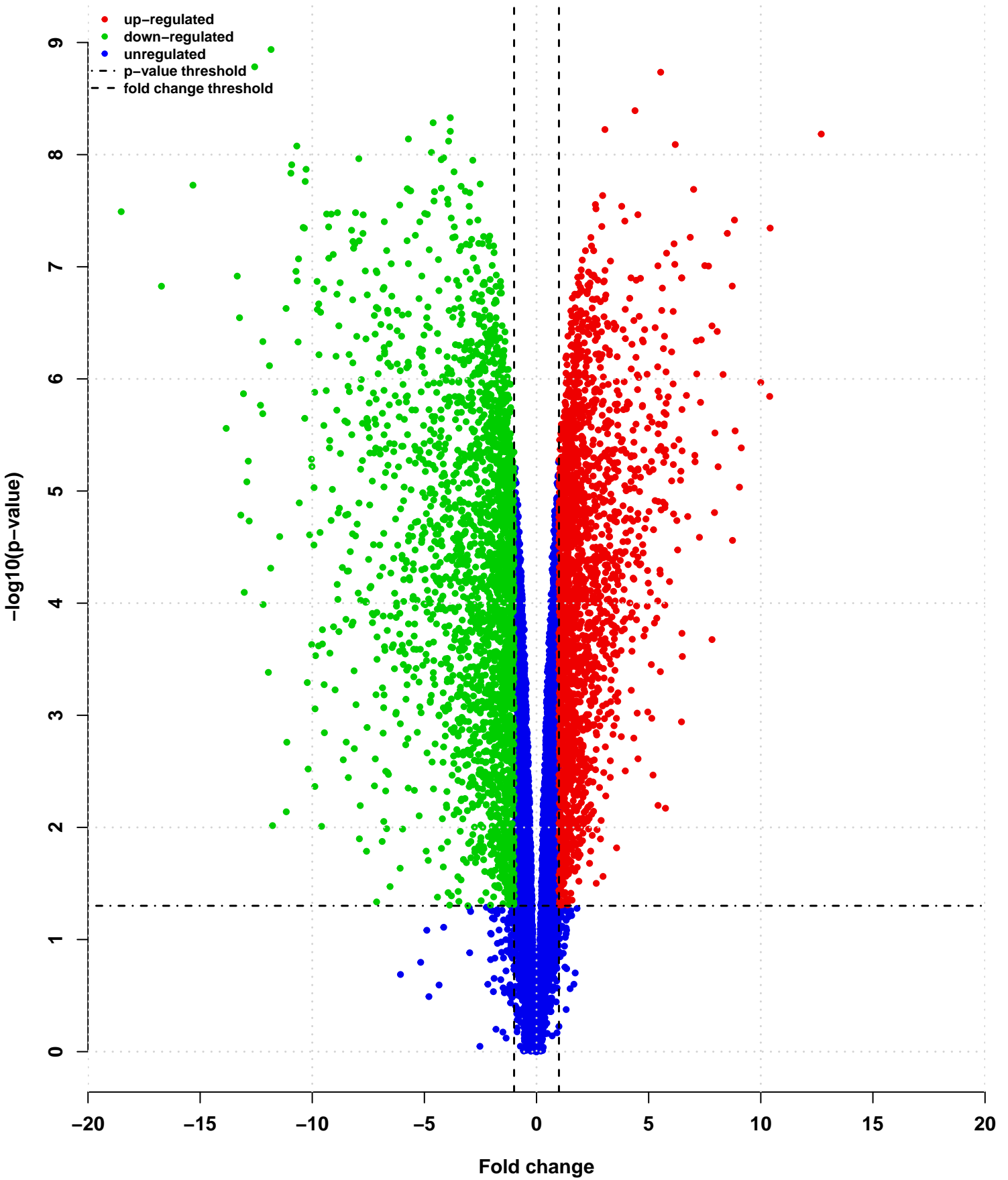

Supplement: Supplementary file 2 — Data S2: Supporting Information [file BTM2-10-e10715-s002.zip › plots/statistics/volcano_plot_hAEpC_static_vs_Arlo_static.pdf]

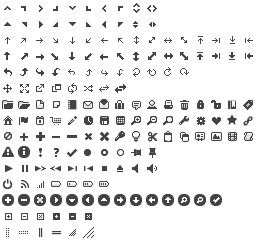

Supplement: Supplementary file 2 — Data S2: Supporting Information [file BTM2-10-e10715-s002.zip › libs/jqueryui-1.11.4/images/ui-icons_444444_256x240.png]

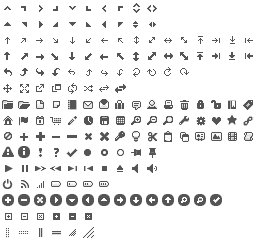

Supplement: Supplementary file 2 — Data S2: Supporting Information [file BTM2-10-e10715-s002.zip › libs/jqueryui-1.11.4/images/ui-icons_555555_256x240.png]

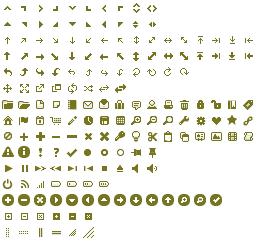

Supplement: Supplementary file 2 — Data S2: Supporting Information [file BTM2-10-e10715-s002.zip › libs/jqueryui-1.11.4/images/ui-icons_777620_256x240.png]

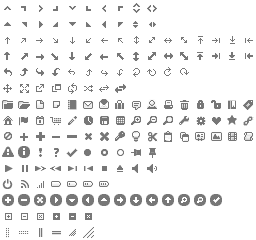

Supplement: Supplementary file 2 — Data S2: Supporting Information [file BTM2-10-e10715-s002.zip › libs/jqueryui-1.11.4/images/ui-icons_777777_256x240.png]

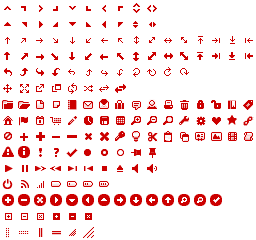

Supplement: Supplementary file 2 — Data S2: Supporting Information [file BTM2-10-e10715-s002.zip › libs/jqueryui-1.11.4/images/ui-icons_cc0000_256x240.png]

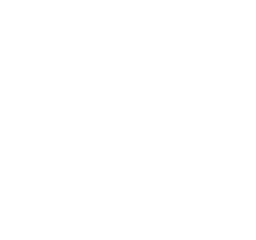

Supplement: Supplementary file 2 — Data S2: Supporting Information [file BTM2-10-e10715-s002.zip › libs/jqueryui-1.11.4/images/ui-icons_ffffff_256x240.png]
